# Experimental and Computational Studies of Ruthenium Complexes Bearing *Z*-Acceptor Aluminum-Based Phosphine Pincer Ligands

**DOI:** 10.1021/acs.inorgchem.2c03665

**Published:** 2022-12-07

**Authors:** Connie J. Isaac, Cameron I. Wilson, Arron L. Burnage, Fedor M. Miloserdov, Mary F. Mahon, Stuart A. Macgregor, Michael K. Whittlesey

**Affiliations:** † Department of Chemistry, University of Bath, Bath BA2 7AY, U.K.; ‡ Institute of Chemical Sciences, Heriot-Watt University, Edinburgh EH14 4AS, U.K.

## Abstract

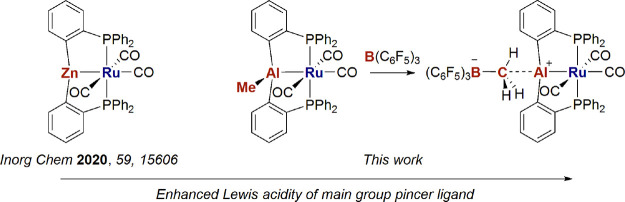

Reaction of [Ru(C_6_H_4_PPh_2_)_2_(Ph_2_PC_6_H_4_AlMe(THF))H] with CO results in clean conversion to the Ru−Al heterobimetallic complex [Ru(AlMePhos)(CO)_3_] (**1**), where AlMePhos is the novel P–Al(Me)–P pincer ligand (*o*-Ph_2_PC_6_H_4_)_2_AlMe. Under photolytic conditions, **1** reacts with H_2_ to give [Ru(AlMePhos)(CO)_2_(μ-H)H] (**2**) that is characterized by multinuclear NMR and IR spectroscopies. DFT calculations indicate that **2** features one terminal and one bridging hydride that are respectively *anti* and *syn* to the Al*Me* group. Calculations also define a mechanism for H_2_ addition to **1** and predict facile hydride exchange in **2** that is also observed experimentally. Reaction of **1** with B(C_6_F_5_)_3_ results in Me abstraction to form the ion pair [Ru(AlPhos)(CO)_3_][MeB(C_6_F_5_)_3_] (**4**) featuring a cationic [(*o*-Ph_2_PC_6_H_4_)_2_Al]^+^ ligand, [AlPhos]^+^. The Ru–Al distance in **4** (2.5334(16) Å) is significantly shorter than that in **1** (2.6578(6) Å), consistent with an enhanced Lewis acidity of the [AlPhos]^+^ ligand. This is corroborated by a blue shift in both the observed and computed ν_CO_ stretching frequencies upon Me abstraction. Electronic structure analyses (QTAIM and EDA-ETS) comparing **1**, **4**, and the previously reported [Ru(ZnPhos)(CO)_3_] analogue (ZnPhos = (*o*-Ph_2_PC_6_H_4_)_2_Zn) indicate that the Lewis acidity of these pincer ligands increases along the series ZnPhos < AlMePhos < [AlPhos]^+^.

## Abstract 2

A combination of experimental and computational approaches shows that the Lewis acidities of the novel *Z*-acceptor pincer phosphine ligands (*o*-Ph_2_PC_6_H_4_)_2_AlMe (AlMePhos) and [(*o*-Ph_2_PC_6_H_4_)_2_Al]^+^ (AlPhos) in [Ru(AlMePhos)(CO)_3_] and [Ru(AlPhos)(CO)_3_]^+^ respectively are greater than those of the previously reported Zn analogue (*o*-Ph_2_PC_6_H_4_)_2_Zn (ZnPhos).

## Introduction

Sigma-accepting (or *Z*-type) ligands incorporating Lewis acidic E(*X*)_n_ functionalities have become prominent in the past few years because of their ability to interact with transition metal (TM) centers to afford complexes with unusual coordination geometries and high reactivity.^1,2^ One commonly used approach to stabilize TM → E(*X*)_n_ interactions involves the use of peripheral P donors to form pincer phosphine ligands P–E(*X*)_n_–P.^3−6^ As the archetypal Lewis acids, group 13 elements, and in particular B, have been the focus of considerable attention and a rich chemistry has developed for B(alkyl/aryl)-derived pincers.^7−9^ In contrast, far fewer examples of P–Al(X)–P ligands are known, and these are largely restricted to X = halide derivatives.^10−14^ In one early example, Bourissou and co-workers showed that attempts to generate Cu→(P–Al(Cl)–P) and Au→(P–Al(Cl)–P) complexes through coordination of (*o*-^i^Pr_2_PC_6_H_4_)_2_AlCl to Cu(I) and Au(I) halide precursors instead resulted in halide migration from the coinage metal to Al to afford zwitterionic products as a result of the high Lewis acidity of the AlCl moiety.^10,11^

**Scheme 1:**
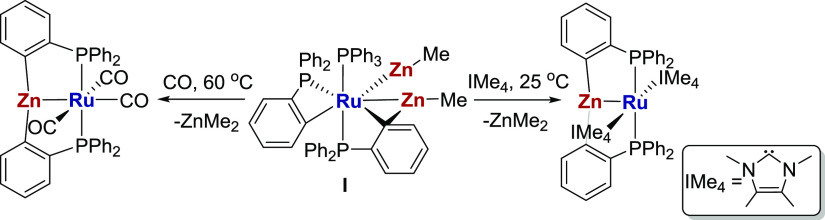
Synthesis of [Ru(ZnPhos)] Complexes from [Ru(PPh_3_)(C_6_H_4_PPh_2_)_2_(ZnMe)_2_] (**I**)

**Scheme 2:**
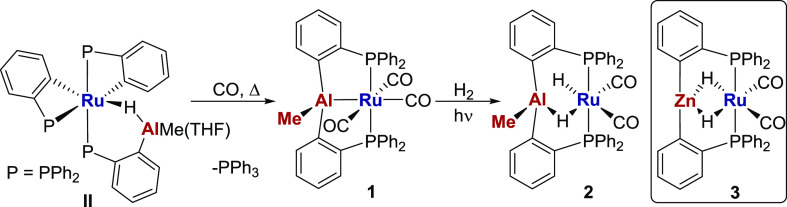
Synthesis of [Ru(AlMePhos)(CO)_3_] (**1**) and Reaction with H_2_ to Give **2** The structure of **2** is drawn on the basis of combined NMR and computational evidence discussed in the main text.

In a recent report, we described the serendipitous formation and trapping of the novel Zn-based *Z*-acceptor pincer ligand (*o*-Ph_2_PC_6_H_4_)_2_Zn (abbreviated to ZnPhos) following reaction of the heterobimetallic ruthenium-zinc complex [Ru(PPh_3_)(C_6_H_4_PPh_2_)_2_(ZnMe)_2_] (**I**) with CO or an N-heterocyclic carbene (Scheme 1).^15,16^ The precise steps leading to the formation of the ZnPhos ligand are not known, but the presence of two cyclometalated phosphine ligands able to couple onto the Lewis acidic Zn center appears to be one requirement. In accord with this, we now report the formation of the corresponding AlMePhos (*o*-Ph_2_PC_6_H_4_)_2_AlMe; Scheme 2) ligand in the reaction of the bis-cyclometalated Ru–Al precursor, [Ru(C_6_H_4_PPh_2_)_2_(Ph_2_PC_6_H_4_AlMe(THF))H] (**II**),^17^ with CO. A combination of experimental and computational studies has been employed to probe the structure of the resulting AlMePhos complex, [Ru(AlMePhos)(CO)_3_] (**1**), as well as its reactivity; photochemical addition of H_2_ at the Ru–Al bond and susceptibility to Lewis acid-mediated AlMe group abstraction to afford the cationic [P–Al–P]^+^ complex, [Ru(AlPhos)(CO)_3_][MeB(C_6_F_5_)_3_] (**4**).

## Experimental Section

### General Comments

All manipulations were carried out at room temperature under argon using standard Schlenk, high vacuum, and glovebox techniques using dry and degassed solvents. C_6_D_6_ was vacuum-transferred from potassium. The NMR spectra were recorded in C_6_D_6_ at 298 K on Bruker Avance 400 and 500 MHz NMR spectrometers and referenced as follows: ^1^H, δ 7.15; ^13^C, δ 128.0. The X{^1^H} spectra were referenced externally to 85% H_3_PO_4_ (X = ^31^P), CFCl_3_ (X = ^19^F), and BF_3_·OEt_2_ (X = ^11^B) at δ = 0. Coupling constants are defined using ^x^*J*_AB_ nomenclature in cases where there is absolute certainty in assignments (NB vt = virtual triplet). The IR spectra were recorded on Nicolet Nexus and Bruker ALPHA ATR-IR spectrometers. In situ ReactIR monitoring of the conversion of **1** to **4** was carried out with a Mettler Toledo REACTIR15 system. Elemental analyses were performed by Elemental Microanalysis Ltd., Okehampton, Devon, U.K. [Ru(C_6_H_4_PPh_2_)_2_{PPh_2_C_6_H_4_AlMe(THF)}H] was prepared according to the literature.^17^ B(C_6_F_5_)_3_ (Alfa Aesar) was used as received.

### [Ru(AlMePhos)(CO)_3_] (**1**)

A C_6_H_6_ (6 mL) solution of [Ru(C_6_H_4_ PPh_2_)_2_{PPh_2_C_6_H_4_AlMe(THF)}H] (200 mg, 0.17 mmol) in a J. Youngs resealable ampule was freeze–pump–thaw-degassed (three cycles), placed under 1 atm CO (or ^13^CO to afford **1-^13^CO**), and heated at 60 °C for 2 h. The resulting yellow solution was filtered by cannula, concentrated (ca. 2 mL), and precipitated by addition of pentane to leave an off-white solid, which was recrystallized from benzene/hexane. Yield: 85 mg (67%). ^1^H NMR (500 MHz, C_6_D_6_): δ 8.47 (d, ^3^*J*_HH_ = 7.0 Hz, 2H, Ar), 7.77 (m, 4H, Ar), 7.36–7.30 (m, 6H, Ar), 7.20 (m, 2H, Ar), 7.04–6.97 (m, 8H, Ar), 6.90–6.85 (m, 6H, Ar), −0.24 (s, 3H, Al*Me*). ^31^P{^1^H} NMR (202 MHz, C_6_D_6_): δ 55.2 (s; **1-^13^CO**: m). ^13^C{^1^H} NMR (126 MHz, C_6_D_6_): δ 204.4 (t, ^2^*J*_CP_ = 17 Hz, Ru-*C*O; **1**-**^13^CO**: dt, ^2^*J*_CC_ = 27 Hz, ^2^*J*_CP_ = 17 Hz), 202.0 (t, ^2^*J*_CP_ = 13 Hz, Ru-*C*O; **1-^13^CO**: dt, ^2^*J*_CC_ = 27 Hz, ^2^*J*_CP_ = 13 Hz), 198.0 (t, ^2^*J*_CP_ = 6 Hz, Ru-*C*O; **1-^13^CO**: br t, ^2^*J*_CP_ = 5 Hz), 170.5 (vt, *J* = 32 Hz, *i*-*C*-PAr), 142.7 (vt, *J* = 32 Hz, *i*-*C*-PAr), 137.3 (vt, *J* = 14 Hz, PAr), 137.0 (m, PAr), 133.6 (vt, *J* = 6 Hz, PAr), 132.5 (vt, *J* = 6 Hz, PAr), 131.4 (vt, *J* = 5 Hz, PAr), 130.4 (s, PAr), 130.1 (s, PAr), 129.2 (s, PAr), 128.8 (vt, *J* = 5 Hz, PAr), 128.5 (s, PAr), 128.4 (vt, *J* = 4 Hz, PAr), 126.4 (vt, *J* = 4 Hz, PAr), −5.0 (Al*Me*: observed by ^1^H-^13^C HSQC). IR: ν_CO_ (C_6_D_6_) = 2047, 1991, 1973 cm^–1^; ν_CO_ (ATR) = 2042, 1992, 1962. Anal. found: C, 65.43; H, 4.40. Calcd. for C_40_H_31_O_3_AlP_2_Ru·0.5C_6_H_6_: C, 65.48; H, 4.34.

### [Ru(AlMePhos)(CO)_2_(μ-H)H] (**2**)

A C_6_D_6_ (0.5 mL) solution of **1** (6 mg, 0.008 mmol) was freeze–pump–thaw-degassed (three cycles) in a J. Youngs resealable NMR tube and placed under 1 atm H_2_. The tube was placed in a beaker of ice-cooled water and irradiated with a 500 W Hg arc lamp. Conversion to **2** (complete on this scale of reaction in ca. 3.5 h) was assessed by periodic removal of the sample from the lamp and NMR analysis. A larger scale reaction (20 mg of **1** in 1.5 mL of C_6_H_6_ in a J. Youngs resealable ampule) was deemed to have reached maximum conversion (based upon NMR analysis) after ca. 13 h. Selected ^1^H NMR (500 MHz, C_6_D_6_): δ 8.35 (d, ^3^*J*_HH_ = 7.3 Hz, 2H, Ar), 7.65 (m, 5H, Ar), 7.45 (m, 4H, Ar), 7.28 (m, 4H, Ar), 6.95–6.90 (m, 3H, Ar),* −0.04 (s, 3H, Al*Me*), −6.18 (td, ^2^*J*_HP_ = 20.2 Hz, ^2^*J*_HH_ = 7.4 Hz, 1H, Ru-H), −8.56 (td, ^2^*J*_HP_ = 16.4 Hz, ^2^*J*_HH_ = 7.4 Hz, 1H, Ru-H-Al). ^31^P{^1^H} NMR (202 MHz, C_6_D_6_): δ 53.3 (s). Selected ^13^C{^1^H} NMR (100 MHz, C_6_D_6_): δ 198.5 (br m, Ru-*C*O). The asterisk (*) indicates that overlap of aromatic signals with those of [Ru(PPh_3_)_2_(CO)_2_H_2_] precluded the full assignment of the aromatic ^1^H NMR signals of **2**.

### [Ru(AlPhos)(CO)_3_][MeB(C_6_F_5_)_3_] (**4**)

Complex **1** (30 mg, 0.04 mmol) and B(C_6_F_5_)_3_ (21 mg, 0.04 mmol) were added to a J. Youngs resealable NMR tube, dissolved in 0.5 mL of C_6_D_6_, and fully converted into **4** (observed by NMR spectroscopy) over 2 h at room temperature. Cannula filtration, evaporation to dryness, and redissolution in C_6_H_6_ followed by layering with pentane afforded colorless crystals of **4**. Yield: 22 mg (44%). ^1^H NMR (500 MHz, C_6_D_6_): δ 8.26 (d, ^3^*J*_HH_ = 7.0 Hz, 2H, Ar), 7.30 (t, ^3^*J*_HH_ = 7.2 Hz, 2H, Ar), 7.22 (m, 7H, Ar), 7.12 (m, 1H, Ar), 7.00–6.84 (m, 16H, Ar), 1.58 (s, 3H, *Me*B(C_6_F_5_)_3_). ^31^P{^1^H} NMR (202 MHz, C_6_D_6_): δ 52.3 (s). ^13^C{^1^H} NMR (126 MHz, C_6_D_6_): δ 197.6 (t, ^2^*J*_CP_ = 13 Hz, Ru-*C*O), 194.8 (t, ^2^*J*_CP_ = 6 Hz, Ru-*C*O), 160.2 (vt, *J* = 26 Hz, PAr), 149.5 (br m, PAr), 147.6 (br m, PAr), 143.0 (vt, *J* = 31 Hz, PAr), 137.0 (vt, *J* = 12 Hz, PAr), 134.3 (vt, *J* = 24 Hz, PAr), 133.0 (vt, *J* = 6 Hz, PAr), 132.8 (vt, *J* = 5 Hz, PAr), 132.4 (vt, *J* = 6 Hz, PAr), 132.2 (vt, *J* = 6 Hz, PAr), 131.4 (s, PAr), 131.0 (s, PAr), 129.2 (vt, *J* = 6 Hz, PAr), 13.4 (B*Me*: observed by ^1^H-^13^C HSQC). ^11^B{^1^H} NMR (160 MHz, C_6_D_6_): δ −14.7 (br s). ^19^F{^1^H} NMR (470 MHz, C_6_D_6_): δ −132.1 (br “s”, 2F), −162.0 (br “s”, 1F), −165.7 (br “s”, 2F). IR: ν_CO_ (C_6_D_6_) = 2073, 2051 cm^–1^. Anal. found: C, 55.32; H, 2.52. Calcd. for C_58_H_31_BO_3_F_15_AlP_2_Ru: C, 55.22; H, 2.48.

### X-ray Crystallography

Data for compounds **1** and **4** were collected on an Agilent SuperNova instrument using a Cu Kα source. Both experiments were conducted at 150 K, solved using SHELXT,^18,19^ and refined using SHELXL^18^ via the Olex2^20^ interface. In the structure of **1**, the asymmetric unit plays host to one and a half molecules of benzene in addition to one molecule of the bimetallic complex. Both the organometallic molecule and full-occupancy solvent entity are entirely ordered. However, the additional half benzene moiety is disordered in a 50:50 ratio between two components. One of these lies close to a 2-fold crystallographic rotation axis, and the other has two fractional occupancy carbons, which are coincident with said symmetry element. Both ADP restraints and C–C distance restraints were included for fractional occupancy carbon atoms. The perfluorophenyl group based on C53, in the structure of **4**, was treated for 57:43 disorder. The rings of both components were refined as rigid hexagons. Additionally, both C–F and B–C distances (involving these fractional occupancy atoms) were refined, subject to respective similarity restraints. The hydrogens attached to C4 were located and refined, subject to being equidistant from the parent carbon.

### Computational Studies

DFT calculations were run with Gaussian 09 (Revision D.01).^21^ Geometry optimizations and thermodynamic corrections were performed with the BP86 functional^22,23^ with Ru, Al, and P centers described by Stuttgart RECPs and associated basis sets^24^ and 6-31G** basis sets for all other atoms.^25,26^ A set of d-orbital polarization functions was added to P (ζ^d^ = 0.387).^27^ All stationary points were fully characterized via analytical frequency calculations as either minima (all positive frequencies) or transition states (one negative frequency), and the latter were characterized via IRC calculations and subsequent geometry optimizations to confirm the adjacent minima. Electronic energies were recomputed with the ωB97x-D functional^28^ using def2-TZVP basis sets^29,30^ and a correction for benzene solvent (PCM approach).^31^ This protocol was previously successful in reproducing the relative free energies of a range of Ru–Zn heterobimetallic complexes in solution.^32^ Details of all computed structures are provided in the Supporting Information. Quantum theory of atoms in molecules (QTAIM) analyses^33^ were performed with AIMALL^34^ and used the extended wavefunction format. Extended transition state-energy decomposition analysis (ETS-EDA) calculations were run with the Amsterdam Modeling Suite (AMS) 2020.102.^35^

## Results and Discussion

### Synthesis of [Ru(AlMePhos)(CO)_3_] (**1**) and Reactivity with H_2_

**Figure 1:**
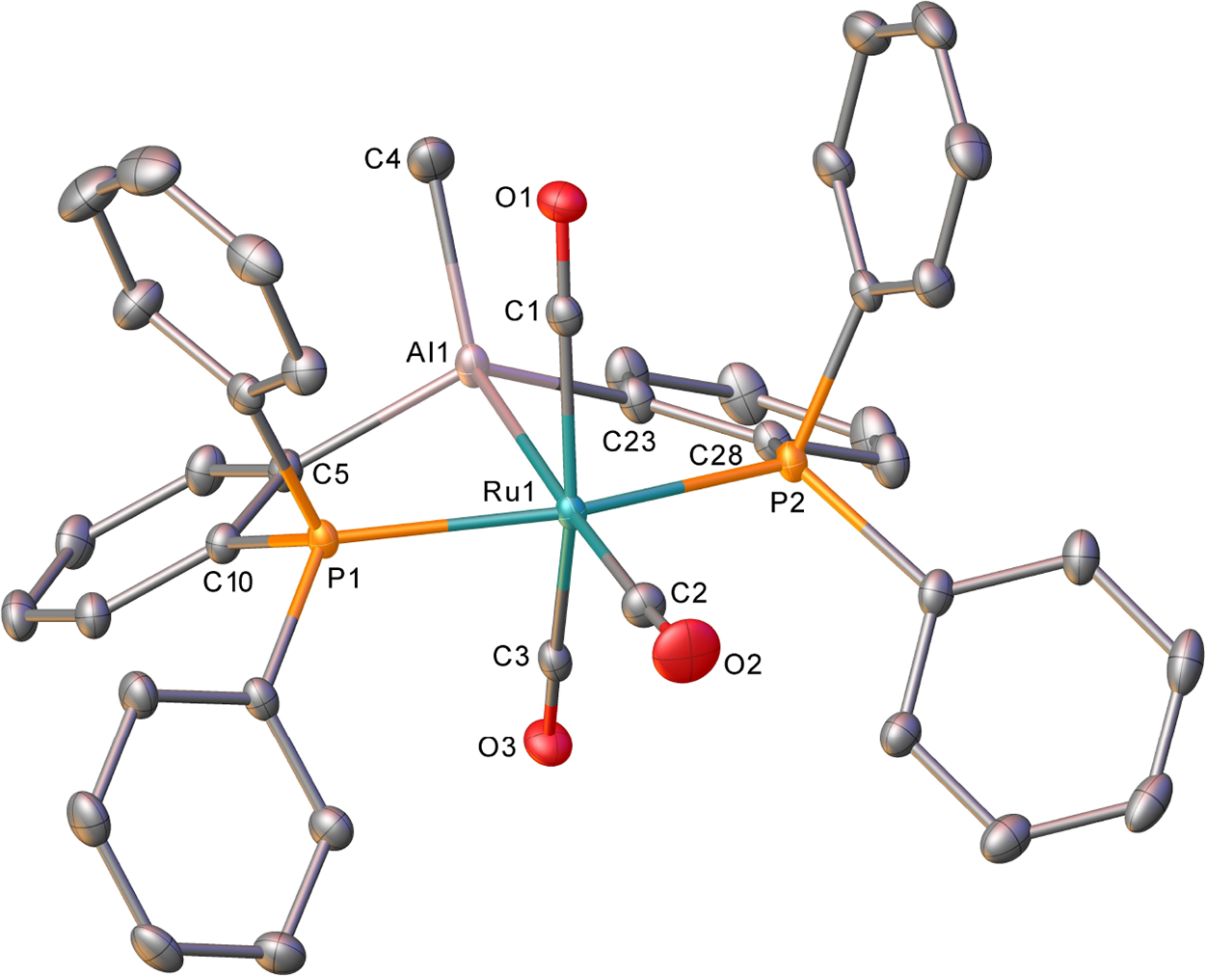
Molecular structure of **1**. Ellipsoids are represented at 30% probability. Hydrogen atoms and solvent have been omitted for clarity.

Heating a benzene solution of [Ru(C_6_H_4_PPh_2_)_2_(Ph_2_PC_6_H_4_AlMe(THF))H]^17^ under 1 atm CO for 2 h at 60 °C brought about clean conversion to [Ru(AlMePhos)(CO)_3_] (**1**, Scheme 2), which was isolated as an off-white solid in 67% yield and fully characterized using a combination of NMR and IR spectroscopy (Figures S1–S7), X-ray crystallography (Figure 1), and elemental analysis. The *C*_s_ symmetry imposed by the Al–Me group resulted in the appearance of three signals associated with the carbonyl groups in both the ^13^C{^1^H} NMR spectrum (δ 204, 202, and 198) and IR spectrum (2047, 1991, and 1973 cm^–1^). The IR stretches are ca. 30–40 cm^–1^ higher in frequency than those in [Ru(ZnPhos)(CO)_3_], indicative of the Ru center being less electron-rich on account of the stronger *Z*-acceptor properties of the AlMePhos ligand. This was also borne out structurally, as evidenced by the lengthening of the Ru–*C*O distance trans to E (E = AlMe, 1.971(2) Å; E = Zn, 1.951(3) Å). The Ru–Al distance of 2.6578(6) Å is within the sum of the covalent radii (2.67 Å),^36^ indicative of a direct Ru–Al bond, and this is supported by the presence of a Ru–Al bond path in a QTAIM study (Figure S26). A more detailed discussion of the structure of **1** is provided below when comparing with the Me-abstracted [AlPhos]^+^ complex **4**.

No thermal reaction of **1** in C_6_D_6_ with dihydrogen was observed (up to 60 °C), whereas UV photolysis under H_2_ led to loss of the ^31^P NMR resonance of the starting material at δ 55.2 and formation of a new singlet at δ 53.3, which was assigned to **2** (Scheme 2), the product of CO loss and subsequent H_2_ addition. The ^1^H NMR spectrum of **2** showed triplet of doublet hydride resonances at δ −6.18 and −8.56; these were simplified to doublets with the same mutual *J*_HH_ splitting (7.4 Hz) upon ^31^P-decoupling (Figure S9), a measurement that revealed the slightly different linewidths (FWHM of 10.9 and 13.6 Hz, respectively) of the two resonances (vide infra). We were unable to isolate **2** due to the co-formation of a second product, [Ru(PPh_3_)_2_(CO)_2_H_2_] (Figures S9 and S11–13),^37,38^ which was also observed to form along with the ZnPhos photolysis product **3** (Scheme 2) and postulated to result from the cleavage of the E–C_6_H_4_ (E = Zn and Al) bonds by adventitious moisture.^15^ In support of this proposal, the concentration of the by-product varied between different experiments, showed no correlation with irradiation time (ruling out formation involving a secondary reaction with H_2_),^39^ and was (qualitatively) formed in greater amounts alongside **2** rather than **3**, which we attribute to the more polar/reactive Al–C_6_H_4_ bond.

**Figure 2:**
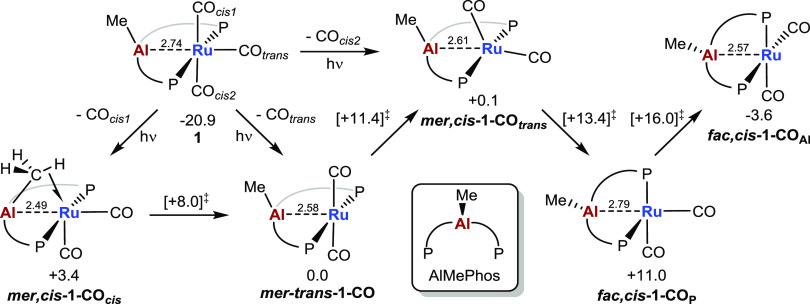
Computed isomers of [Ru(AlMePhos)(CO)_2_] (**1-CO**) with free energies in kcal/mol. Isomerization transition state energies are shown in square brackets and Ru–Al distances in Å.

Density functional theory (DFT) calculations were used to investigate both the structure and mechanism of formation of **2**. We assume that under photolytic conditions, loss of one CO ligand occurs to give 16e [Ru(AlMePhos)(CO)_2_] (**1-CO**) for which several isomers are possible (Figure 2). CO loss trans to Al gives ***mer*,*trans*-1-CO**, the free energy of which is set to 0.0 kcal/mol. Loss of the cis CO ligands leads to either ***mer*,*cis*-1-CO_*cis*_** (+3.4 kcal/mol) or ***mer*,*cis*-1-CO_*trans*_** (+0.1 kcal/mol) depending on whether the Al–Me group is *syn* or *anti* to the vacant site. All three isomers show a distinct shortening of the Ru–Al distance (**1**: 2.74 Å (2.6578(6) Å experimentally); ***mer*,*trans*-1-CO**: 2.58 Å; ***mer*,*cis*-1-CO_*trans*_**: 2.61 Å; ***mer*,*cis*-1-CO_*cis*_**: 2.49 Å). The shorter Ru–Al distance in ***mer*,*cis*-1-CO_*cis*_** reflects a distortion of the Al–Me unit to engage in an agostic interaction involving one Me C–H bond (Ru···H = 2.28 Å; C–H = 1.12 Å). Distortion of the AlMePhos backbone is also seen in ***mer*,*cis*-1-CO_*trans*_** such that some degree of Ru···C(aryl) interaction is seen (Ru···C_aryl_ = 2.60 Å). Both this and the agostic interaction in ***mer*,*cis*-1-CO_*cis*_** were corroborated by QTAIM studies (Figure S26). All three isomers can interconvert with barriers below 12 kcal/mol. As with the ZnPhos ligand, AlMePhos can also adopt a facial binding mode to give square-pyramidal geometries with either phosphorus (***fac*,*cis-*1-CO_P_**: +11.0 kcal/mol) or Al (***fac*,*cis-*1-CO_Al_**: −3.6 kcal/mol) in the axial position. ***fac*,*cis-*1-CO_Al_** is therefore the most stable isomer of **1-CO**; however, the barrier for its formation via isomerization from the *mer*-isomers is 16 kcal/mol. As this is somewhat higher than the barriers for H_2_ activation at the *mer*-isomers (vide infra), only the reactions of the latter with H_2_ were considered. The greater stability of the *fac*,*cis*-isomer in the AlMePhos system reflects the ability of the {R_2_AlMe} moiety to accommodate a pyramidal geometry at Al (Σ_angles_ at Al = 338.7°), whereas the {R_2_Zn} moiety in the equivalent isomer of [Ru(ZnPhos)(CO)_2_] showed a distorted Zn center, with a C–Zn–C angle of 150.8°.^15^

**Figure 3:**
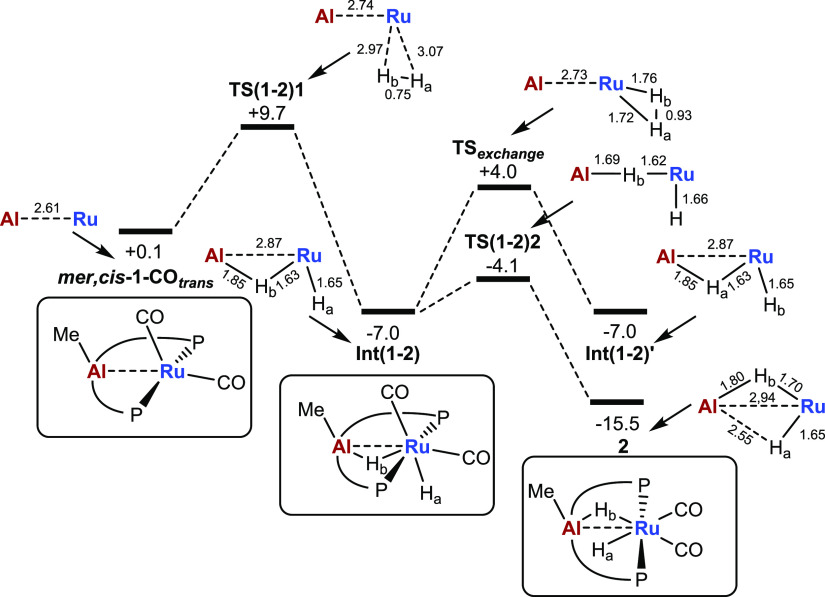
Computed reaction profile (kcal/mol) for the addition of H_2_ to ***mer*,*cis-*1-CO_*trans*_** with key distances within the {AlRuH_2_} moiety indicated in Å.

The addition of H_2_ was modeled for all three *mer*-isomers of **1-CO** and the lowest energy pathway shown to start from ***mer*,*cis*-1-CO_*trans*_** (Figure 3). H_2_ addition proceeds with a barrier of 9.6 kcal/mol via **TS(1–2)1**, which exhibits a very early transition state geometry with long Ru···H distances (2.97/3.07 Å) and minimal H–H bond elongation (0.75 Å). The distinct barrier arises from the need to distort the AlMePhos backbone to remove the short Ru···C_aryl_ contact noted in the structure of ***mer*,*cis*-1-CO_*trans*_** to make the vacant site at Ru available for H_2_ addition. Beyond this transition state, H_2_ cleavage proceeds without any subsequent barrier to give **Int(1–2)** at −7.0 kcal/mol. This intermediate exhibits one terminal Ru–hydride (Ru-H_a_ = 1.65 Å) and a second hydride that bridges the Ru···Al vector (Ru-H_b_ = 1.63 Å; Al-H_b_ = 1.85 Å) with the Ru···Al distance increasing as a result to 2.87 Å. A facile rearrangement via **TS(1–2)2** at −4.1 kcal/mol moves H_b_ across the Ru···Al vector to give **2** at −15.5 kcal/mol. The geometry of **2** again suggests one terminal hydride positioned anti to the Al–*Me* group (Ru-H_a_ = 1.65 Å) and one bridging hydride syn to Al–*Me* (Ru-H_b_ = 1.70 Å; Al-H_b_ = 1.80 Å; Ru···Al = 2.94 Å), and this is supported by a QTAIM study that showed the presence of the corresponding Ru-H_a_, Ru-H_b_, and Al-H_b_ bond paths but no Al···H_a_ bond path (Figure S26). The computational findings were consistent with experimental observations on **2**: the presence of two hydride resonances, the slightly broader nature of the lower frequency signal from bridging between Ru and quadrupolar Al, and *T*_1_ values (400 MHz, 298 K) of 730 ms (δ −6.2) and 515 ms (δ −8.6), consistent with classical hydrides.^40^

Of the other *mer*-isomers, H_2_ activation at ***mer*,*cis*-1-CO_*cis*_** proceeds through a similar pathway with a slightly higher barrier of 11.5 kcal/mol for the initial H_2_ addition step. For ***mer*,*trans*-1-CO**, H_2_ addition entails a smaller barrier of 4.3 kcal/mol to form an η^2^-H_2_ complex, ***mer-trans*-Int(1–2)1**, at −2.5 kcal/mol. This species can then isomerize to **2**; however, this process has an overall barrier of 15.7 kcal/mol. H_2_ addition to ***mer*,*trans*-1-CO** will therefore be a reversible process, with isomerization to either of the *mer*,*cis*-isomers providing access to the lower energy H_2_ activation pathways associated with those species (Figures S24 and S25).

In general, the different isomers of **1-CO** and their interconversion and reactivity with H_2_ all follow a similar pattern to that reported previously for their ZnPhos analogues.^15^ However, the presence of the Al–*Me* group places the two hydrides in **2** in different environments (H_a_: terminal; H_b_: bridging) and hence offers the possibility of H_a_/H_b_ exchange. This was defined computationally from **2** by reverting back to **Int(1–2)** in which both H_a_ and H_b_ are on the same side of the Ru···Al vector (Figure 3). H_a_/H_b_ exchange then proceeds through **TS_exchange_** at +4.0 kcal/mol that corresponds to the rotation of an η^2^-H_a_-H_b_ moiety. The overall barrier for H_a_/H_b_ exchange is therefore predicted to be 19.5 kcal/mol. An alternative pathway involving inversion at the Al center was found to have a higher barrier of 33.8 kcal/mol. This low computed exchange barrier was verified experimentally by the appearance of an EXSY signal between the two hydrides, as well as a NOESY correlation from both hydrides to the Al–*Me* resonance (Figure S10).

### Complexation of [AlPhos]^+^

**Scheme 3:**
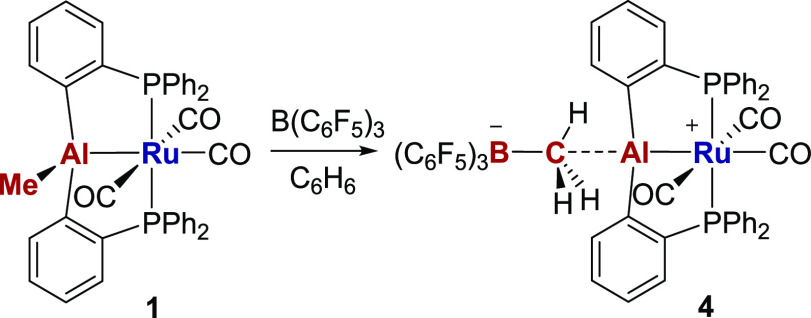
Synthesis of [Ru(AlPhos)(CO)_3_][MeB(C_6_F_5_)_3_] (**4**)

Treatment of **1** with an equimolar amount of B(C_6_F_5_)_3_ in benzene resulted in abstraction of the Al–Me group and formation of the [MeB(C_6_F_5_)_3_]^−^ salt of the cationic aluminum pincer phosphine complex, [Ru(AlPhos)(CO)_3_]^+^ (**4**) (Scheme 3). The [MeB(C_6_F_5_)_3_]^−^ anion showed a characteristic downfield shift^41^ of the methyl resonance in the ^1^H NMR spectrum from δ −0.24 in **1** to δ 1.58 in **4**. When the reaction was followed by ReactIR spectroscopy (Figure S23), loss of the carbonyl absorption bands for **1** at 2047, 1991, and 1973 cm^–1^ was accompanied by the growth of new bands for **4** at 2073 and 2051 cm^–1^, the shift to higher frequency being consistent with the presence of the more Lewis acidic [AlPhos]^+^ ligand.

**Figure 4:**
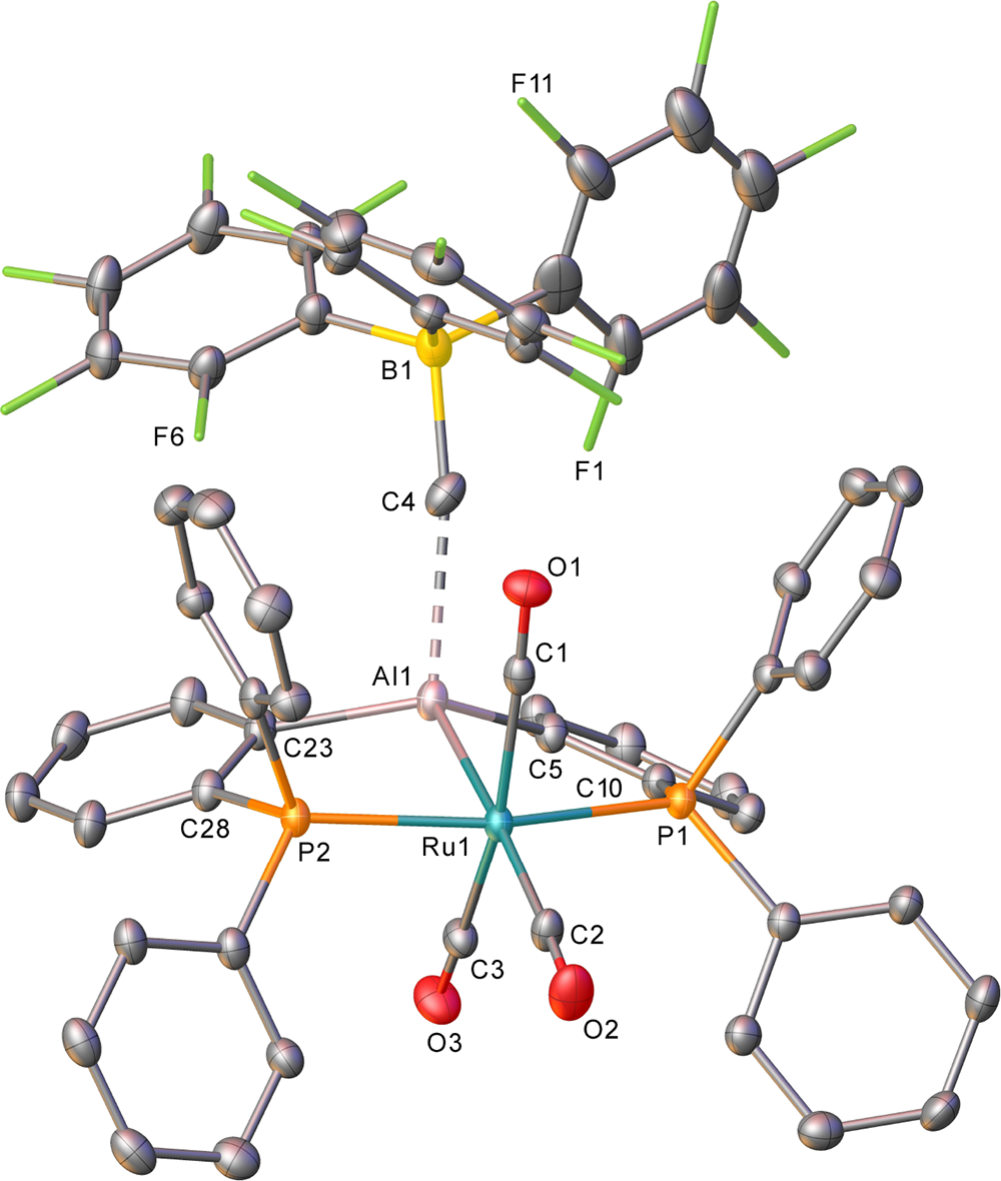
Molecular structure of **4**. Ellipsoids are represented at 30% probability. Hydrogen atoms, solvent, and the minor components of disordered atoms have been omitted for clarity.

Isolation of X-ray quality crystals yielded the structure of **4** shown in Figure 4. Particularly notable were the significant changes in the metrics relative to those in **1**: reduction of the Ru–Al distance (from 2.6578(6) to 2.5334(16) Å), elongation of the Ru–*C*O bond length trans to Al (from 1.971(2) Å to 1.986(6) Å), and shortening of the Al–*C*_6_H_4_ distances (from 2.0085 Å (average) to 1.979 Å (average)). In regard to the extent of interaction between the cation and the anion, the Al···C and B–C distances of 2.354(7) and 1.684(10) Å, respectively, and Al···C–B angle of 171.5(5)° are comparable to those found in [pySiMe_2_(TMS)AlMe][MeB(C_6_F_5_)_3_], which exhibits a crystallographically characterized Al···*Me*–B moiety.^42^ In both cases, the Al···C distance is well beyond the sum of the covalent radii (1.97 Å),^36^ although computational studies do suggest some residual interaction (see below). Near-identical diffusion coefficients (Figure S21) for the cation and anion in this species, as well as a Δ^19^F chemical shift difference of 3.7 ppm between the *meta*- and *para*-F resonances of the [MeB(C_6_F_5_)_3_]^−^ anion,^43,44^ support ion pair character in solution; this is perhaps unsurprising given the established high Lewis acidity of [AlR_2_]^+^ cations.^45−47^

Methyl group abstraction from **1** by B(C_6_F_5_)_3_ was also modeled computationally and shown to proceed from a **1**·B(C_6_F_5_)_3_ precursor adduct with a barrier of only 7.2 kcal/mol to form ion pair **4** at −6.3 kcal/mol. The Me abstraction transition state shows a near-planar CH_3_ unit (Σ_angles_ at C = 356.9°) that is equidistant between the Al and B centers (Al···C = 2.15 Å; C···B = 2.15 Å). The Ru–Al distance also shortens to 2.67 Å en route to its final computed value of 2.62 Å in **4**. As is the case for **1**, the computed Ru–Al distance in **4** is ca. 0.09 Å longer than that determined experimentally; however, the 0.12 Å shortening of the Ru–Al distance upon Me abstraction is nicely reproduced, as are the changes in Ru–*C*O distances between **1** and **4**.

Disappointingly, **4** exhibited only limited stability in solution, with redissolved crystals of the compound decomposing in C_6_D_6_ over ca. 3 days to unknown products. We postulate that this could involve reaction of the [MeB(C_6_F_5_)_3_]^−^ anion, whose non-innocence is well established.^48^

### Electronic Structure Analyses of **1** and **4** and Comparison with [Ru(ZnPhos)(CO)_3_]

The nature of the Ru–Al interactions in **1** and **4** was probed through a combination of QTAIM and ETS-EDA analyses. These were based on the experimental structures with the heavy atoms fixed from the crystal structures and the H atoms optimized with the BP86 functional. The QTAIM analysis of ion pair **4** reveals an Al···C(*Me*)–B bond path with an electron density, ρ(r), of 0.029 au at the bond critical point (BCP, Figure S26). Moreover, optimization of **4^+^** (i.e., the cation in the absence of the [MeB(C_6_F_5_)_3_]^−^ anion) resulted in a shortening of the Ru–Al distance from 2.62 to 2.51 Å and a widening of the C_aryl_–Al–C_aryl_ angle from 127° to 138°. The presence of the anion therefore has some impact on the structure of **4^+^**, implying that some degree of Al···C(*Me*) interaction is present.

BCP metrics for the Ru···Al bond paths in **1** and **4** are shown in Table 1 along with the equivalent data for the Ru···Zn bond path in [Ru(ZnPhos)(CO)_3_].^15^ All three species show low BCP ρ(r) values that are typical of TM–E bonds of this type, while the small, negative total energy densities, *H*(r), suggest a degree of covalent character.^49,50^ The small ellipticities of the Ru···Al bond paths are also indicative of cylindrical σ-interactions in both **1** and **4** despite the availability of a second vacant orbital in the latter (see the ETS-EDA analysis below). Overall, all the BCP metrics indicate that the Ru···Al interaction in **4** is somewhat stronger than in **1.** Comparison of the Ru···Al interaction in **1** with the Ru···Zn interaction in [Ru(ZnPhos)(CO)_3_] is less clear-cut, as the main indicators of the strength of interaction, ρ(r) and H(r), are contradictory (the former being smaller and the latter larger in **1**).

**Table 1:** Selected BCP Metrics (in Atomic Units) for the Ru···Al Bond Paths in **1** and **4** and the Ru···Zn Bond Path in [Ru(ZnPhos)(CO)_3_]

species	bond path	ρ(r)	∇^2^ρ(r)	ε	*H*(r)
**1**	Ru···Al	0.040	+0.052	0.045	–0.017
**4**	Ru···Al	0.051	+0.061	0.029	–0.024
[Ru(ZnPhos)(CO)_3_]	Ru···Zn	0.045	+0.039	0.035	–0.014

**Figure 5:**
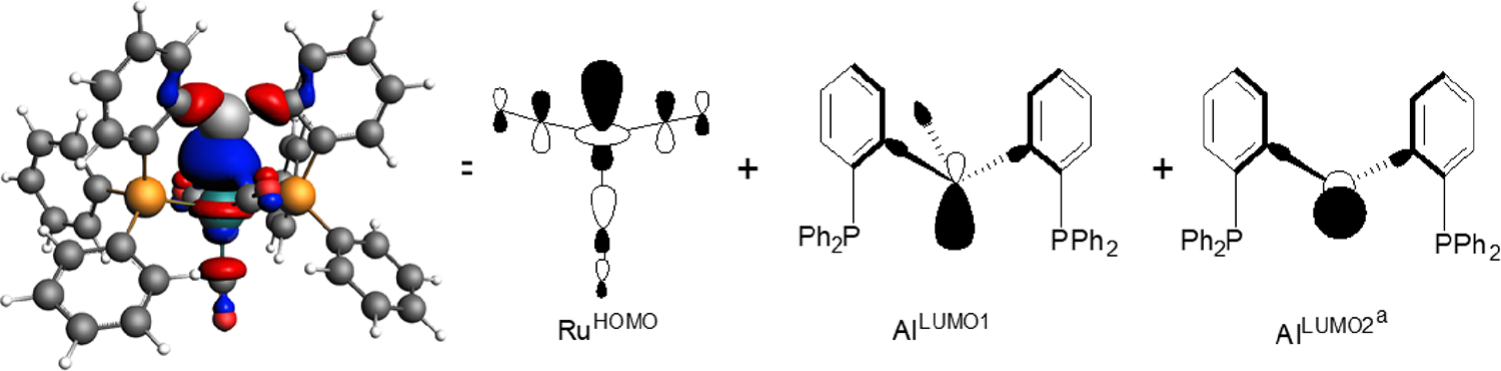
Ru–Al bonding orbital in **4^+^** (HOMO-1) and schematics of the key fragment orbitals used in the ETS-EDA calculations on **1** and **4^+^**; ^a^Al^LUMO2^ is only present in **4^+^**.

The ETS-EDA analysis was performed on **1** and the **4^+^** cation, and inspection of the molecular orbitals of this species revealed the presence of one high-lying occupied orbital with strong Ru–Al bonding character (Figure 5 for **4^+^**). The nature of this interaction was quantified within the ETS-EDA scheme by considering donation from the HOMO of the common d^8^ {Ru(CO)_3_} fragment (Ru^HOMO^, shown schematically in Figure 5) into the Al-based LUMOs on the {AlMePhos} and {AlPhos}^+^ fragments. Of these, Al^LUMO1^ is present in both fragments, whereas Al^LUMO2^ is only available in {AlPhos}^+^. A similar analysis was also performed for [Ru(ZnPhos)(CO)_3_], and the key data are collected in Table 2.

**Table 2:** ETS-EDA Data for Ru–Al Bonding in **1** and **4** and Ru–Zn Bonding in **3**^a^

	orbital populations					
species	Ru^HOMO^	Al^LUMO1^	Al^LUMO2^	Δ*Ε*^orbital^	Δ*Ε*^Total^^a^	ν_CO_ (calc)/cm^–1^
**1**	1.46	0.32		–234.2	–169.7	2013, 1964, 1953
**4**^b^	1.19	0.21	0.37^c^	–282.9	–193.2	2045, 1991, 1972
[Ru(ZnPhos)(CO)_3_]	1.51	0.12^d^		–215.7	–156.1	1982, 1942, 1922

a Δ*E*^Total^ is the computed binding energy (kcal/mol) between the {Ru(CO)_3_} fragment and the {AlMePhos}, {AlPhos}^+^, and {ZnPhos} fragments in **1**, **4^+^**, and [Ru(ZnPhos)(CO)_3_], respectively. This is the sum of Δ*E*^steric^ (not shown) and Δ*E*^orbital^, the orbital interaction:^33^ the magnitude of Δ*E*^orbital^ reflects the additional contributions from phosphine arms of the {AlMePhos} and {AlPhos}^+^ fragments.

b For the purposes of the ETS-EDA analysis, the **4^+^** cation was computed in the absence of the anion.

c Al^LUMO2^ is only present in {AlPhos}^+^.

d Occupation of the primary Zn-based acceptor orbital. Several other acceptor orbitals with Zn character are also populated to some extent but are heavily delocalized over the ZnPhos ligand, meaning that an accurate assessment of the total population at Zn is not possible.

Table 2 shows that for **1**, Ru^HOMO^ is depopulated to 1.46e, with 0.32e being donated into Al^LUMO1^. Upon Me abstraction to form **4**^+^, the population of Ru^HOMO^ decreases further to 1.19e, reflecting the availability of a second acceptor orbital and a more Lewis acidic [AlPhos]^+^ ligand, the two acceptor orbitals of which have a combined occupation of 0.58e. This is also reflected in an increase in the total interaction energy, Δ*E*^Total^, and its orbital interaction component, Δ*E*^orbital^. As the other three occupied Ru-based dπ orbitals in the {Ru(CO)_3_} fragment showed essentially no variation in occupancy between **1** and **4**^+^ (Figure S27), the stronger Ru–Al interaction in **4^+^** must arise from the stronger σ-acceptor properties of the [AlPhos]^+^ ligand rather than any π-acceptor character. This is also consistent with the low ellipticity noted in the QTAIM study. Comparing the ETS-EDA analyses of **1** and [Ru(ZnPhos)(CO)_3_] shows that the AlMePhos ligand causes a higher depopulation of Ru^HOMO^ and provides greater values of Δ*E*^Total^ and Δ*E*^orbital^. The computed trend in ligand Lewis acidity is therefore [AlPhos]^+^ > AlMePhos > ZnPhos. This is also supported by the calculated CO stretching frequencies that show an increase of 20–30 cm^–1^ from [Ru(ZnPhos)(CO)_3_] to **1** and again from **1** to **4^+^**.

## Conclusions

The synthesis and characterization of the Ru–Al heterobimetallic complex [Ru(AlMePhos)(CO)_3_] (**1**) have been presented, where AlMePhos is the novel P–Al(Me)–P pincer ligand (*o*-Ph_2_PC_6_H_4_)_2_AlMe. Under photolytic conditions, **1** loses CO and activates H_2_ to give [Ru(AlMePhos)(CO)_2_(μ-H)H] (**2**),which has been characterized by multinuclear NMR and IR spectroscopies. DFT calculations define a low energy mechanism by which H_2_ is activated at an unsaturated 16e Ru center before rearranging to form **2**, the most stable structure of which has one terminal and one bridging hydride that are respectively *anti* and *syn* to the Al*Me* group. The calculations predict facile hydride exchange on the NMR timescale, a process that was corroborated experimentally. Reaction of **1** with B(C_6_F_5_)_3_ results in Me abstraction to form the ion pair [Ru(AlPhos)(CO)_3_][MeB(C_6_F_5_)_3_] (**4**) featuring the cationic [(*o*-Ph_2_PC_6_H_4_)_2_Al]^+^ ligand, [AlPhos]^+^. Crystallographic and computational characterizations suggest that **4** exists as a close contact ion pair in the solid state with some Al···Me–B interaction; this ion pairing is retained in benzene solution. Electronic structure analyses identify a Ru–Al bond in **1** that is strengthened upon Me abstraction to form **4**. Further electronic structure analyses comparing **1** and **4** with the previously reported [Ru(ZnPhos)(CO)_3_] complex indicate that the Lewis acidity of these pincer ligands increases along the series ZnPhos < AlMePhos < [AlPhos]^+^. This is supported by the trends in both the experimental and computed ν_CO_ stretching frequencies. The AlMePhos and [AlPhos]^+^ pincer ligands add to the growing family of main group analogues^4^ of the widely used DPEPhos ligand, Ph_2_P(*o*-C_6_H_4_)_2_O.^51^

## Supporting Information Available

NMR and IR spectra of compounds **1**, **2**, and **4**; crystallographic data; computational data; computed structures and energies (PDF)
Structures and Cartesian coordinates (XYZ)

## Supplementary Material

ic2c03665_si_001.pdf

ic2c03665_si_002.xyz
