# Supplementary material for: Experimental and Computational Studies of Ruthenium Complexes Bearing Z-Acceptor Aluminum-Based Phosphine Pincer Ligands
Source: Inorg Chem. 2022 Dec 7;61(50):20690–8. doi: 10.1021/acs.inorgchem.2c03665 (PMC9768752; doi:10.1021/acs.inorgchem.2c03665)
Supplement: Supplementary file 1 — ic2c03665_si_001.pdf [file ic2c03665_si_001.pdf]

**Experimental and Computational Studies of Ruthenium Complexes Bearing *Z*-Acceptor Aluminium-Based Phosphine Pincer Ligands**

Connie J. Isaac, Cameron I. Wilson, Arron L. Burnage, Fedor M. Miloserdov, Mary F. Mahon, Stuart A. Macgregor and Michael K. Whittlesey

**Contents**

| | |
|---|---|
| NMR and IR spectra | S2 |
| Crystallographic data | S25 |
| Computational data | S26 |
| Computed Structures and Energies | S29 |

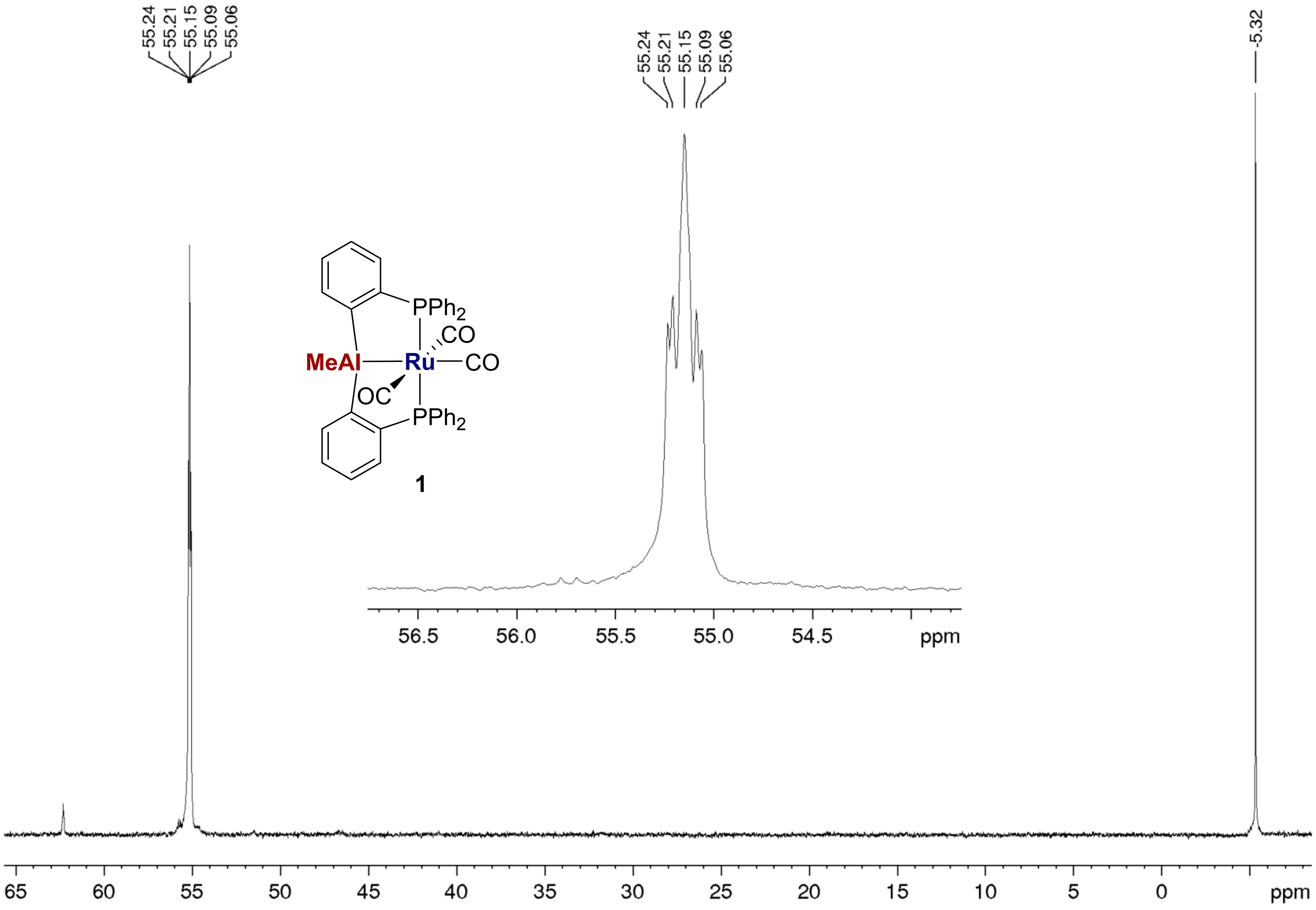


**Figure S1.** $^{31}P\{^{1}H\}$ NMR spectrum (202 MHz, $C_6D_6$, 298 K) of a solution of [Ru($C_6H_4PPh_2$)$_2$($Ph_2PC_6H_4$AlMe(THF))H] heated at 60 °C (1 h) under 1 atm $^{13}$CO illustrating elimination of $PPh_3$ and formation of [Ru(AlMePhos)($^{13}$CO)$_3$] (**1-$^{13}$CO**).

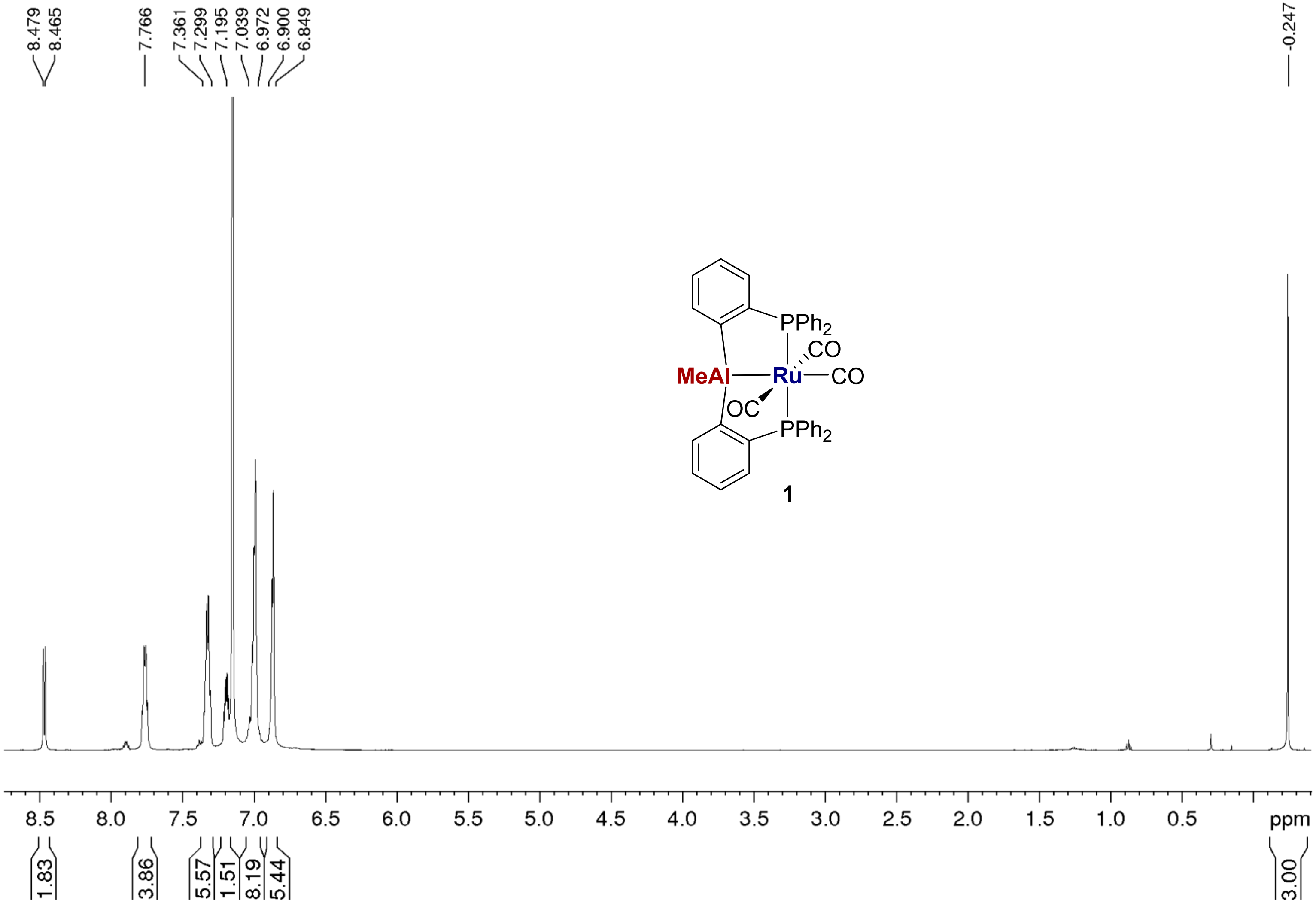


**Figure S2.** $^{1}$H NMR spectrum (500 MHz, $C_6D_6$, 298 K) of [Ru(AlMePhos)$(CO)_3$] (**1**).

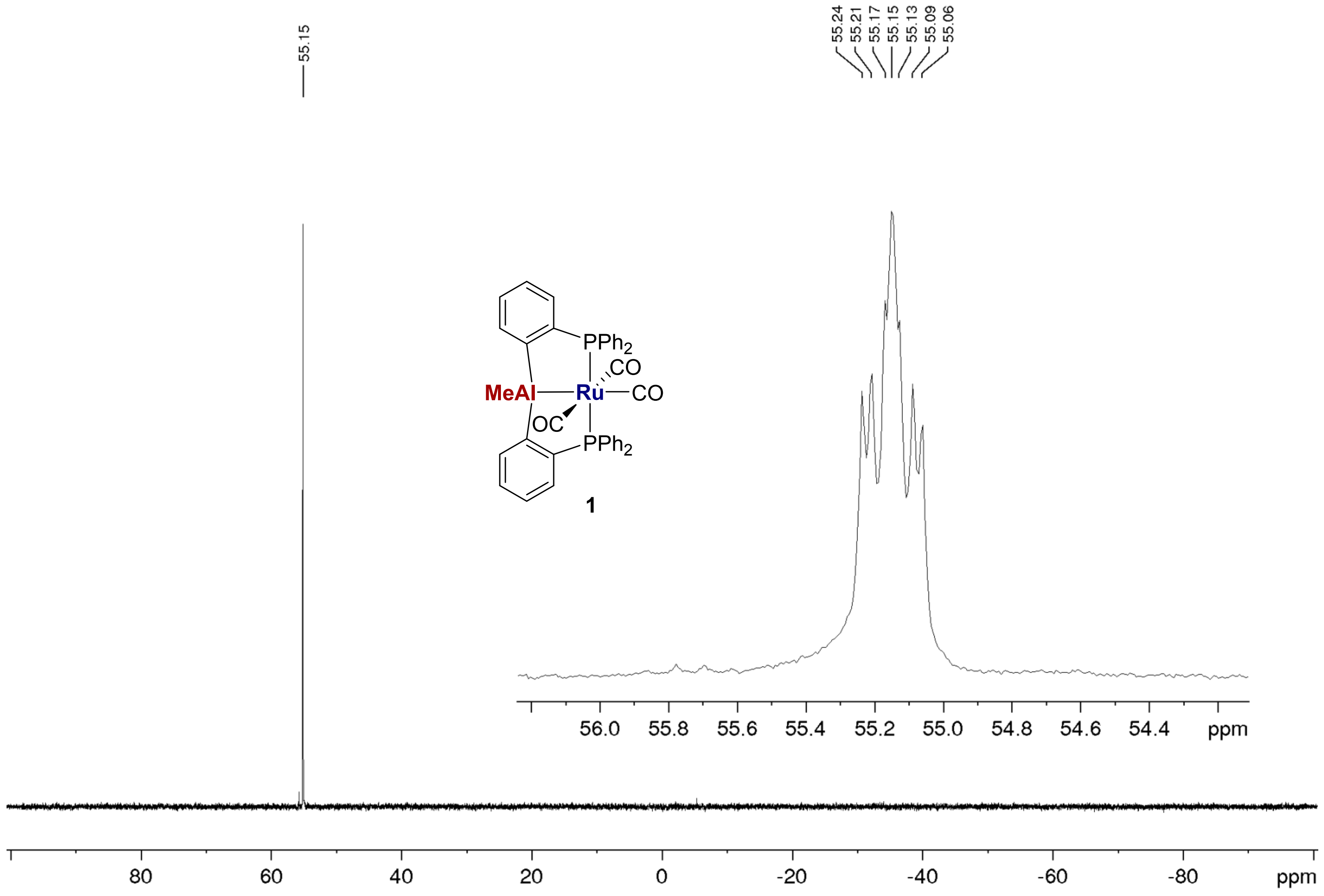


**Figure S3.** $^{31}P\{^{1}H\}$ NMR spectrum (202 MHz, $C_6D_6$, 298 K) of $[Ru(AlMePhos)(CO)_3]$ (**1**). Inset shows the signal of $^{13}CO$ labelled **1**.

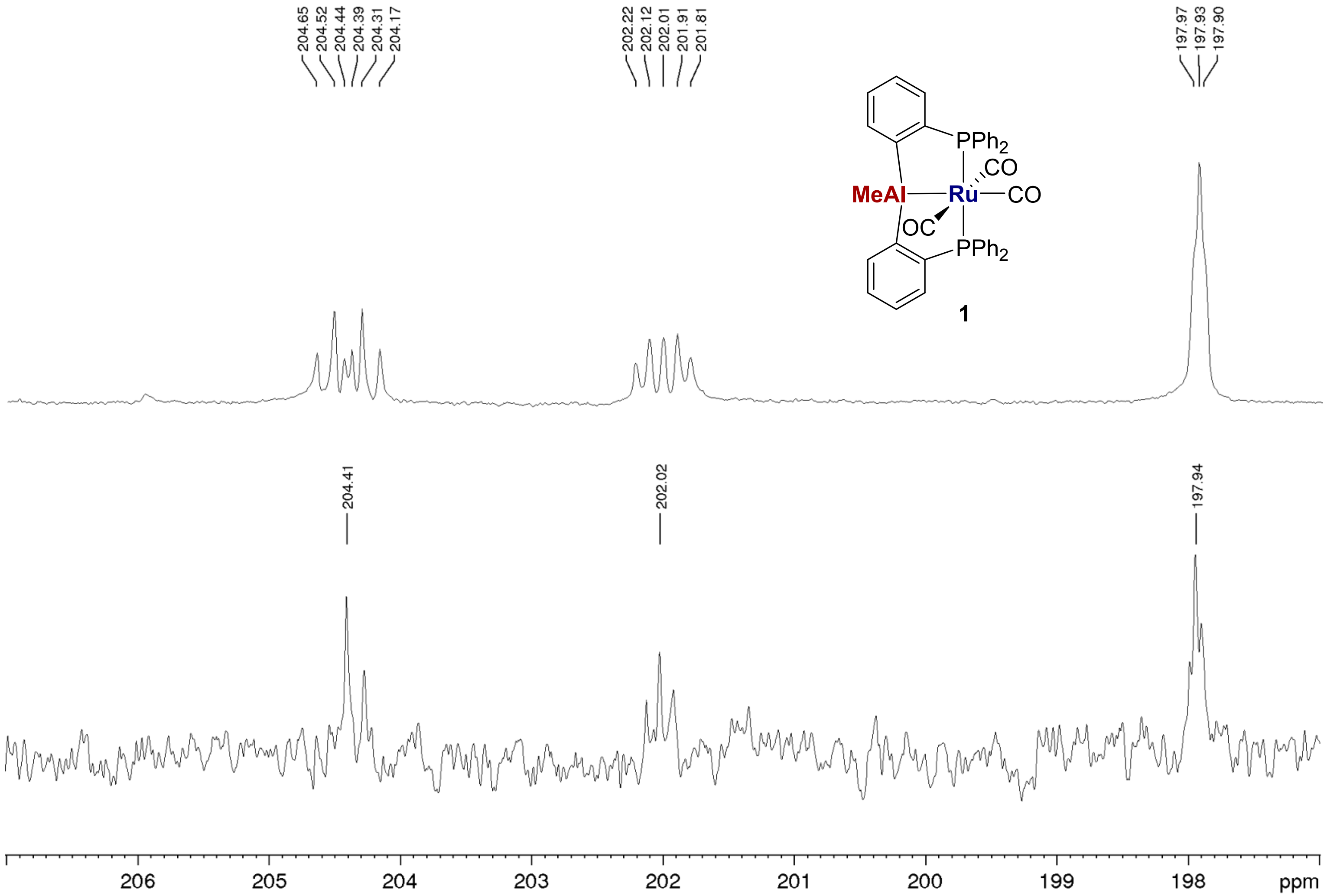


**Figure S4.** Carbonyl region of the $^{13}C\{^{1}H\}$ PENDANT NMR spectrum (125 MHz, $C_6D_6$, 298 K) of [Ru(AlMePhos)(CO)$_3$] (**1**) prepared using $^{12}CO$ (bottom) and $^{13}CO$ (top).

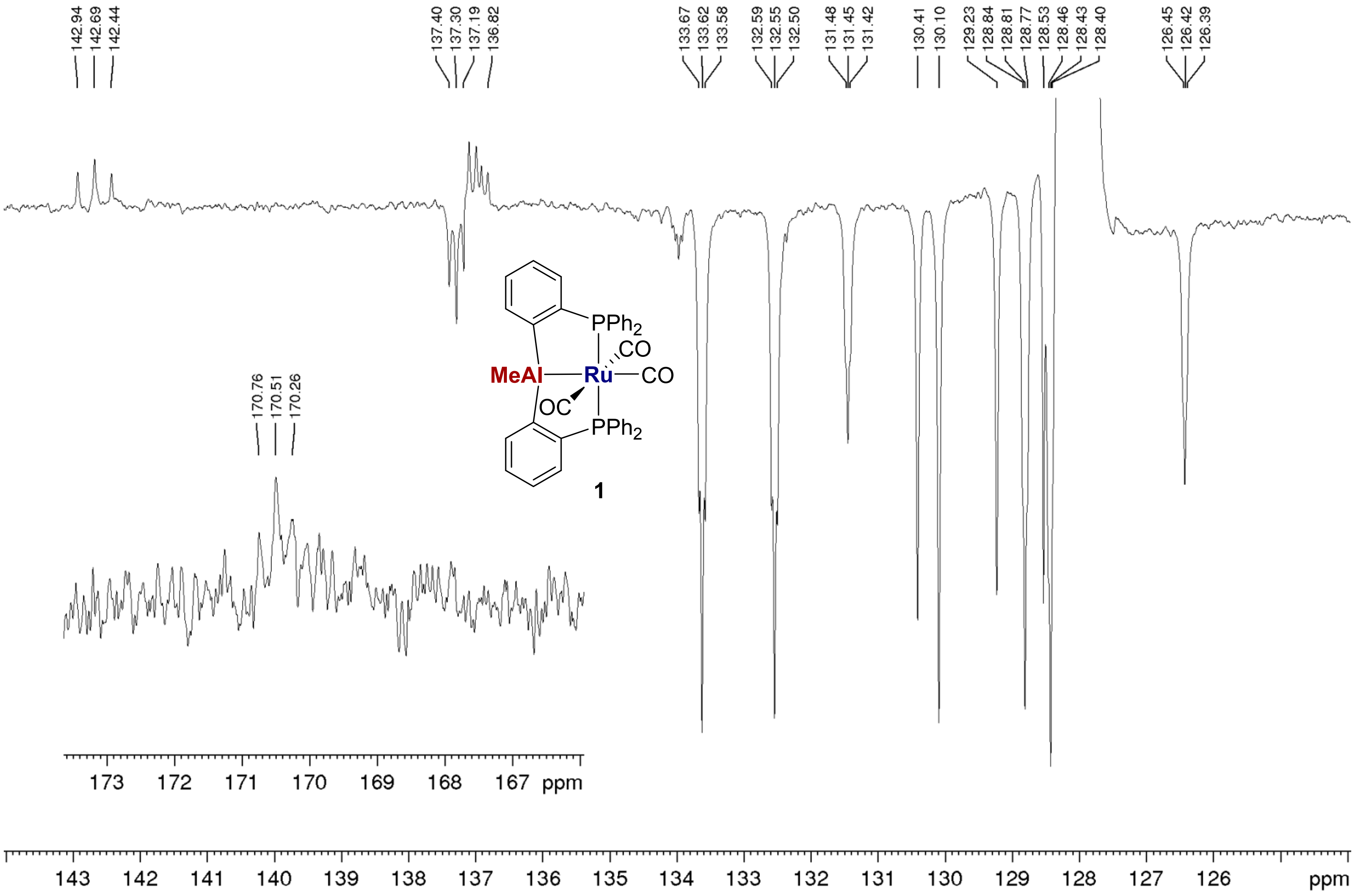


**Figure S5.** Aromatic region of the $^{13}C\{^{1}H\}$ PENDANT NMR spectrum (125 MHz, $C_6D_6$, 298 K) of [Ru(AlMePhos)(CO)$_3$] (**1**).

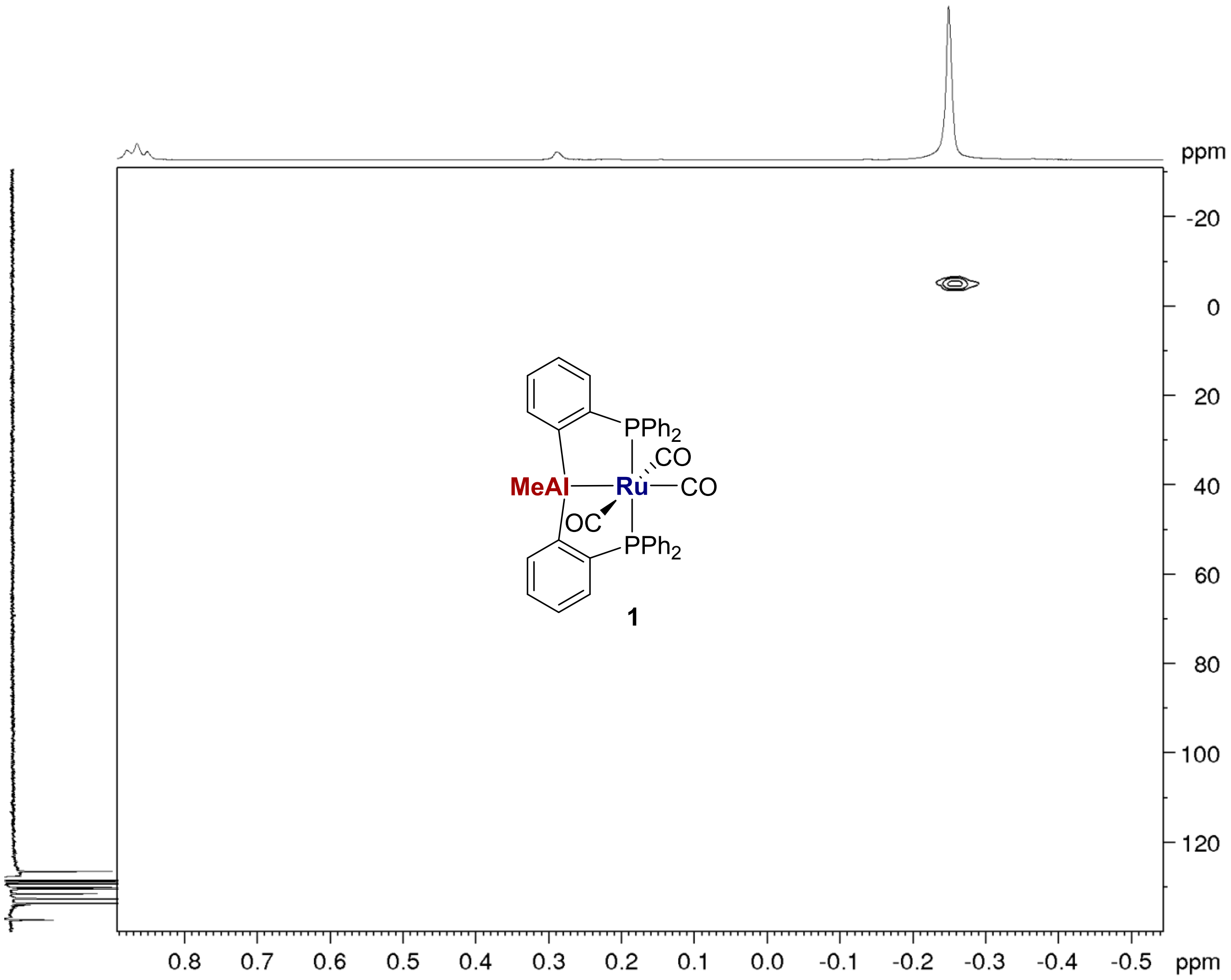


**Figure S6.** $^{1}H$-$^{13}C$ HSQC spectrum ($C_6D_6$, 298 K) showing Al-*Me* correlation signal in [Ru(AlMePhos)$(CO)_3$] (**1**).

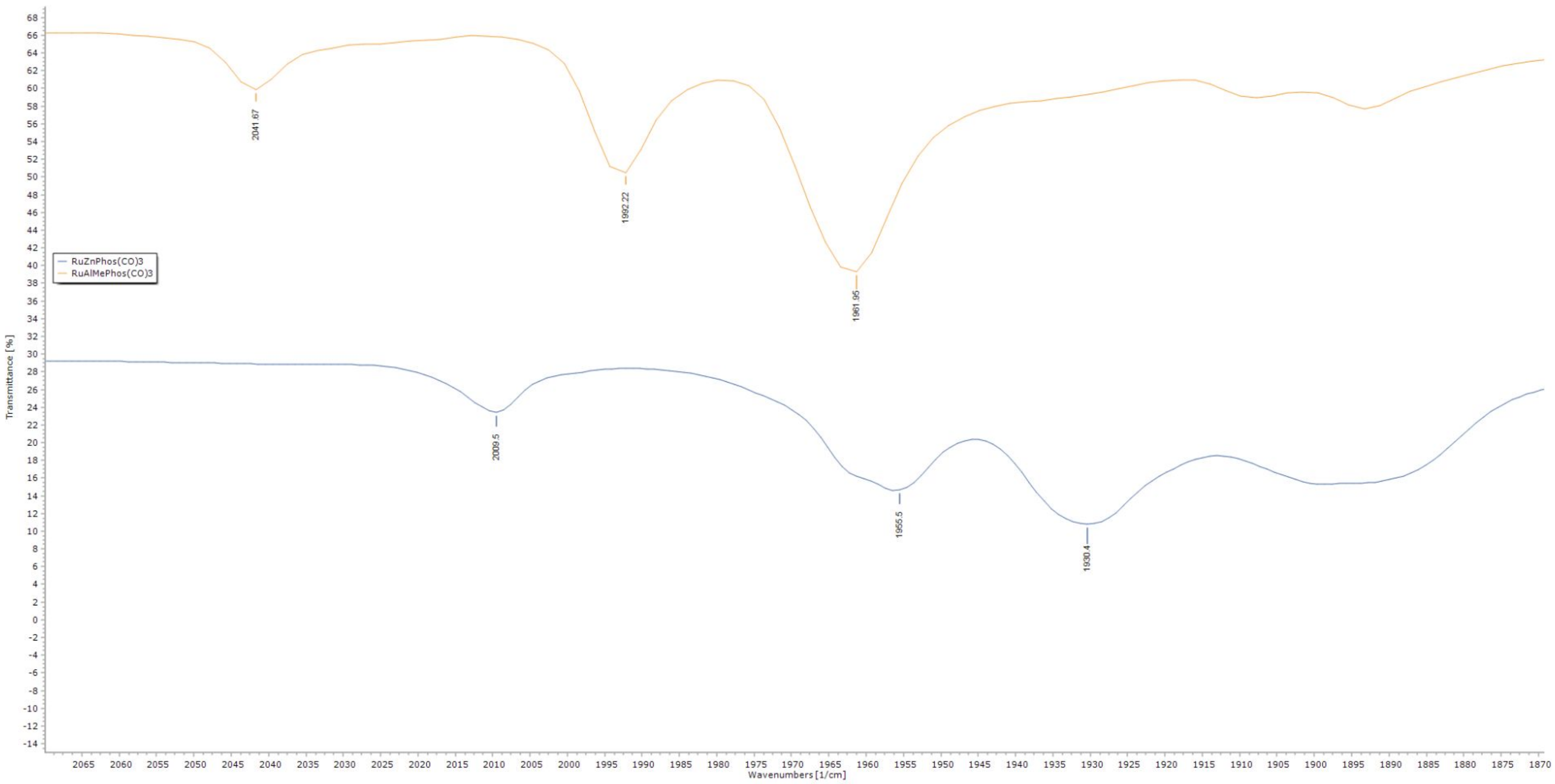


**Figure S7.** Comparison of ATR-IR spectrum of (top) [Ru(AlMePhos)$(CO)_3$] (**1**) with that of (bottom) [Ru(ZnPhos)$(CO)_3$] recorded in KBr.

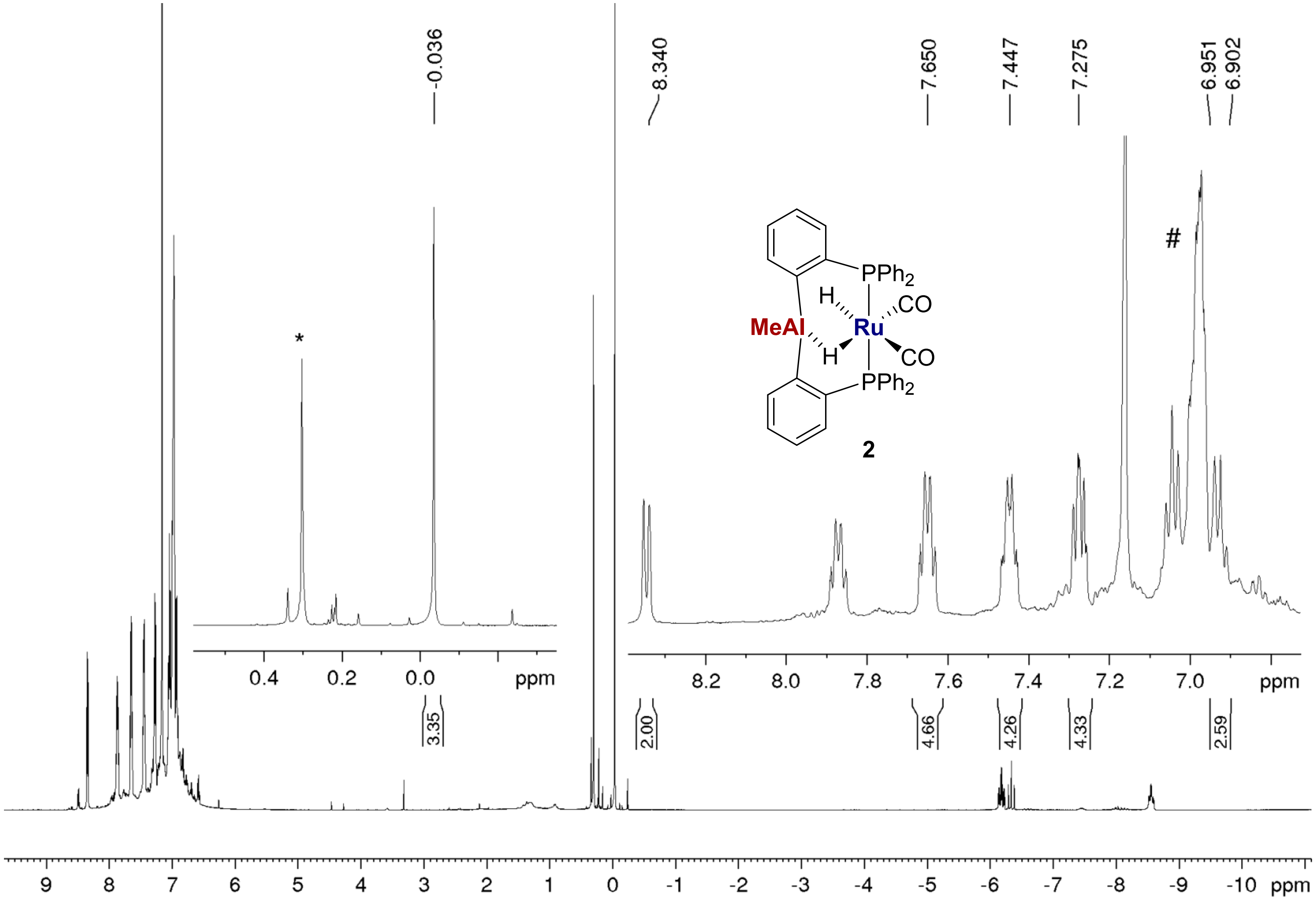


**Figure S8.** $^{1}$H NMR spectrum (500 MHz, $C_6D_6$, 298 K) of [Ru(AlMePhos)(CO)$_2$(μ-H)H] (**2**) recorded after 4 h photolysis of **1** under $H_2$ (1 atm).* denotes silicone grease. The aromatic region labelled # could not be integrated due to overlap with signals from [Ru($PPh_3$)$_2$(CO)$_2$$H_2$].

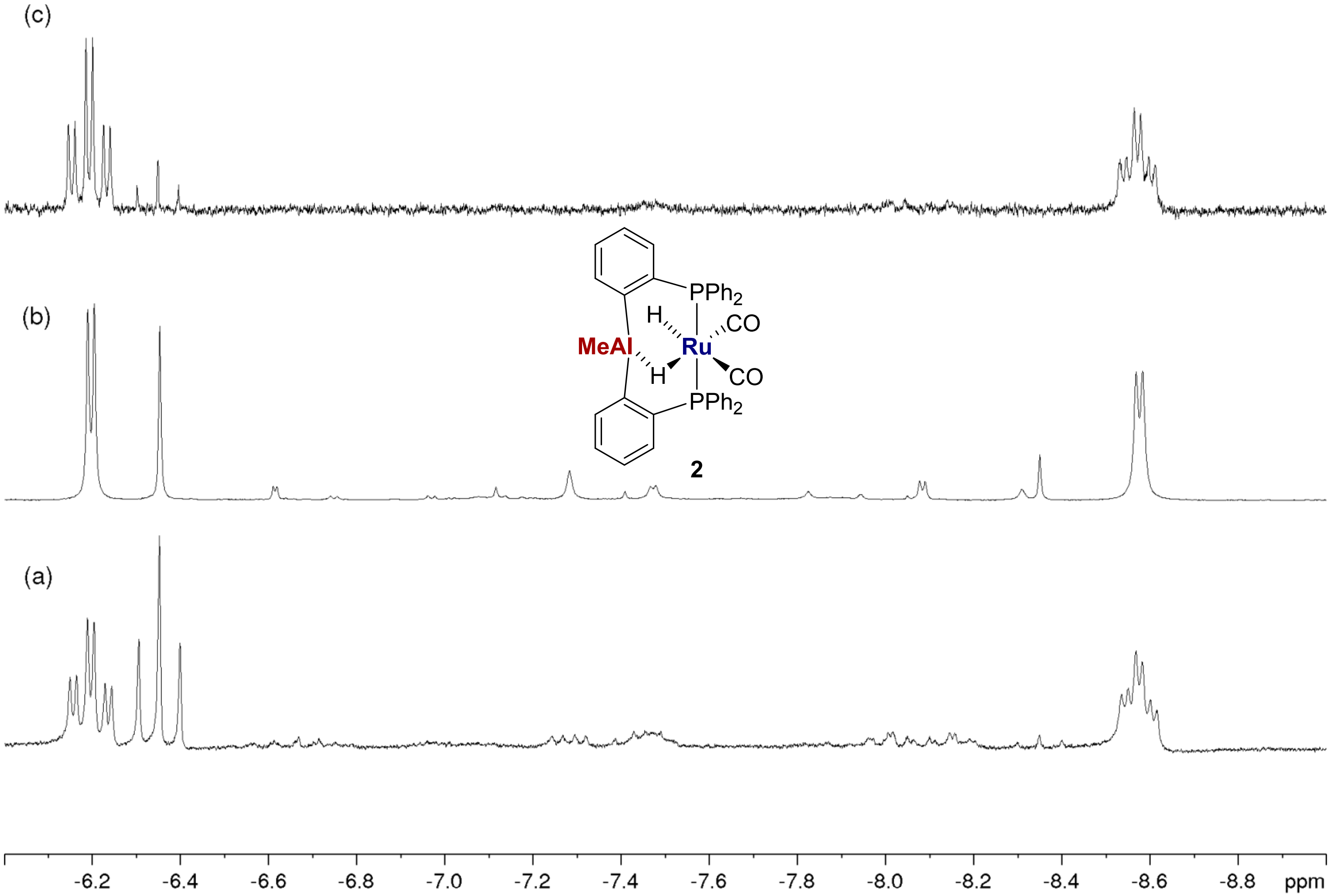


**Figure S9.** Hydride region of the (a) $^1H$ and (b) $^1H\{^{31}P\}$ NMR spectrum of **2** from Figure S8. The triplet at δ -6.3 is due to $[Ru(PPh_3)_2(CO)_2H_2]$. The variable formation of this species is highlighted by (c), a spectrum of a separate experiment after 4 h photolysis of **1** under $H_2$ in $C_6D_6$.

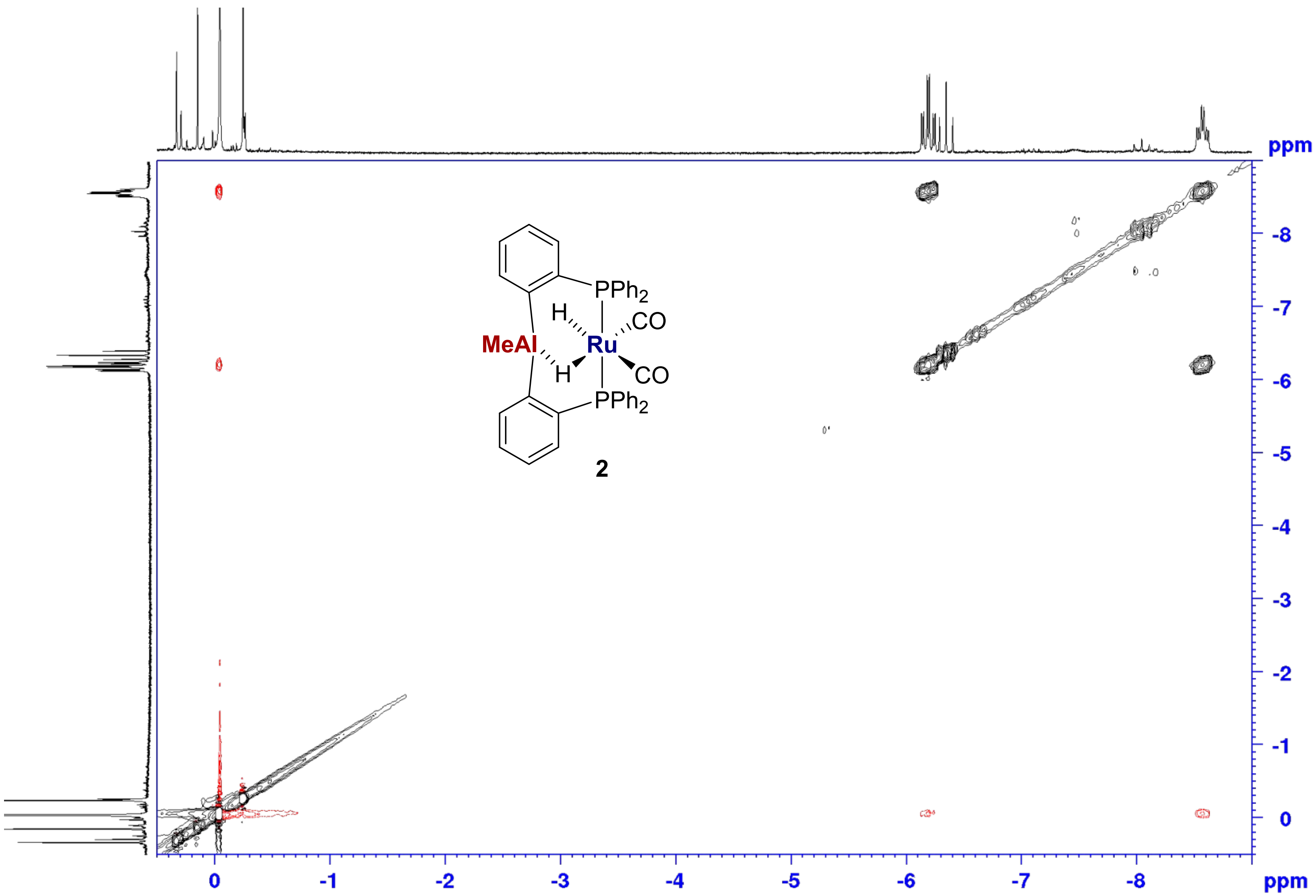


**Figure S10.** [1]H NOESY spectrum (500 MHz, $C_6D_6$, 298 K) of [Ru(AlMePhos)$(CO)_2$(μ-H)H] (**2**) illustrating EXSY signals between the hydrides and NOESY signals to the Al-*Me* resonance.

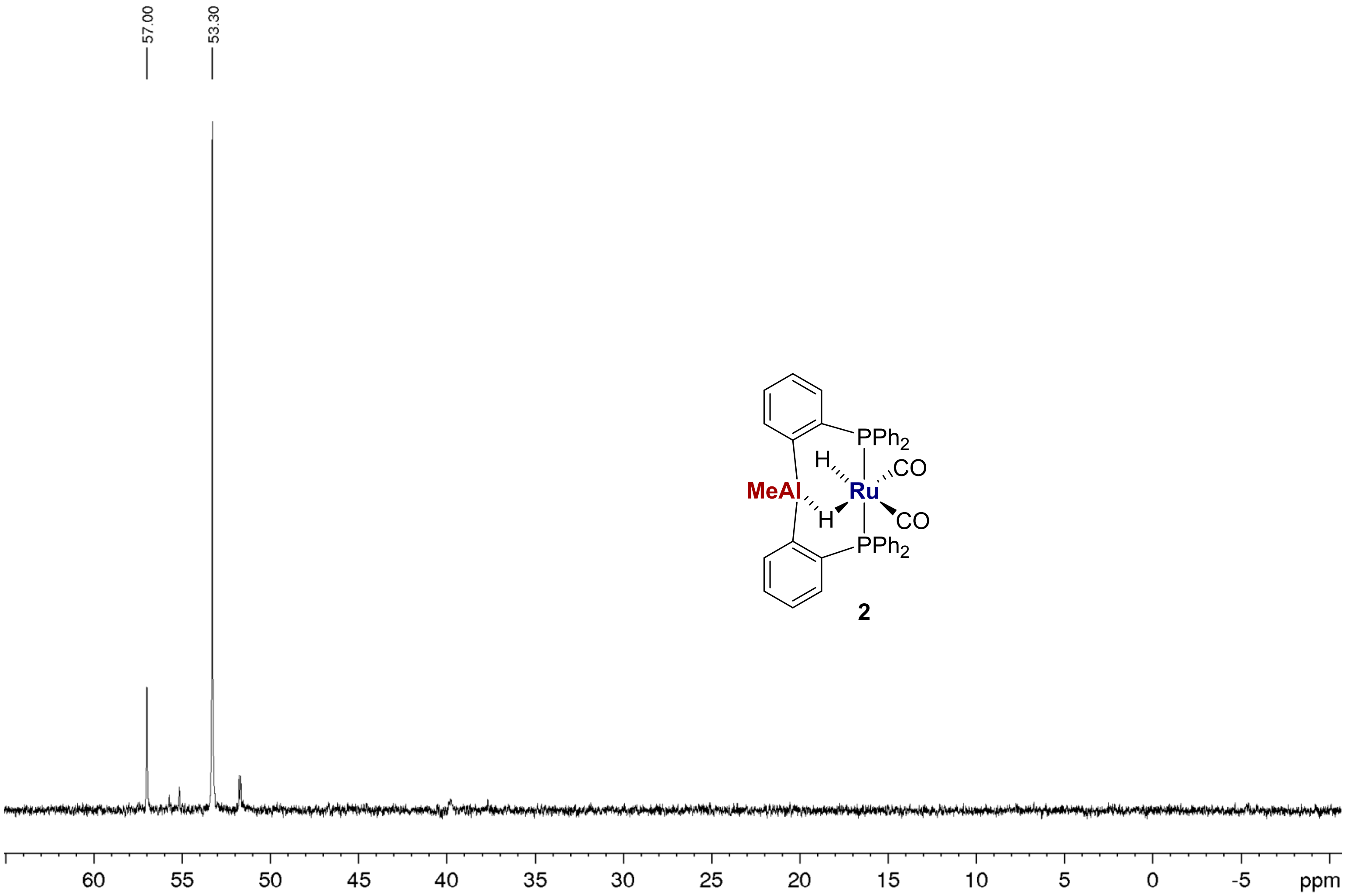


**Figure S11.** $^{31}P\{^1H\}$ NMR spectrum (202 MHz, $C_6D_6$, 298 K) of [Ru(AlMePhos)$(CO)_2$(μ-H)H] (**2**). The small singlet at ca. δ 57 is due to [Ru$(PPh_3)_2(CO)_2H_2$].

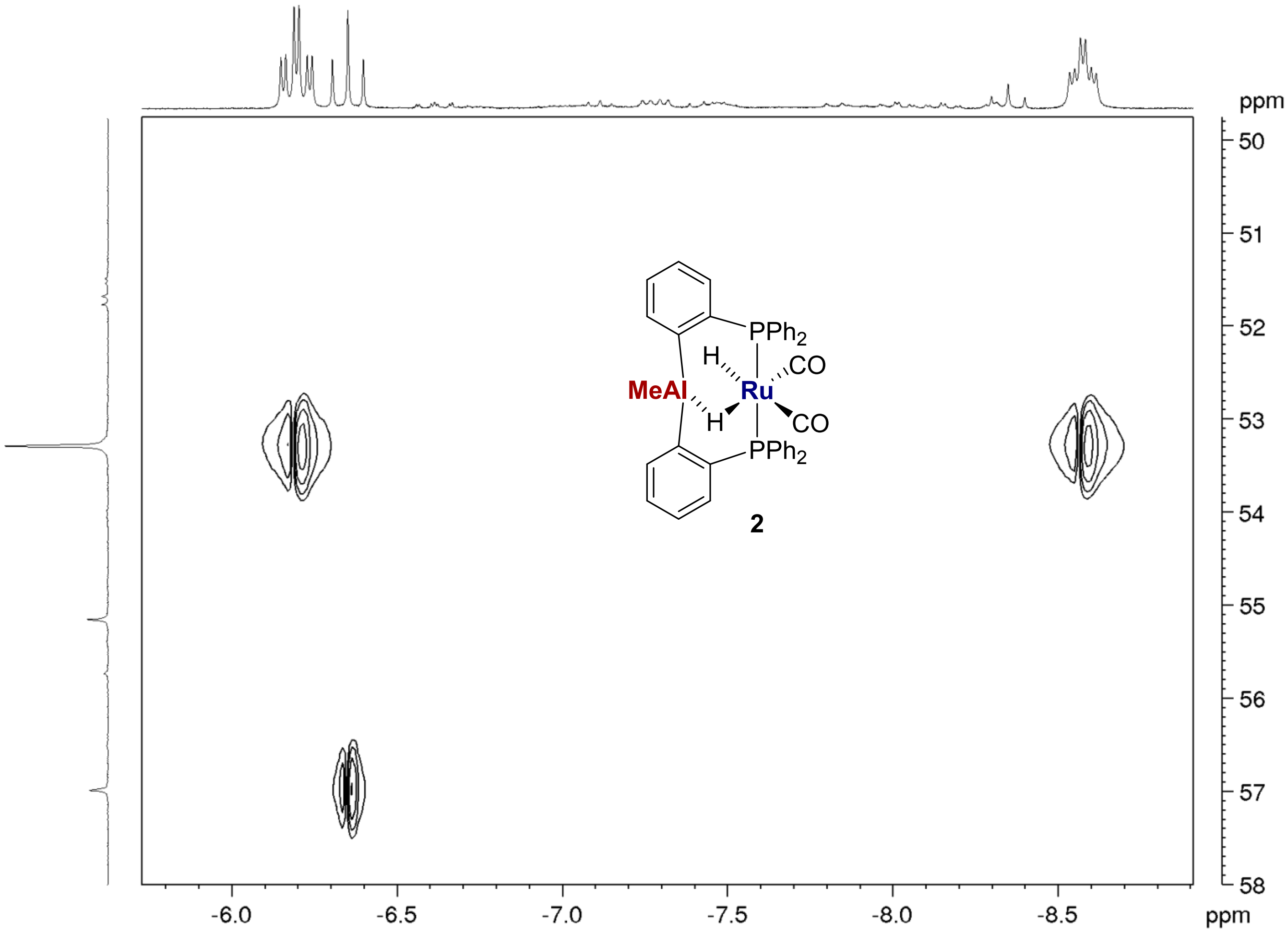


**Figure S12.** $^{1}H$-$^{31}P$ HSQC spectrum ($C_6D_6$, 298 K) of hydride correlations for [Ru(AlMePhos)$(CO)_2$(μ-H)H] (**2**) (as well as [Ru$(PPh_3)_2(CO)_2H_2$]).

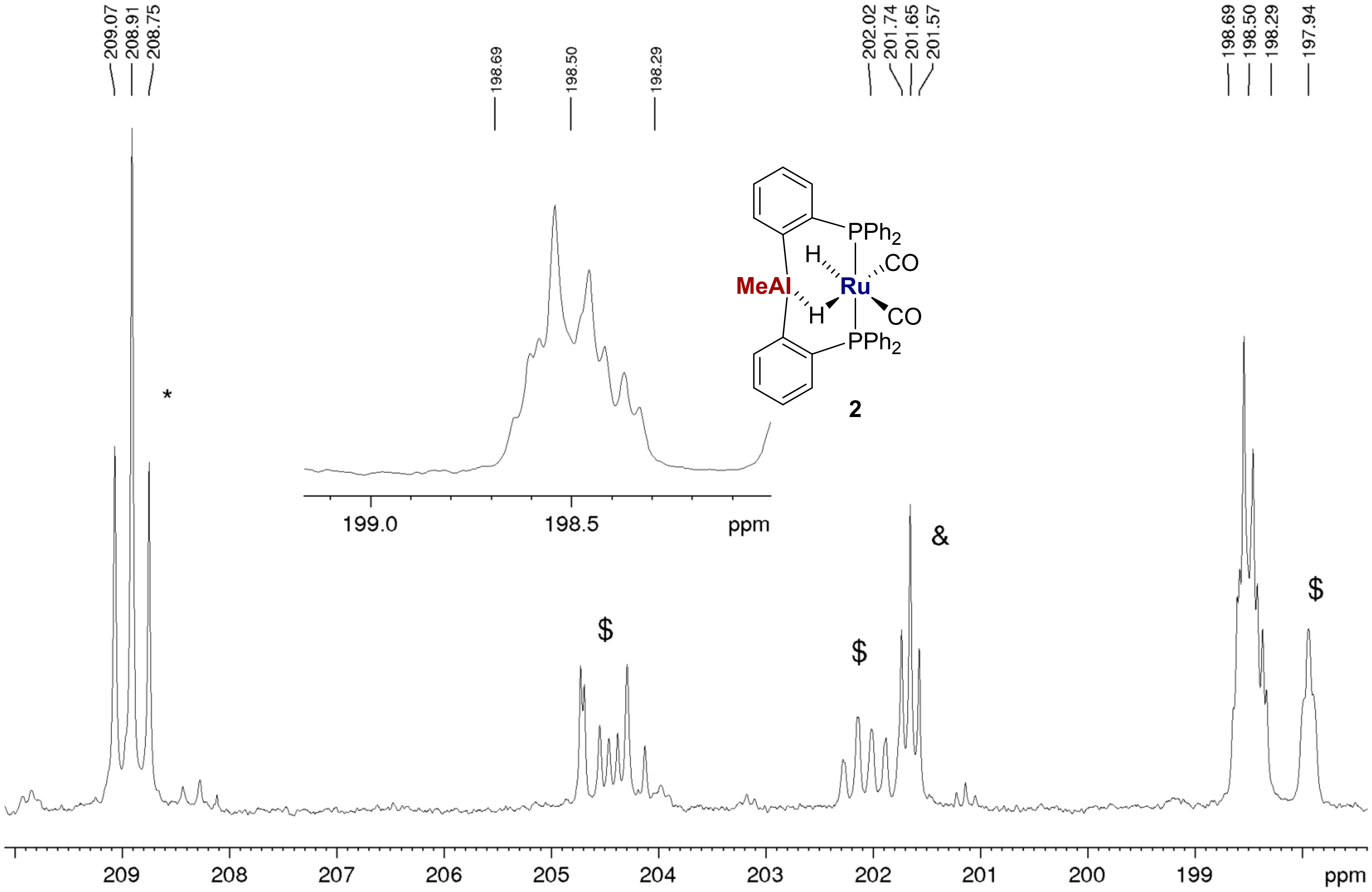


**Figure S13.** Carbonyl region of the $^{13}C\{^{1}H\}$ DEPTQ NMR spectrum (100 MHz, $C_6D_6$, 298 K) following photolysis of **1-$^{13}$CO** with $H_2$ to form [Ru(AlMePhos)($^{13}$CO)$_2$(μ-H)H] (**2-$^{13}$CO**). $ = **1-$^{13}$CO**; & = [Ru(PPh$_3$)$_2$($^{13}$CO)$_2$H$_2$]; * = [Ru(PPh$_3$)$_2$($^{13}$CO)$_3$].

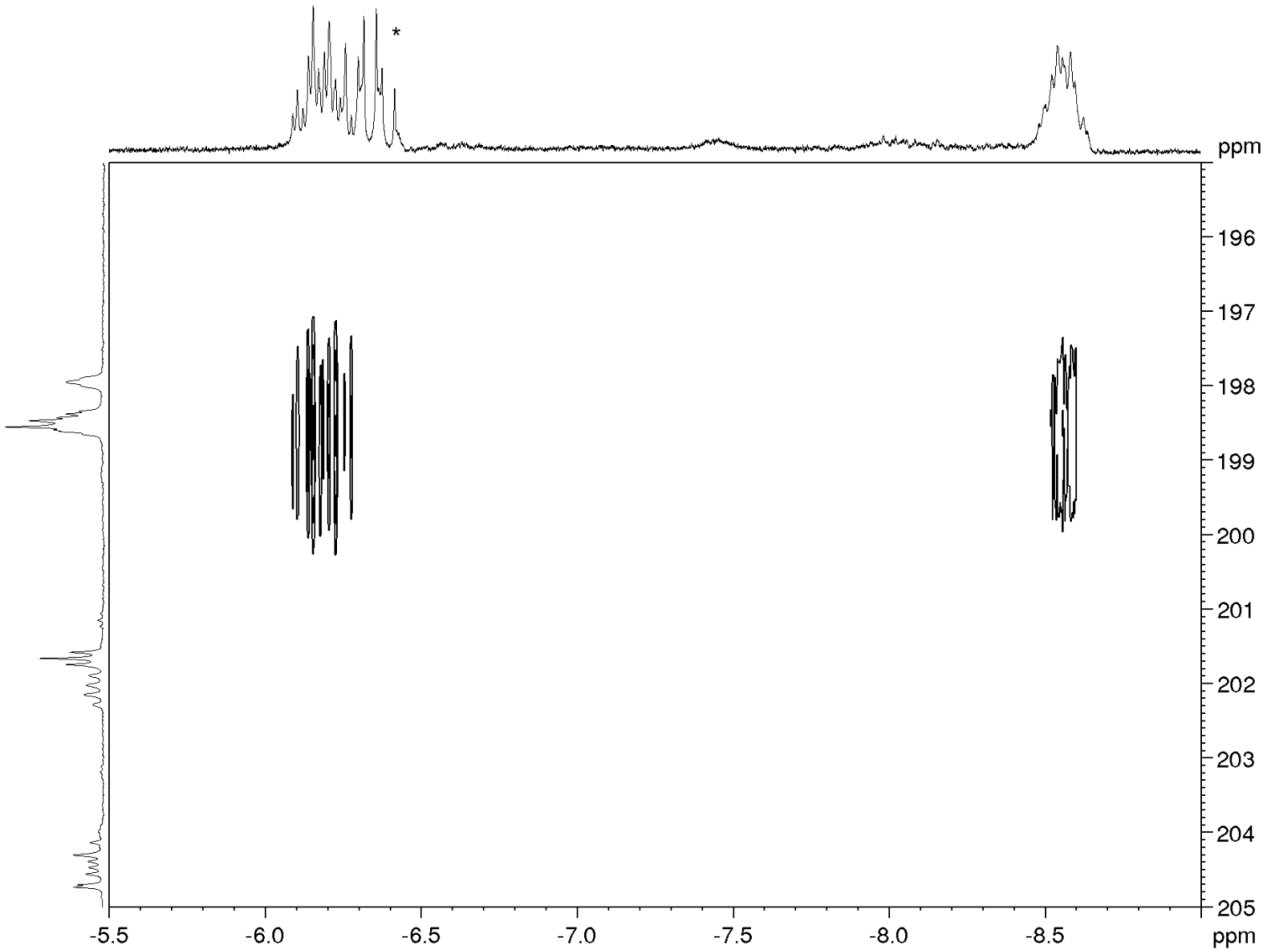


**Figure S14.** $^{13}C$-$^{1}H$ HMBC spectrum ($C_6D_6$, 298 K) of [Ru(AlMePhos)($^{13}CO$)$_2$(μ-H)H] (**2-$^{13}$CO**) showing correlation of both hydride resonances to the broad, multiplet carbonyl resonance at ca. δ 198.5. * = [Ru($PPh_3$)$_2$($^{13}CO$)$_2$$H_2$].

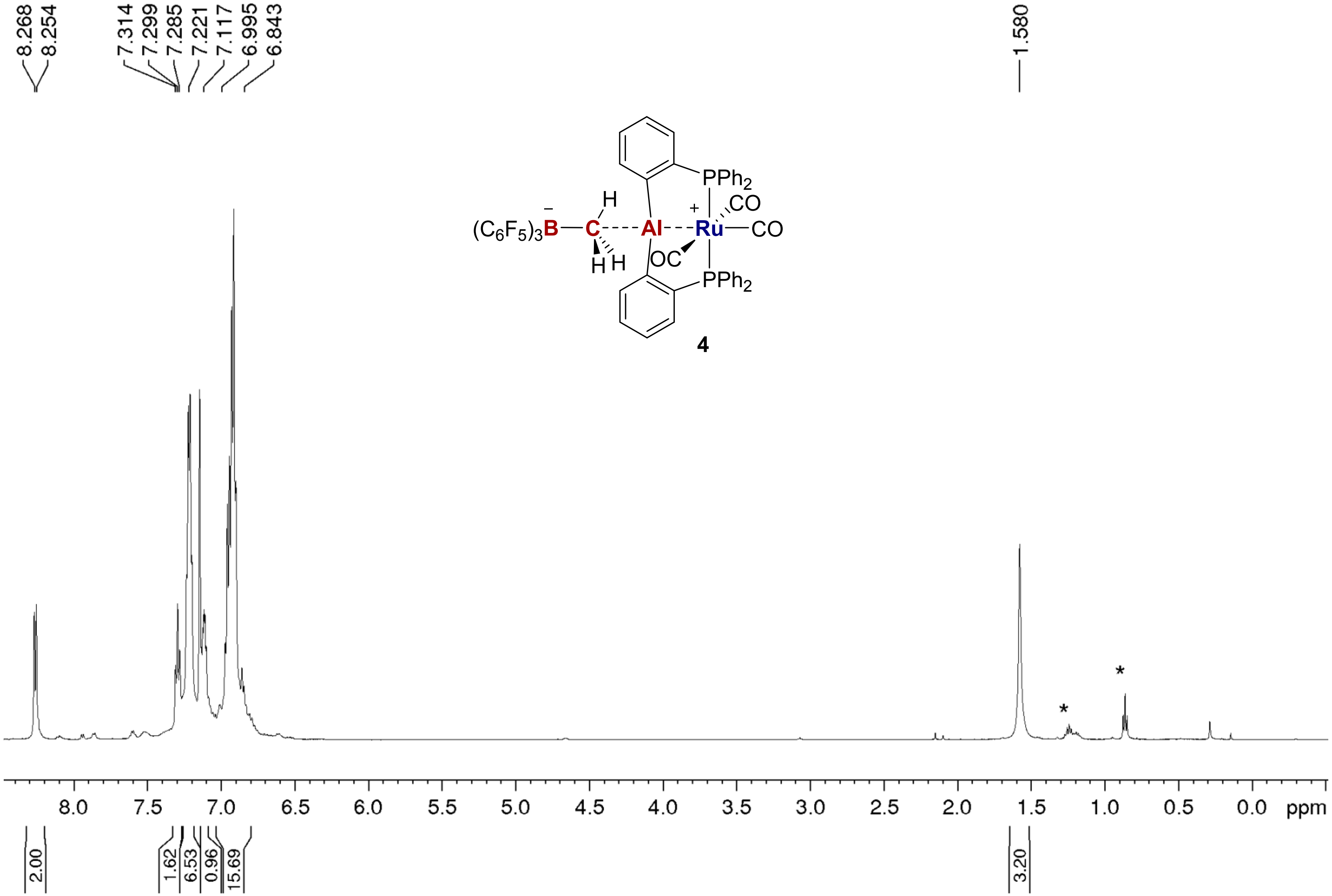


**Figure S15.** $^{1}$H NMR spectrum (500 MHz, $C_6D_6$, 298 K) of [Ru(AlPhos)$(CO)_3$][MeB$(C_6F_5)_3$] (**4**).* denotes hexane.

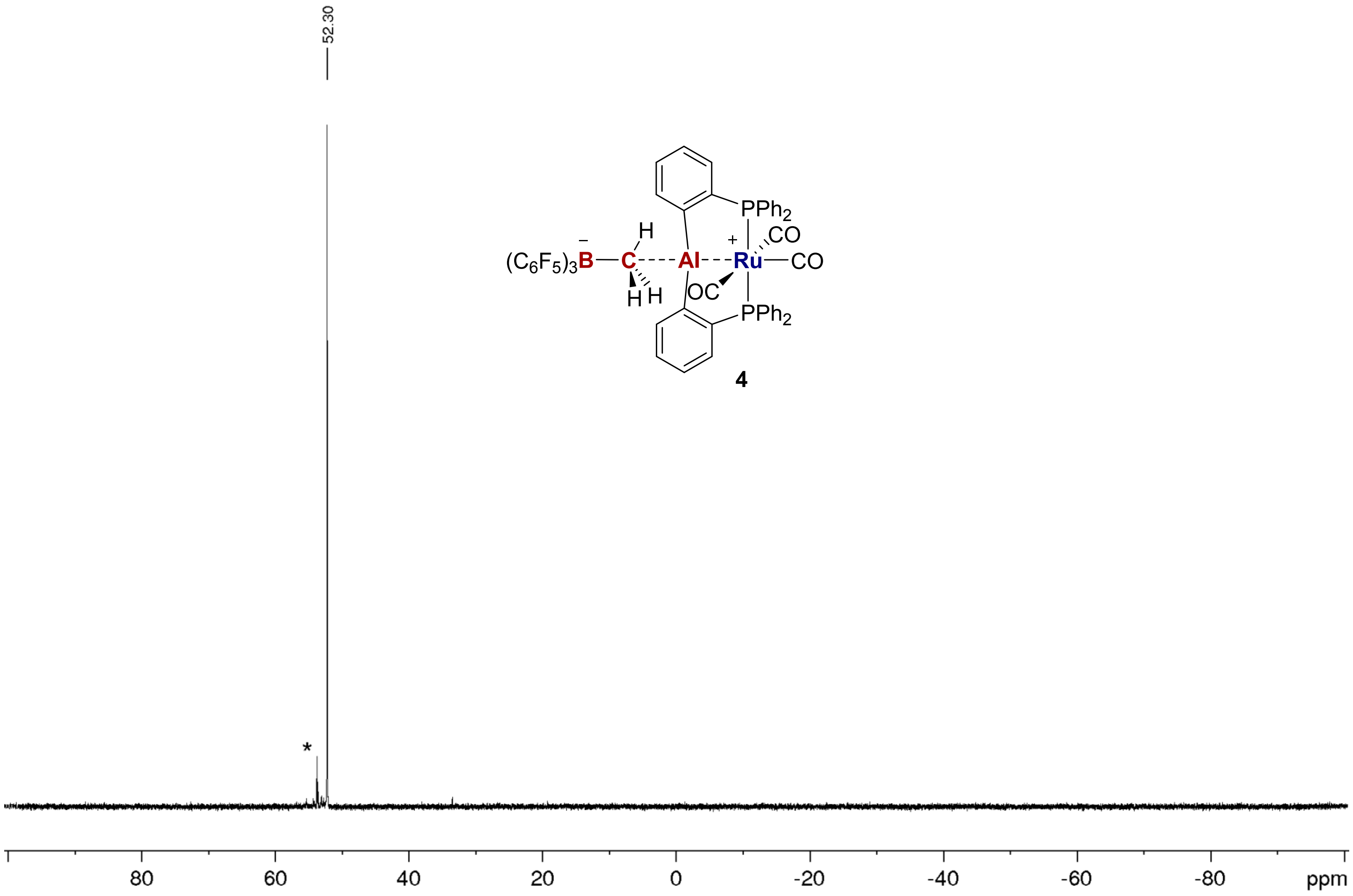


**Figure S16.** $^{31}P\{^1H\}$ NMR spectrum (202 MHz, $C_6D_6$, 298 K) of $[Ru(AlPhos)(CO)_3][MeB(C_6F_5)_3]$ (**4**). The identity of the species that affords the small singlet with * is unknown.

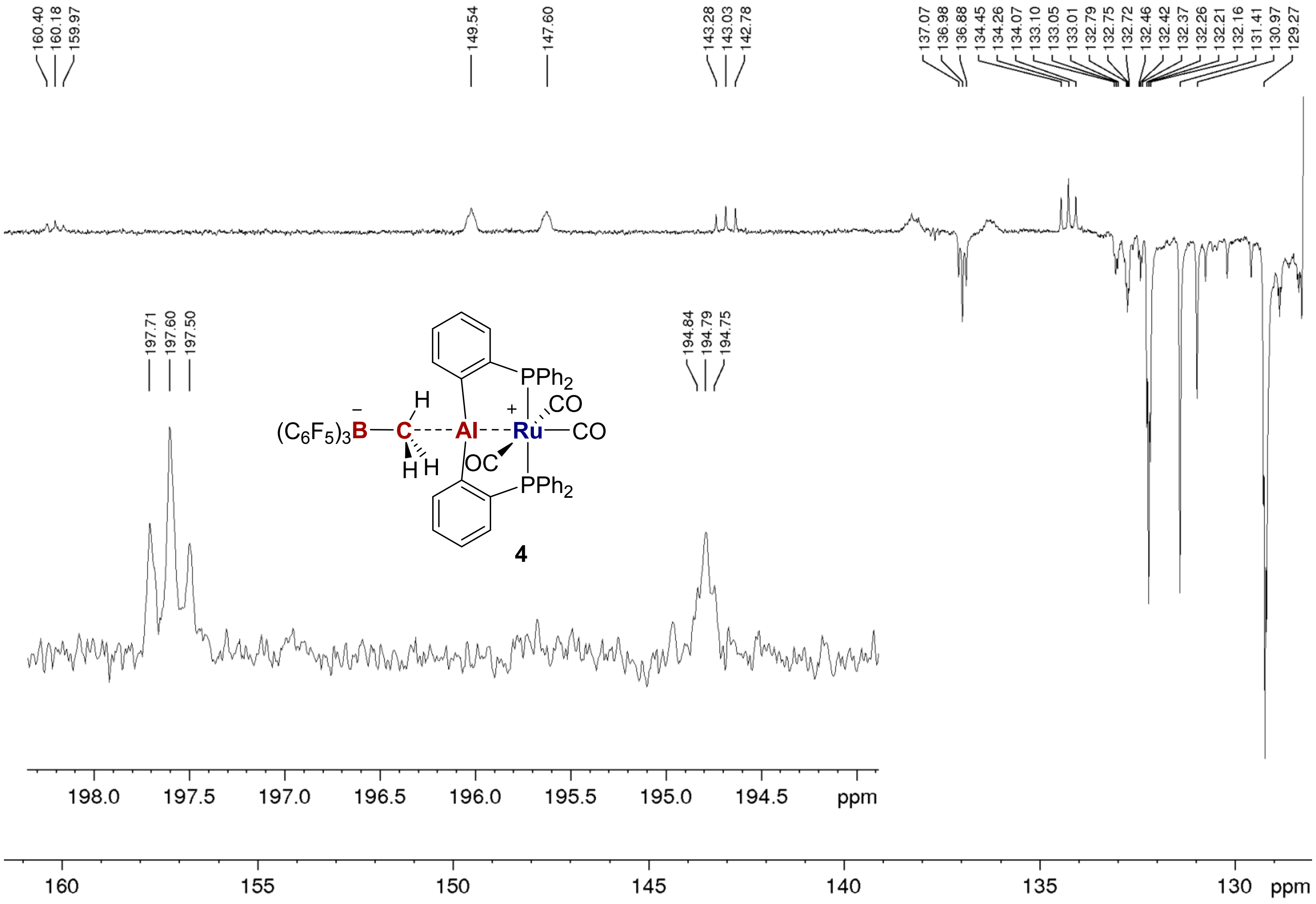


**Figure S17.** Aromatic region (with, inset, Ru-*C*O region) of the $^{13}C\{^{1}H\}$ PENDANT NMR spectrum (125 MHz, $C_6D_6$, 298 K) of $[Ru(AlPhos)(CO)_3][MeB(C_6F_5)_3]$ (**4**).

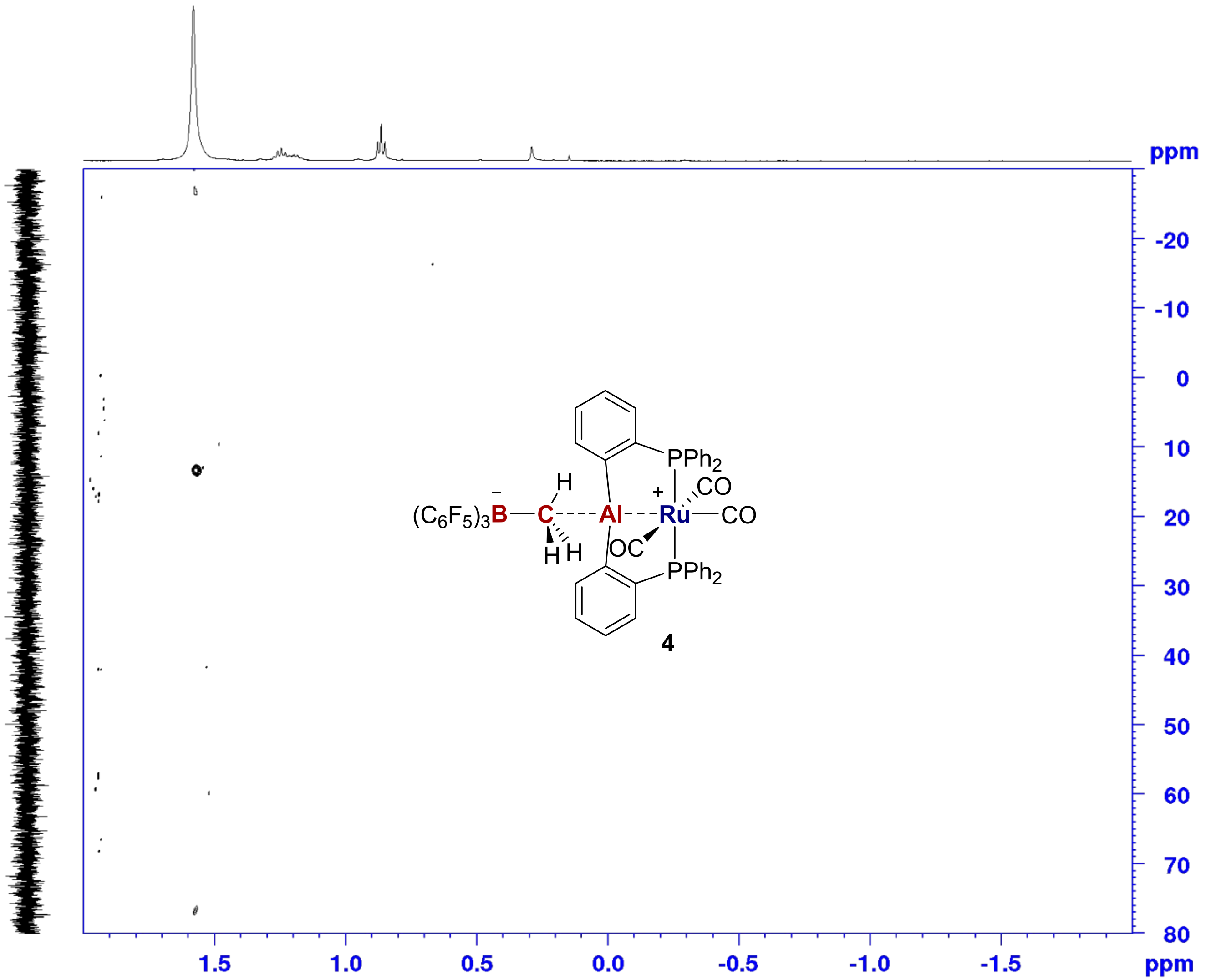


**Figure S18.** $^{1}H$-$^{13}C$ HSQC spectrum ($C_6D_6$, 298 K) showing B-*Me* correlation signal in $[Ru(AlPhos)(CO)_3][MeB(C_6F_5)_3]$ (**4**).

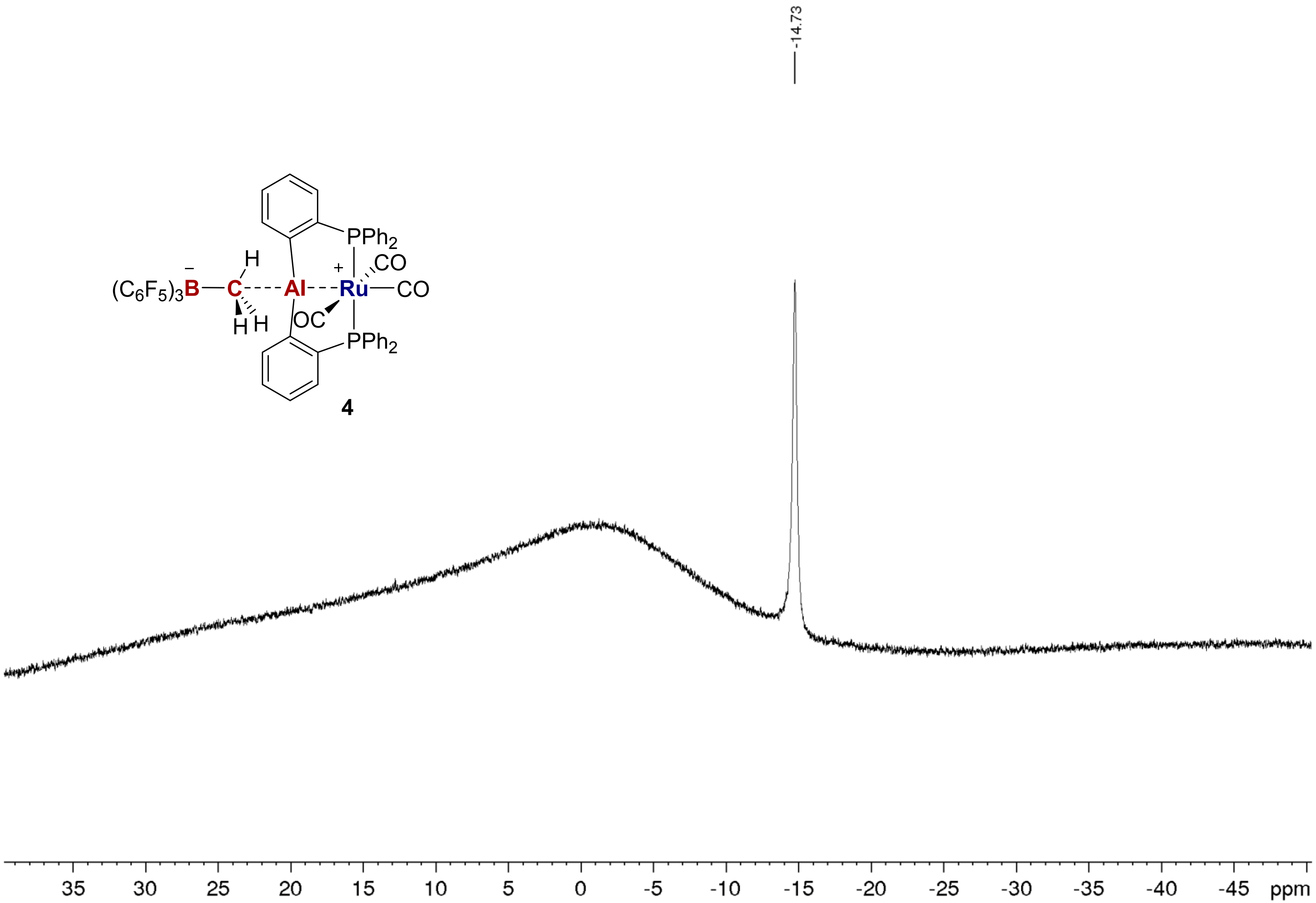


**Figure S19.** $^{11}$B NMR spectrum (160 MHz, $C_6D_6$, 298 K) of [Ru(AlPhos)(CO)$_3$][MeB($C_6F_5$)$_3$] (**4**).

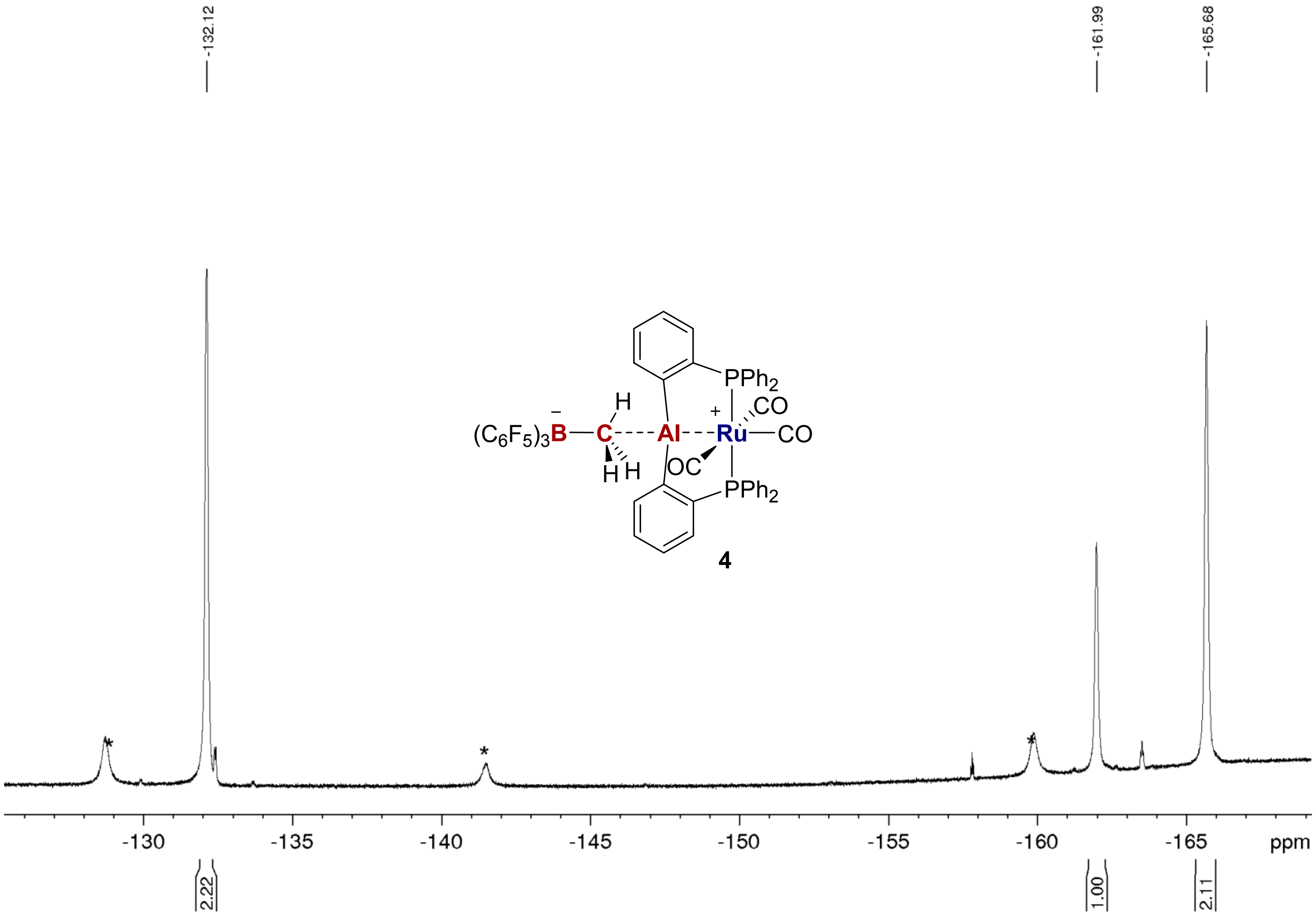


**Figure S20.** $^{19}F\{^{1}H\}$ NMR spectrum (470 MHz, $C_6D_6$, 298 K) of $[Ru(AlPhos)(CO)_3][MeB(C_6F_5)_3]$ (**4**). We associate the resonances denoted * with the solution instability of the complex discussed in the manuscript and illustrated in Figure S22.

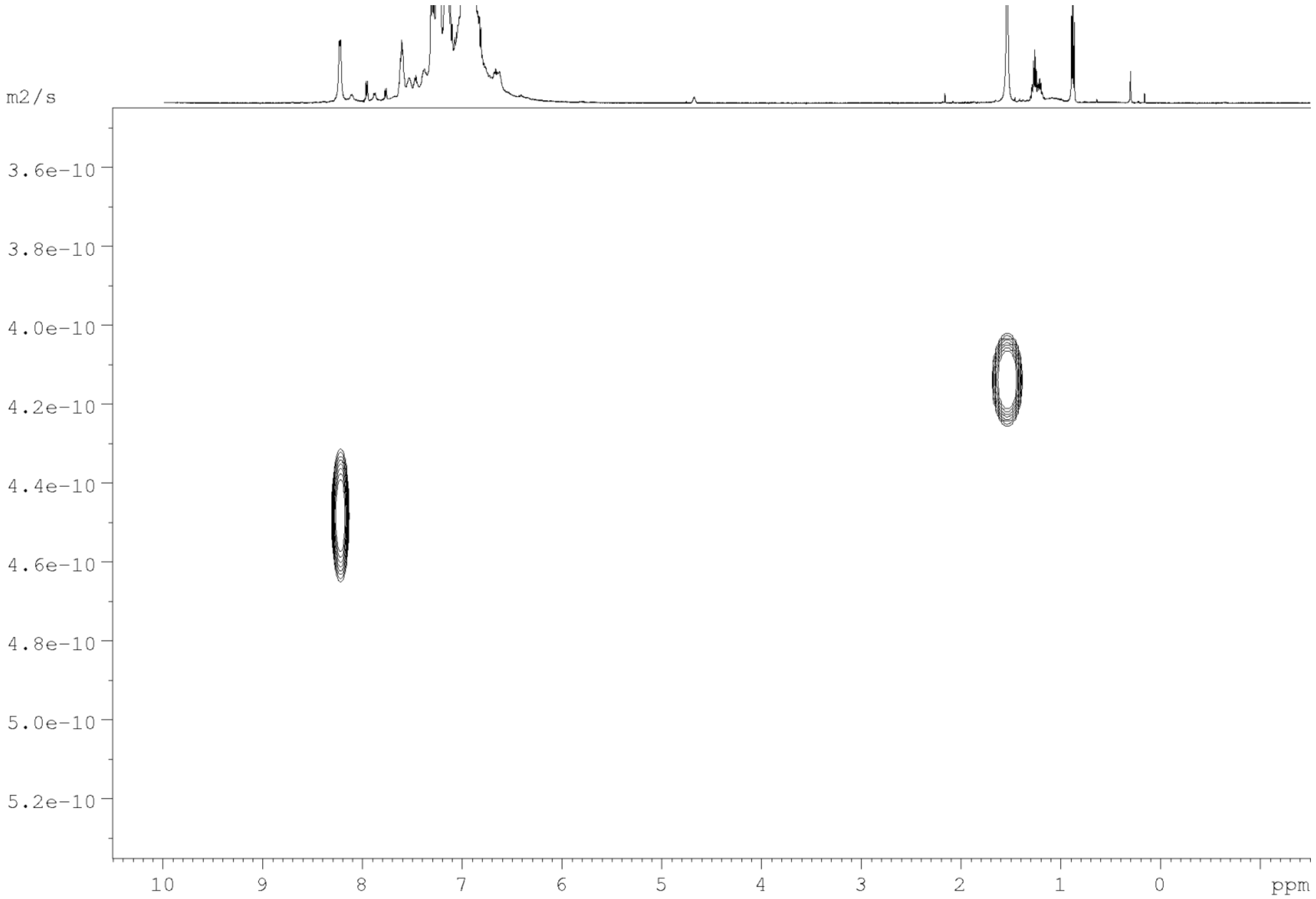


**Figure S21.** $^1$H DOSY NMR spectrum (500 MHz, $C_6D_6$, 298 K) of [Ru(AlPhos)(CO)$_3$][MeB($C_6F_5$)$_3$] (**4**) showing high and low frequency signals of the cation and anion respectively. These yield diffusion constants of 4.5 x $10^{-10}$ and 4.1 x $10^{-10}$ $m^2$/s respectively.

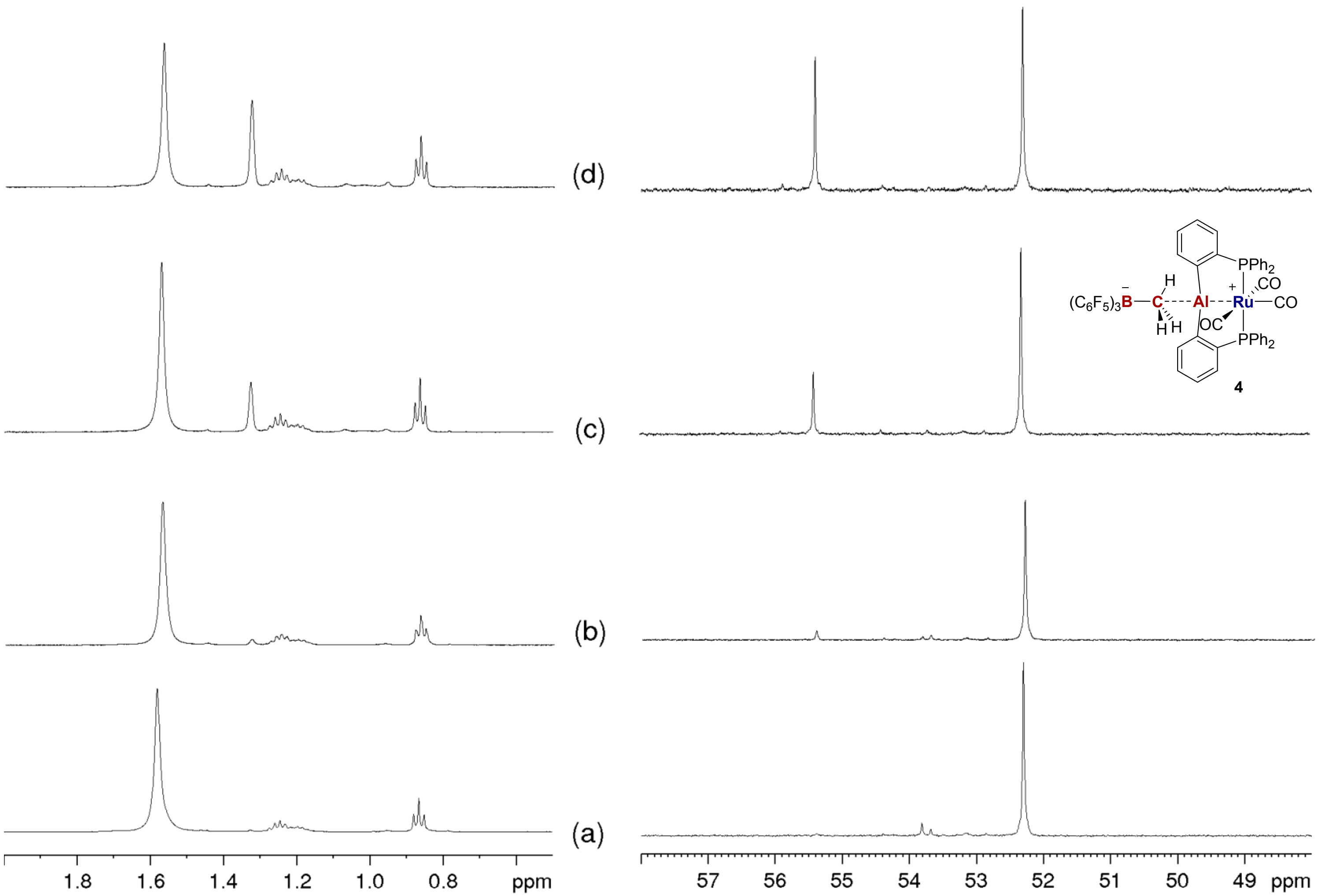


**Figure S22.** (Left) $^{1}H$ (500 MHz) and (right) $^{31}P\{^{1}H\}$ (202 MHz) NMR spectra of (a) a freshly made $C_6D_6$ solution of crystalline $[Ru(AlPhos)(CO)_3][MeB(C_6F_5)_3]$ (**4**), which was then left to stand for (b) 6 h, (c) 12 h and (d) 48 h at room temperature.

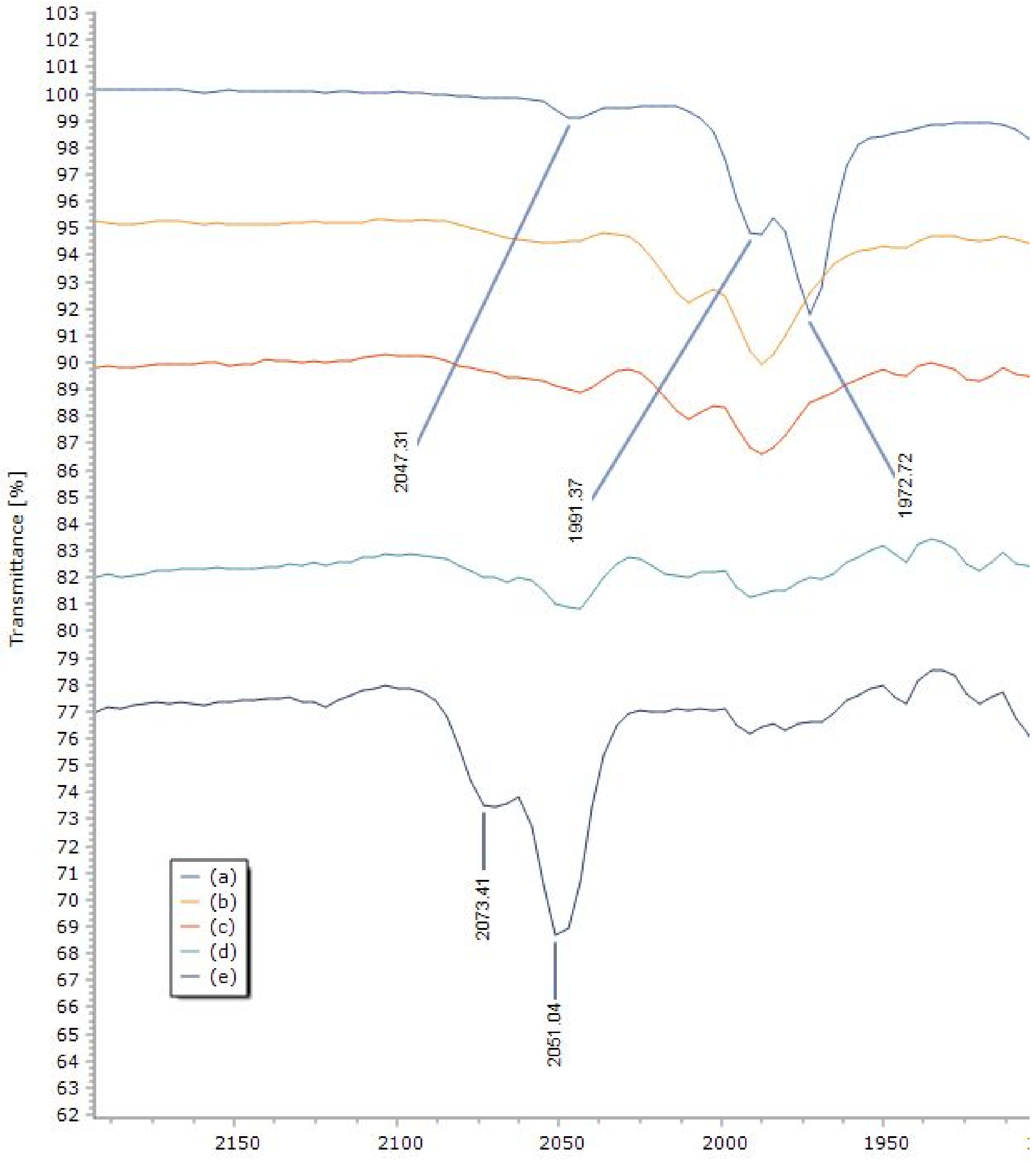


**Figure S23.** In-situ ReactIR spectra ($C_6D_6$) showing (a) the carbonyl bands of [Ru(AlMePhos)$(CO)_3$] (**1**) and then conversion to (e) [Ru(AlPhos)$(CO)_3$][MeB$(C_6F_5)_3$] (**4**) upon addition of B$(C_6F_5)_3$ (1 equiv). Spectra recorded (b) 5 min, (c) 20 min, (d) 50 and (e) 80 min after addition of the borane.

**Table S1.** Crystal data and structural refinement details for compounds **1** and **4**.

| Identification code | **1** | **4** |
|---|---|---|
| Empirical formula | $C_{49}H_{40}AlO_3P_2Ru$ | $C_{116}H_{62}Al_2B_2F_{30}O_6P_4Ru_2$ |
| Formula weight | 866.80 | 2523.25 |
| Crystal system | orthorhombic | orthorhombic |
| Space group | *Pccn* | $P2_12_12_1$ |
| *a*/ Å | 23.2722(2) | 14.9090(2) |
| *b*/ Å | 20.5643(2) | 15.7851(2) |
| *c*/ Å | 17.2834(1) | 22.8503(3) |
| *α*/ ° | 90 | 90 |
| *β*/ ° | 90 | 90 |
| *γ*/ ° | 90 | 90 |
| *U*/ $Å^3$ | 8271.43(12) | 5377.59(12) |
| *Z* | 8 | 2 |
| $\rho_{calc}$/ g $cm^{-3}$ | 1.392 | 1.558 |
| $\mu$/ $mm^{-1}$ | 4.335 | 3.953 |
| *F*(000) | 3560.0 | 2520.0 |
| Crystal size/ $mm^3$ | 0.327 × 0.077 × 0.071 | 0.079 × 0.057 × 0.043 |
| 2θ range for data collection/° | 5.736 to 146.246 | 6.806 to 146.334 |
| Index ranges | $-28 \leq h \leq 28$, $-24 \leq k \leq 25$, $-21 \leq l \leq 17$ | $-13 \leq h \leq 18$, $-19 \leq k \leq 18$, $-22 \leq l \leq 27$ |
| Reflections collected | 62123 | 41291 |
| Independent reflections, $R_{int}$ | 8258, 0.0439 | 10678, 0.0495 |
| Data/restraints/parameters | 8258/96/534 | 10678/262/817 |
| Goodness–of–fit on $F^2$ | 1.049 | 1.076 |
| Final *R*1, *wR*2 [$I >= 2\sigma(I)$] | 0.0366, 0.0996 | 0.0414, 0.1062 |
| Final *R*1, *wR*2 [all data] | 0.0383, 0.1014 | 0.0464, 0.1093 |
| Largest diff. peak/hole/ e $Å^{-3}$ | 0.71/–0.64 | 0.97/–0.42 |
| Flack Parameter | – | –0.004(4) |

## 2. Computational Studies.

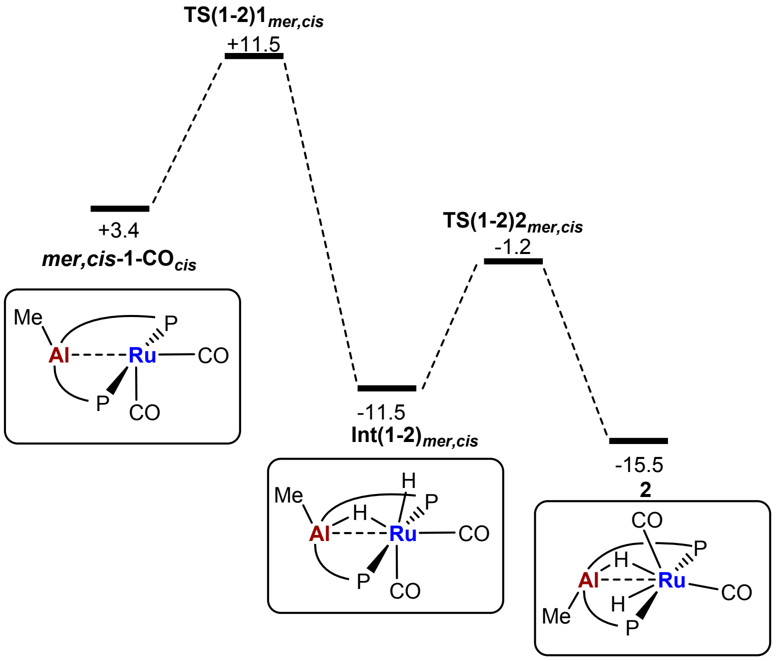


**Figure S24**. Computed reaction profile (kcal/mol) for the addition of $H_2$ to ***mer,cis*-1-CO*cis***.

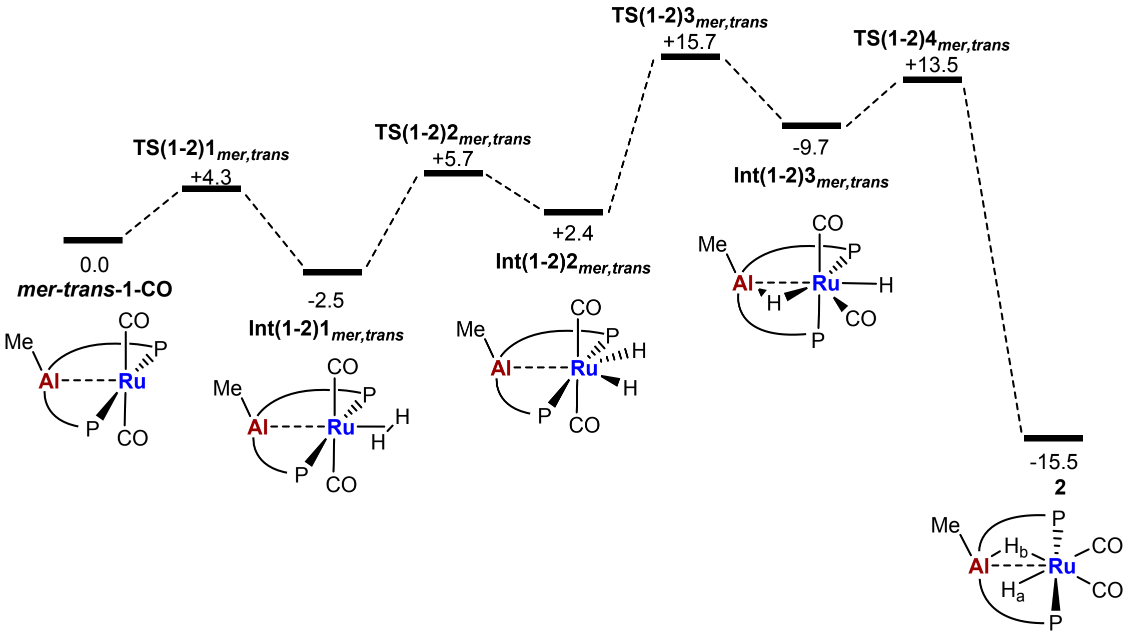


**Figure S25**. Computed reaction profile (kcal/mol) for the addition of $H_2$ to ***mer,trans*-1-CO**.

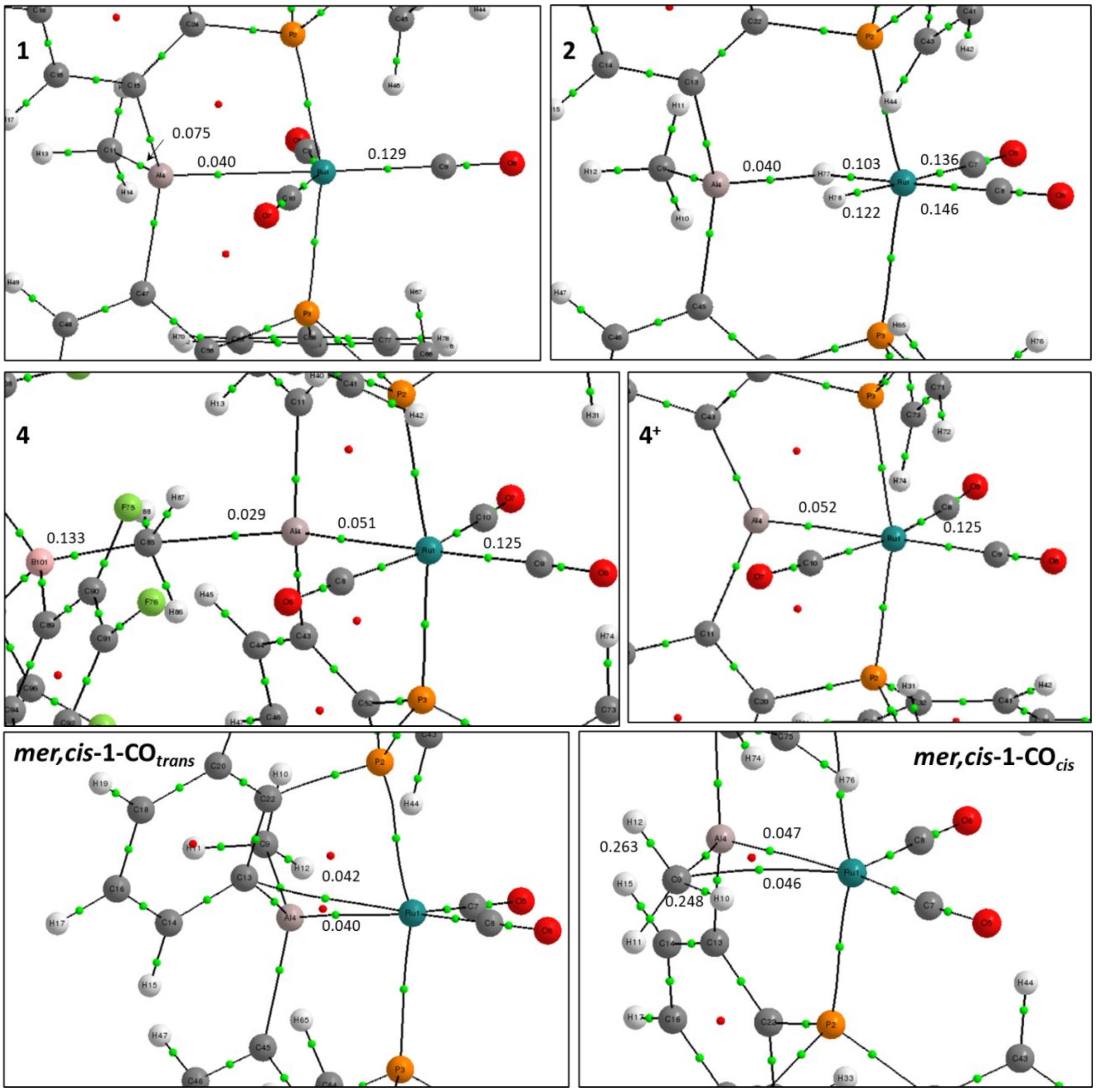


**Figure S26.** Details of the QTAIM molecular graphs of key stationary points. BCPs and RCPs are shown as green and red spheres, respectively with selected BCP r(r) values shown in atomic units.

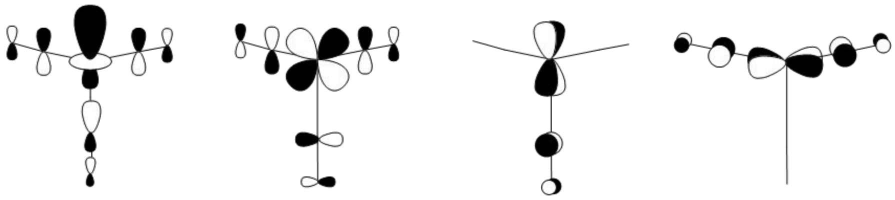

| | Ru$_{HOMO}$ | Ru d$\pi_1$ | Ru d$\pi_2$ | Ru d$\pi_3$ |
|---|---|---|---|---|
| **1** | 1.46 | 1.98 | 1.88 | 1.91 |
| **4** | 1.19 | 1.97 | 1.88 | 1.92 |
| [Ru(ZnPhos)$(CO)_3$] | 1.51 | 1.99 | 1.88 | 1.91 |

**Figure S27.** Schematic representations of the Ru-based occupied orbitals of the $\{Ru(CO)_3\}$ fragment and their occupations within **1**, **4**$^+$ and [Ru(ZnPhos)$(CO)_3$] computed with the ETS-EDA analysis.

**Computed Cartesian Coordinates (Å) and energies (a.u.) for all stationary points.**
SCF, enthalpy and free energies computed with the BP86 functional; wB97x-D energies include a correction for benzene solvent.

**1**
```
SCF  =          -1878.95184174
H(0 K)=         -1878.378539
H(298 K)=       -1878.331534
G(298 K)=       -1878.460785
wB97X-D =       -2788.92926471
Low Freq. = 16.0429cm-1, 24.8780cm-1
```

78
**1**
```
Ru  0.00002  0.51408  0.24669
P   2.37445  0.17588  0.01423
P  -2.37444  0.17587  0.01419
Al  0.00004 -2.05816 -0.68548
O  -0.00002 -1.13732  2.87006
O   0.00005  3.35664  1.49627
O   0.00001  0.96265 -2.82011
C   0.00000 -0.53189  1.86946
C   0.00004  2.29498  1.00026
C   0.00001  0.79658 -1.66005
C  -0.00029 -3.59756  0.60099
H   0.89080 -3.60608  1.25304
H  -0.00054 -4.55111  0.03798
H  -0.89144 -3.60559  1.25297
C   1.75953 -1.96150 -1.71754
C   2.09820 -2.86594 -2.75494
H   1.37675 -3.63798 -3.05324
C   3.32561 -2.80728 -3.43634
H   3.53906 -3.51374 -4.24688
C   4.27852 -1.83776 -3.08424
H   5.23549 -1.78023 -3.61397
C   3.99623 -0.93339 -2.05013
H   4.73555 -0.17272 -1.77733
C   2.75255 -1.00010 -1.38524
C   3.21759 -0.54820  1.50793
C   3.83639 -1.81272  1.46530
H   3.84674 -2.38363  0.53205
C   4.43877 -2.33970  2.62086
H   4.91376 -3.32542  2.57983
C   4.43142 -1.60940  3.81860
H   4.90078 -2.02318  4.71716
C   3.81612 -0.34620  3.86430
H   3.80438  0.22750  4.79689
C   3.20694  0.18124  2.71730
H   2.72142  1.16242  2.76030
C   3.40485  1.69753 -0.32314
C   4.75077  1.77576  0.09699
H   5.19390  0.95476  0.66897
C   5.52335  2.90867 -0.20567
H   6.56470  2.95805  0.12966
C   4.96345  3.97334 -0.92936
H   5.56605  4.85787 -1.16040
C   3.62533  3.90282 -1.34876
H   3.17841  4.73182 -1.90720
C   2.84918  2.77390 -1.04486
H   1.80345  2.73413 -1.36246
C  -1.75941 -1.96142 -1.71764
C  -2.09800 -2.86581 -2.75511
H  -1.37648 -3.63776 -3.05347
C  -3.32540 -2.80720 -3.43652
H  -3.53880 -3.51362 -4.24712
C  -4.27840 -1.83779 -3.08434
H  -5.23538 -1.78031 -3.61406
C  -3.99618 -0.93346 -2.05017
H  -4.73557 -0.17288 -1.77734
C  -2.75250 -1.00011 -1.38529
C  -3.40484  1.69751 -0.32317
C  -4.75080  1.77565  0.09687
H  -5.19392  0.95460  0.66879
C  -5.52342  2.90855 -0.20577
H  -6.56479  2.95786  0.12948
C  -4.96351  3.97329 -0.92934
H  -5.56613  4.85781 -1.16037
C  -3.62534  3.90287 -1.34863
H  -3.17842  4.73194 -1.90696
C  -2.84916  2.77397 -1.04475
H  -1.80340  2.73427 -1.36226
C  -3.21761 -0.54819  1.50789
C  -3.83648 -1.81267  1.46525
H  -3.84684 -2.38359  0.53201
C  -4.43892 -2.33961  2.62081
H  -4.91397 -3.32531  2.57976
C  -4.43156 -1.60930  3.81853
H  -4.90095 -2.02304  4.71710
C  -3.81618 -0.34613  3.86424
H  -3.80443  0.22756  4.79683
C  -3.20694  0.18127  2.71726
H  -2.72136  1.16242  2.76025
```

CO
```
SCF  =          -113.305193181
H(0 K)=         -113.300370
H(298 K)=       -113.297065
G(298 K)=       -113.319529
wB97X-D =       -113.320366850
Low Freq. = 2116.9384cm-1
```

2
CO
```
C   0.00000  0.00000 -0.65707
O   0.00000  0.00000  0.49280
```

H2
```
SCF  =          -1.17646513400
H(0 K)=         -1.166541
H(298 K)=       -1.163236
G(298 K)=       -1.178047
wB97X-D =       -1.17620973246
Low Freq. = 4356.3702cm-1, cm-1
```

2
H2
```
H   0.00000  0.00000  0.37525
H   0.00000  0.00000 -0.37525
```

***mer,cis*-1-CO$_{cis}$**
```
SCF  =          -1765.58374683
H(0 K)=         -1765.018481
H(298 K)=       -1764.974254
G(298 K)=       -1765.097512
wB97X-D =       -2675.55097839
Low Freq. = 14.9134cm-1, 21.7670cm-1
```

76
***mer,cis*-1-CO$_{cis}$**
```
Ru -0.00000 -0.45342 -0.13676
P  -2.36307 -0.11155 -0.05183
P   2.36307 -0.11156 -0.05186
Al  0.00001  2.02081  0.16409
O   0.00008 -3.53901  0.07028
O  -0.00004  0.26911 -3.08208
C   0.00004 -2.35957  0.12091
C  -0.00002  0.02995 -1.92015
C   0.00012  1.00533  1.97691
H   0.00010 -0.10758  2.11753
H  -0.90918  1.33951  2.50361
H   0.90943  1.33950  2.50360
C  -1.82958  2.67373 -0.33998
C  -2.22126  4.00015 -0.63419
H  -1.48784  4.81219 -0.55118
C  -3.52616  4.31988 -1.04874
H  -3.79039  5.35800 -1.27995
C  -4.48643  3.30590 -1.17922
H  -5.50145  3.54270 -1.51599
C  -4.13969  1.97643 -0.88704
H  -4.88723  1.18440 -1.00418
```

C  -2.82947  1.66466 -0.47077
C  -3.08137 -0.37827  1.65288
C  -3.82697  0.61135  2.32470
H  -4.01377  1.57546  1.84215
C  -4.33303  0.36242  3.61280
H  -4.90789  1.14023  4.12665
C  -4.10244 -0.87262  4.23684
H  -4.49644 -1.06358  5.24057
C  -3.36044 -1.86336  3.57044
H  -3.17481 -2.82942  4.05157
C  -2.84685 -1.61749  2.28910
H  -2.25740 -2.38743  1.78067
C  -3.47308 -1.17382 -1.11035
C  -4.79278 -1.48093 -0.71269
H  -5.17392 -1.11777  0.24711
C  -5.61578 -2.26607 -1.53578
H  -6.63626 -2.50070 -1.21476
C  -5.13049 -2.75404 -2.75958
H  -5.77196 -3.37050 -3.39805
C  -3.81662 -2.45803 -3.15635
H  -3.42713 -2.84395 -4.10410
C  -2.98870 -1.67597 -2.33600
H  -1.96196 -1.46296 -2.64422
C   1.82958  2.67372 -0.34008
C   2.22127  4.00013 -0.63429
H   1.48786  4.81218 -0.55128
C   3.52617  4.31985 -1.04885
H   3.79041  5.35797 -1.28005
C   4.48643  3.30586 -1.17934
H   5.50145  3.54265 -1.51612
C   4.13967  1.97639 -0.88716
H   4.88720  1.18436 -1.00432
C   2.82946  1.66464 -0.47087
C   3.47305 -1.17389 -1.11035
C   4.79278 -1.48089 -0.71273
H   5.17395 -1.11764  0.24703
C   5.61578 -2.26608 -1.53578
H   6.63629 -2.50063 -1.21479
C   5.13044 -2.75421 -2.75949
H   5.77191 -3.37070 -3.39794
C   3.81654 -2.45830 -3.15623
H   3.42701 -2.84435 -4.10391
C   2.98862 -1.67619 -2.33592
H   1.96185 -1.46326 -2.64409
C   3.08139 -0.37821  1.65286
C   3.82692  0.61147  2.32466
H   4.01368  1.57557  1.84207
C   4.33296  0.36263  3.61279
H   4.90776  1.14049  4.12663
C   4.10242 -0.87239  4.23687
H   4.49640 -1.06329  5.24062
C   3.36051 -1.86320  3.57049
H   3.17492 -2.82926  4.05166
C   2.84693 -1.61742  2.28912
H   2.25754 -2.38742  1.78071

**TS(*mer,cis*-1-CO$_{cis}$-*mer,trans*-1-CO)**
SCF  =          -1765.57467611
H(0 K)=         -1765.010710
H(298 K)=       -1764.966262
G(298 K)=       -1765.091069
wB97X-D =       -2675.54110029
Low Freq. = -69.4773cm-1, 16.3173cm-1

76
**TS(*mer,cis*-1-CO$_{cis}$-*mer,trans*-1-CO)**
Ru -0.00002 -0.56304 -0.10375
P  -2.34713 -0.17833 -0.04589
P   2.34711 -0.17835 -0.04593
Al -0.00001  2.06032  0.06247
O   0.00005 -3.27867  1.37251
O  -0.00005 -0.16142 -3.09627
C  -0.00002 -2.15874  1.01730
C  -0.00004 -0.19896 -1.91289
C   0.00011  2.42877  2.03667
H  -0.00034  3.52977  2.17344
H   0.89539  2.03325  2.54394
H  -0.89465  2.03248  2.54427
C  -1.76605  2.44468 -0.86645
C  -2.10064  3.67219 -1.48672
H  -1.36930  4.49102 -1.49971
C  -3.34364  3.88267 -2.10980
H  -3.55981  4.84218 -2.59354
C  -4.30446  2.85983 -2.12313
H  -5.27110  3.01278 -2.61507
C  -4.01925  1.63062 -1.50762
H  -4.76460  0.82801 -1.52314
C  -2.76724  1.43351 -0.89111
C  -3.06214 -0.00356  1.67121
C  -3.72398  1.17175  2.08067
H  -3.83265  2.00693  1.38249
C  -4.24214  1.27329  3.38327
H  -4.75097  2.19277  3.69128
C  -4.10781  0.20577  4.28379
H  -4.51045  0.28850  5.29873
C  -3.45208 -0.96978  3.87947
H  -3.34328 -1.80767  4.57610
C  -2.92710 -1.07326  2.58336
H  -2.41433 -1.99117  2.27953
C  -3.45601 -1.46480 -0.81613
C  -4.78305 -1.66317 -0.37844
H  -5.17870 -1.07201  0.45371
C  -5.59505 -2.62609 -0.99912
H  -6.62279 -2.77388 -0.65074
C  -5.09075 -3.39926 -2.05743
H  -5.72448 -4.15272 -2.53680
C  -3.76931 -3.20977 -2.49344
H  -3.36747 -3.81502 -3.31270
C  -2.95296 -2.25054 -1.87445
H  -1.91942 -2.11222 -2.20674
C   1.76605  2.44466 -0.86645
C   2.10065  3.67218 -1.48672
H   1.36936  4.49104 -1.49963
C   3.34361  3.88262 -2.10990
H   3.55979  4.84212 -2.59364
C   4.30438  2.85974 -2.12334
H   5.27098  3.01265 -2.61537
C   4.01917  1.63052 -1.50782
H   4.76449  0.82788 -1.52342
C   2.76721  1.43345 -0.89122
C   3.45592 -1.46489 -0.81616
C   4.78302 -1.66317 -0.37861
H   5.17876 -1.07190  0.45343
C   5.59496 -2.62615 -0.99927
H   6.62275 -2.77388 -0.65100
C   5.09054 -3.39947 -2.05742
H   5.72423 -4.15298 -2.53677
C   3.76905 -3.21007 -2.49328
H   3.36712 -3.81543 -3.31242
C   2.95276 -2.25077 -1.87432
H   1.91916 -2.11252 -2.20648
C   3.06223 -0.00352  1.67112
C   3.72422  1.17175  2.08046
H   3.83292  2.00687  1.38222
C   4.24248  1.27332  3.38302
H   4.75143  2.19277  3.69092
C   4.10810  0.20589  4.28362
H   4.51083  0.28865  5.29853
C   3.45222 -0.96962  3.87942
H   3.34338 -1.80745  4.57613
C   2.92714 -1.07314  2.58336
H   2.41425 -1.99101  2.27964

***mer,trans*-1-CO**
SCF  =          -1765.57978168
H(0 K)=         -1765.015307
H(298 K)=       -1764.970368
G(298 K)=       -1765.096291
wB97X-D =       -2675.55372920
Low Freq. = 16.0654cm-1, 20.6606cm-1

76
***mer,trans*-1-CO**
Ru -0.00000  0.41824 -0.37086

```
P  -2.36124  0.22922 -0.12903
P   2.36123  0.22923 -0.12903
Al -0.00000 -1.67208  1.14129
O  -0.00002 -1.44886 -2.83845
O  -0.00003  1.91196  2.34291
C   0.00000 -0.73281 -1.91569
C   0.00000  1.35850  1.31205
C  -0.00001 -3.46308  0.23678
H   0.00000 -4.27136  0.99411
H   0.89194 -3.61333 -0.39699
H  -0.89196 -3.61333 -0.39698
C  -1.74460 -1.28847  2.14786
C  -2.06072 -1.83278  3.41752
H  -1.33846 -2.49276  3.91611
C  -3.26675 -1.54902  4.08107
H  -3.46444 -1.97810  5.07036
C  -4.21740 -0.70697  3.48152
H  -5.15588 -0.47339  3.99598
C  -3.95704 -0.15874  2.21673
H  -4.69450  0.50307  1.74957
C  -2.73835 -0.44943  1.56858
C  -3.25263 -0.91601 -1.30156
C  -3.82168 -2.12733 -0.86209
H  -3.77239 -2.39946  0.19646
C  -4.45291 -2.98382 -1.78078
H  -4.88940 -3.92455 -1.42913
C  -4.52469 -2.63703 -3.13819
H  -5.01680 -3.30639 -3.85152
C  -3.96042 -1.42815 -3.58113
H  -4.01171 -1.15146 -4.63945
C  -3.32363 -0.57407 -2.66980
H  -2.88391  0.36576 -3.02268
C  -3.36063  1.79924 -0.31032
C  -4.74013  1.77442 -0.61273
H  -5.24585  0.81723 -0.77528
C  -5.46434  2.97204 -0.72118
H  -6.53390  2.93900 -0.95446
C  -4.82067  4.20699 -0.53761
H  -5.38690  5.13992 -0.62737
C  -3.44739  4.24142 -0.24825
H  -2.93661  5.20069 -0.11375
C  -2.72155  3.04486 -0.13827
H  -1.64803  3.07490  0.07515
C   1.74460 -1.28847  2.14786
C   2.06071 -1.83279  3.41751
H   1.33846 -2.49277  3.91611
C   3.26674 -1.54902  4.08107
H   3.46443 -1.97811  5.07035
C   4.21739 -0.70697  3.48152
H   5.15587 -0.47339  3.99598
C   3.95704 -0.15873  2.21673
H   4.69449  0.50308  1.74957
C   2.73834 -0.44943  1.56859
C   3.36062  1.79925 -0.31031
C   4.74012  1.77443 -0.61272
H   5.24584  0.81724 -0.77528
C   5.46434  2.97204 -0.72118
H   6.53390  2.93900 -0.95446
C   4.82066  4.20700 -0.53760
H   5.38690  5.13993 -0.62735
C   3.44739  4.24142 -0.24823
H   2.93661  5.20069 -0.11373
C   2.72154  3.04487 -0.13826
H   1.64803  3.07491  0.07517
C   3.25264 -0.91600 -1.30155
C   3.82169 -2.12732 -0.86208
H   3.77239 -2.39945  0.19647
C   4.45294 -2.98381 -1.78076
H   4.88943 -3.92453 -1.42911
C   4.52473 -2.63702 -3.13818
H   5.01685 -3.30637 -3.85149
C   3.96045 -1.42814 -3.58112
H   4.01175 -1.15145 -4.63944
C   3.32365 -0.57406 -2.66979
H   2.88392  0.36576 -3.02268
```

**TS(*mer,trans*-1-CO-*mer,cis*-1-CO$_{trans}$)**

```
SCF  =          -1765.56912728
H(0 K)=         -1765.005012
H(298 K)=       -1764.960655
G(298 K)=       -1765.084686
wB97X-D =       -2675.53653768
Low Freq. = -76.8947cm-1, 13.1360cm-1
```

76

**TS(*mer,trans*-1-CO-*mer,cis*-1-CO$_{trans}$)**

```
Ru -0.03762  0.34356 -0.43002
P  -2.38754  0.21576 -0.07545
P   2.33442  0.10697 -0.17651
Al -0.10411 -1.89996  0.94656
O  -0.06670 -1.10100 -3.09091
O   0.08563  3.33583  0.39728
C  -0.04987 -0.63513 -2.00457
C   0.02697  2.17562  0.25045
C  -0.71272 -3.61648  0.13518
H  -1.70605 -3.56201 -0.33776
H  -0.76175 -4.39969  0.91721
H   0.00894 -3.96581 -0.62458
C  -1.35198 -0.88593  2.22260
C  -1.21006 -1.01086  3.62632
H  -0.41442 -1.64803  4.03414
C  -2.05402 -0.33276  4.52419
H  -1.91871 -0.45741  5.60480
C  -3.06289  0.51516  4.03927
H  -3.71522  1.05352  4.73540
C  -3.22620  0.68518  2.65440
H  -3.99288  1.36875  2.27434
C  -2.38518 -0.01232  1.76655
C  -3.38662 -1.20972 -0.74254
C  -4.15703 -2.03140  0.10494
H  -4.13871 -1.86770  1.18701
C  -4.93401 -3.06927 -0.43673
H  -5.52482 -3.70689  0.22941
C  -4.94791 -3.29201 -1.82243
H  -5.55001 -4.10476 -2.24190
C  -4.18185 -2.47374 -2.66994
H  -4.18371 -2.64603 -3.75123
C  -3.40327 -1.43727 -2.13453
H  -2.80239 -0.81090 -2.80126
C  -3.52191  1.63781 -0.48998
C  -4.90528  1.56720 -0.20984
H  -5.32632  0.66438  0.24515
C  -5.74801  2.64067 -0.53308
H  -6.81764  2.57809 -0.30655
C  -5.22422  3.78629 -1.15676
H  -5.88520  4.62055 -1.41414
C  -3.85632  3.85197 -1.46147
H  -3.44466  4.73475 -1.96165
C  -3.00796  2.78221 -1.13043
H  -1.94445  2.82795 -1.38174
C   1.80144 -1.99229  1.63081
C   2.20435 -2.93193  2.61373
H   1.47285 -3.64973  3.00908
C   3.51814 -2.98052  3.10827
H   3.79126 -3.71522  3.87459
C   4.48444 -2.08264  2.62343
H   5.50962 -2.10969  3.00840
C   4.12973 -1.14579  1.64241
H   4.87889 -0.43934  1.26761
C   2.80440 -1.11309  1.15189
C   3.28923  1.63132  0.34954
C   4.26014  2.25541 -0.45733
H   4.50019  1.84597 -1.44298
C   4.93126  3.40254  0.00164
H   5.68355  3.87844 -0.63646
C   4.64536  3.93113  1.26884
H   5.17047  4.82378  1.62446
C   3.68225  3.30857  2.08158
H   3.45373  3.71244  3.07345
C   3.00610  2.16908  1.62464
H   2.25812  1.68920  2.26595
C   3.23667 -0.43952 -1.71400
C   4.11842 -1.53832 -1.72631
H   4.30109 -2.10318 -0.80748
```

```
C   4.75861 -1.91605 -2.91865
H   5.43540 -2.77704 -2.91808
C   4.53287 -1.19798 -4.10288
H   5.03371 -1.49529 -5.03015
C   3.65269 -0.10294 -4.09774
H   3.46107  0.45483 -5.02034
C   2.99942  0.26853 -2.91338
H   2.29123  1.10466 -2.91915
```

***mer,cis*-1-CO$_{trans}$**

```
SCF  =             -1765.58436527
H(0 K)=            -1765.019764
H(298 K)=          -1764.974710
G(298 K)=          -1765.101144
wB97X-D =          -2675.55326764
Low Freq. = 16.0515cm-1, 21.3974cm-1
```

76

***mer,cis*-1-CO$_{trans}$**

```
Ru -0.12729  0.11696 -0.48952
P  -2.45866  0.15543 -0.10571
P   2.23530  0.09320 -0.16805
Al -0.03252 -2.09672  0.89773
O  -0.03958 -1.97325 -2.68576
O   0.09291  2.80699 -1.94321
C  -0.06301 -1.23953 -1.75661
C  -0.01617  1.83814 -1.27491
C  -1.05207 -3.80109  0.82955
H  -2.10967 -3.66644  0.55066
H  -1.03257 -4.31042  1.81135
H  -0.59918 -4.49307  0.09677
C  -0.80642 -0.47398  1.96150
C  -0.22134 -0.23073  3.23582
H   0.74038 -0.69983  3.47835
C  -0.85525  0.56291  4.20494
H  -0.40068  0.68666  5.19454
C  -2.07301  1.20451  3.91020
H  -2.56483  1.82587  4.66676
C  -2.64548  1.06827  2.63776
H  -3.55403  1.62182  2.37735
C  -2.02995  0.22669  1.68826
C  -3.57348 -1.29332 -0.41017
C  -4.44650 -1.76316  0.59323
H  -4.45548 -1.28362  1.57761
C  -5.28933 -2.85551  0.33414
H  -5.95791 -3.22362  1.11938
C  -5.27074 -3.47700 -0.92579
H  -5.92643 -4.33122 -1.12414
C  -4.40385 -3.00940 -1.92746
H  -4.38000 -3.49742 -2.90717
C  -3.55235 -1.92323 -1.67150
H  -2.85955 -1.57651 -2.44490
C  -3.54344  1.62615 -0.45405
C  -4.85778  1.48408 -0.94433
H  -5.27333  0.48738 -1.11866
C  -5.63874  2.62091 -1.21126
H  -6.65655  2.49816 -1.59625
C  -5.11951  3.90464 -0.98590
H  -5.72976  4.78918 -1.19616
C  -3.81067  4.05185 -0.49554
H  -3.39544  5.05023 -0.32325
C  -3.02306  2.92116 -0.23783
H  -1.99781  3.04076  0.12631
C   1.91813 -2.23119  1.40414
C   2.42500 -3.25841  2.23902
H   1.75463 -4.06334  2.56774
C   3.76203 -3.27976  2.66998
H   4.11915 -4.08744  3.31932
C   4.64201 -2.25784  2.27561
H   5.68335 -2.26064  2.61567
C   4.18204 -1.22909  1.44026
H   4.86773 -0.43143  1.13310
C   2.83924 -1.22990  1.00366
C   2.97000  1.66463  0.52013
C   4.23999  2.14065  0.13477
H   4.81477  1.60945 -0.63048
C   4.76360  3.30736  0.71683
H   5.74805  3.67265  0.40539
C   4.02777  4.00404  1.68790
H   4.43633  4.91529  2.13727
C   2.76179  3.53341  2.07548
H   2.17961  4.07625  2.82771
C   2.23232  2.37277  1.49210
H   1.23834  2.01454  1.78302
C   3.19921 -0.18636 -1.73890
C   3.97475 -1.34492 -1.94245
H   4.06572 -2.08954 -1.14642
C   4.63130 -1.54894 -3.16831
H   5.22776 -2.45543 -3.31611
C   4.52344 -0.60006 -4.19558
H   5.03595 -0.76163 -5.14973
C   3.74954  0.55645 -3.99802
H   3.65448  1.29921 -4.79701
C   3.08394  0.76124 -2.78114
H   2.47018  1.65711 -2.64451
```

**TS(*mer,cis*-1-CO$_{trans}$-*fac,cis*-1-CO$_{P}$)**

```
SCF  =             -1765.55961053
H(0 K)=            -1764.995523
H(298 K)=          -1764.951166
G(298 K)=          -1765.076732
wB97X-D =          -2675.52705820
Low Freq. = -86.5620cm-1, 9.5252cm-1
```

76

**TS(*mer,cis*-1-CO$_{trans}$-*fac,cis*-1-CO$_{P}$)**

```
Ru -0.08362 -0.11048 -0.95772
P  -2.11707  0.17164  0.05946
P   2.14506  0.19181  0.00176
Al  0.13638 -2.34080  0.70666
O  -0.96302 -2.52408 -2.55381
O  -0.52998  2.27988 -2.83025
C  -0.66955 -1.66615 -1.79295
C  -0.34707  1.44678 -2.01608
C  -0.42400 -4.20503  0.24931
H  -1.48703 -4.26512 -0.04190
H  -0.28951 -4.87412  1.12105
H   0.16724 -4.62913 -0.58154
C  -1.06984 -1.44680  2.09808
C  -0.99742 -1.85814  3.45330
H  -0.29267 -2.65101  3.73639
C  -1.79051 -1.28121  4.45921
H  -1.70356 -1.62978  5.49493
C  -2.69218 -0.25054  4.14236
H  -3.30925  0.20808  4.92257
C  -2.79074  0.19835  2.81786
H  -3.47900  1.01257  2.56626
C  -1.99176 -0.40359  1.81914
C  -3.58360 -0.69613 -0.68179
C  -4.46415 -1.45751  0.11303
H  -4.28388 -1.55616  1.18771
C  -5.56811 -2.09509 -0.47764
H  -6.24301 -2.69271  0.14390
C  -5.80185 -1.97115 -1.85578
H  -6.66106 -2.47237 -2.31354
C  -4.92571 -1.21160 -2.65042
H  -5.09804 -1.12006 -3.72770
C  -3.81626 -0.58162 -2.06949
H  -3.11868 -0.01307 -2.69338
C  -2.67246  1.94609  0.18382
C  -4.00362  2.33413 -0.06950
H  -4.74286  1.58890 -0.37726
C  -4.38380  3.68002  0.06865
H  -5.41953  3.97196 -0.13427
C  -3.44265  4.64425  0.45983
H  -3.74100  5.69281  0.56229
C  -2.11454  4.26156  0.71531
H  -1.37330  5.00952  1.01508
C  -1.72982  2.92078  0.57807
H  -0.68956  2.63038  0.76046
C   2.05860 -2.22689  1.36732
C   2.64420 -3.25121  2.15427
H   2.08533 -4.17914  2.33299
C   3.91989 -3.11385  2.72713
H   4.33643 -3.92080  3.34135
```

C   4.66396 -1.93868  2.52120
H   5.65691 -1.82594  2.96998
C   4.13309 -0.91156  1.72822
H   4.71302  0.00151  1.55244
C   2.85166 -1.07235  1.15801
C   2.82798  1.81796  0.59775
C   3.95531  2.45207  0.03802
H   4.47375  1.99762 -0.81203
C   4.41565  3.66990  0.56712
H   5.29124  4.15547  0.12310
C   3.76269  4.25932  1.66091
H   4.12448  5.20815  2.07045
C   2.64393  3.62685  2.22970
H   2.13325  4.07762  3.08721
C   2.17560  2.41599  1.69908
H   1.30566  1.92232  2.14834
C   2.98907 -0.14543 -1.63293
C   3.62996 -1.38227 -1.87257
H   3.69402 -2.12405 -1.07104
C   4.18476 -1.65711 -3.13117
H   4.68096 -2.61794 -3.30323
C   4.10784 -0.70839 -4.16474
H   4.54180 -0.92844 -5.14559
C   3.46984  0.52182 -3.93703
H   3.40198  1.26553 -4.73770
C   2.90425  0.80149 -2.68357
H   2.40481  1.76062 -2.51635

**_fac,cis_-1-CO$_{P}$**
SCF  =           -1765.56711292
H(0 K)=          -1765.002560
H(298 K)=        -1764.957719
G(298 K)=        -1765.082704
wB97X-D =        -2675.53810271
Low Freq. = 10.2344cm-1, 19.9805cm-1

76
**_fac,cis_-1-CO$_{P}$**
Ru  0.00192 -0.10643 -1.08076
P  -1.86902  0.31194  0.24560
P   2.03619  0.21418  0.10738
Al  0.10514 -2.34268  0.58992
O  -1.32551 -2.31867 -2.71637
O  -0.70107  2.39528 -2.76202
C  -0.86067 -1.49853 -2.01125
C  -0.36925  1.49361 -2.08237
C  -0.17545 -4.19696 -0.11546
H  -1.19449 -4.34770 -0.51209
H  -0.02481 -4.94577  0.68609
H   0.52888 -4.45039 -0.92841
C  -1.22305 -1.74683  2.01277
C  -1.38704 -2.45255  3.23189
H  -0.85168 -3.39911  3.38247
C  -2.20171 -1.97361  4.27040
H  -2.29707 -2.54278  5.20252
C  -2.88760 -0.75517  4.12269
H  -3.51356 -0.36848  4.93426
C  -2.76644 -0.03136  2.92848
H  -3.30151  0.91670  2.80905
C  -1.94773 -0.53809  1.89416
C  -3.45813 -0.18367 -0.59872
C  -4.27916 -1.19926 -0.06984
H  -4.00684 -1.68743  0.87033
C  -5.44589 -1.58829 -0.75018
H  -6.07388 -2.38237 -0.33286
C  -5.80370 -0.96517 -1.95478
H  -6.71290 -1.27074 -2.48305
C  -4.98928  0.05092 -2.48430
H  -5.26092  0.54065 -3.42529
C  -3.81954  0.43727 -1.81529
H  -3.18722  1.22252 -2.24119
C  -2.15657  2.11870  0.62373
C  -3.46014  2.64187  0.76256
H  -4.32933  1.99943  0.59138
C  -3.64806  3.99050  1.10765
H  -4.66434  4.38595  1.20832
C  -2.54107  4.82856  1.31443
H  -2.69056  5.88120  1.57650
C  -1.24178  4.31436  1.17315
H  -0.37259  4.96411  1.31939
C  -1.05069  2.96798  0.82845
H  -0.03924  2.57316  0.69114
C   1.93085 -2.14516  1.50310
C   2.46465 -3.13076  2.37017
H   1.92976 -4.08034  2.50275
C   3.65594 -2.92516  3.08771
H   4.02789 -3.70067  3.76762
C   4.37195 -1.72383  2.94324
H   5.29996 -1.56173  3.50218
C   3.90043 -0.73477  2.06797
H   4.46382  0.19445  1.93145
C   2.69869 -0.96333  1.36555
C   3.07963  1.74739  0.25645
C   4.16368  2.03171 -0.60047
H   4.43280  1.32813 -1.39411
C   4.90690  3.21195 -0.43333
H   5.74454  3.42312 -1.10650
C   4.58583  4.11165  0.59534
H   5.16835  5.02973  0.72377
C   3.51859  3.82708  1.46362
H   3.27004  4.51865  2.27547
C   2.76658  2.65491  1.29348
H   1.93992  2.43739  1.97960
C   2.41957 -0.55907 -1.55122
C   2.79425 -1.93007 -1.69188
H   2.96039 -2.53034 -0.79260
C   2.94980 -2.49646 -2.95444
H   3.24624 -3.54644 -3.04217
C   2.71344 -1.72971 -4.11997
H   2.82666 -2.19030 -5.10686
C   2.35412 -0.38726 -4.01299
H   2.19342  0.22077 -4.90863
C   2.21518  0.21146 -2.73671
H   2.07932  1.29226 -2.66628

**TS(_fac,cis_-1-CO$_{P}$-_fac,cis_-1-CO$_{Al}$)**
SCF  =           -1765.55478374
H(0 K)=          -1764.990664
H(298 K)=        -1764.946322
G(298 K)=        -1765.070022
wB97X-D =        -2675.52955364
Low Freq. = -98.0280cm-1, 14.4802cm-1

76
**TS(_fac,cis_-1-CO$_{P}$-_fac,cis_-1-CO$_{Al}$)**
Ru -0.19360 -0.51620 -1.45252
P   1.83480 -0.04588 -0.11823
C  -1.56629 -1.44252 -2.32839
C   1.69357  1.77350  2.05919
C   1.73010  2.15070  3.41008
H   1.61611  3.20634  3.67770
C   1.91222  1.18209  4.41070
H   1.94211  1.47876  5.46447
C   2.05998 -0.16685  4.05339
H   2.20681 -0.92853  4.82632
C   2.02814 -0.54947  2.70237
H   2.15884 -1.60088  2.43132
C   1.84456  0.41834  1.69403
C   2.50015 -1.78024 -0.15018
C   1.52768 -2.80909  0.02373
Al -0.47082 -2.53923  0.29143
C   1.99643 -4.14386 -0.07673
H   1.28225 -4.97131  0.02060
C   3.35000 -4.44496 -0.30393
H   3.67799 -5.48896 -0.36497
C   4.28102 -3.40654 -0.46648
H   5.33593 -3.63322 -0.65564
C   3.85602 -2.07047 -0.39918
H   4.57788 -1.26131 -0.54908
C   3.22930  1.03923 -0.73473
C   3.19598  1.58044 -2.03407
H   2.33642  1.38851 -2.67958
C   4.25950  2.36550 -2.50975
H   4.21459  2.77941 -3.52251

C 5.36897 2.62037 -1.68996
H 6.19720 3.23418 -2.05936
C 5.41080 2.08848 -0.39011
H 6.27203 2.28441 0.25745
C 4.34772 1.30784 0.08806
H 4.38402 0.91014 1.10696
P -1.74193 0.39840 0.02136
C 0.45411 -0.01199 -3.19792
C -0.75458 2.92590 -0.74808
C -0.41571 4.28014 -0.59694
H -0.01240 4.83634 -1.44977
C -0.59157 4.91549 0.64351
H -0.32944 5.97201 0.76325
C -1.09806 4.18585 1.73077
H -1.22664 4.66960 2.70503
C -1.43409 2.82964 1.58397
H -1.79546 2.27000 2.44968
C -1.27754 2.18816 0.33891
C -3.47633 0.54615 -0.67025
C -4.39095 -0.50676 -0.45509
H -4.10378 -1.36328 0.16315
C -5.67256 -0.46001 -1.02461
H -6.37149 -1.28474 -0.85012
C -6.05654 0.63707 -1.81231
H -7.05734 0.67283 -2.25513
C -5.15177 1.68871 -2.02800
H -5.44447 2.55074 -2.63683
C -3.86741 1.64432 -1.46316
H -3.17376 2.47308 -1.63409
C -2.03928 -0.41678 1.67640
C -2.95229 0.09498 2.62758
H -3.54572 0.98978 2.40873
C -3.12719 -0.56759 3.85075
H -3.82704 -0.16922 4.59342
C -2.41616 -1.75263 4.10865
H -2.55093 -2.27263 5.06419
C -1.56414 -2.28642 3.12869
H -1.06360 -3.24153 3.33613
C -1.35160 -1.63938 1.88541
H 1.56233 2.54007 1.28881
H -0.60215 2.41934 -1.70816
O -2.37931 -2.07175 -2.89689
O 0.59568 0.36502 -4.30128
C -1.50646 -4.15639 -0.26140
H -2.58589 -3.94574 -0.35679
H -1.16536 -4.57534 -1.22402
H -1.40827 -4.95546 0.49984

***fac,cis*-1-CO$_{Al}$**

SCF = -1765.58081500
H(0 K)= -1765.015912
H(298 K)= -1764.971107
G(298 K)= -1765.096198
wB97X-D = -2675.56062514
Low Freq. = 11.8723cm-1, 20.5599cm-1

76
fac_cis_1_CO_P_ts_crup_2_ircf
Ru -0.12319 -0.63169 -1.47410
P 1.82180 0.10027 -0.10883
C -1.48442 -1.41122 -2.50750
C 1.58016 2.41617 1.55741
C 1.50629 3.08947 2.78659
H 1.43023 4.18162 2.79809
C 1.52582 2.36795 3.99101
H 1.47029 2.89534 4.94930
C 1.61832 0.96771 3.96071
H 1.63261 0.39559 4.89417
C 1.70383 0.28981 2.73442
H 1.79372 -0.79985 2.71980
C 1.69110 1.00975 1.52133
C 2.75422 -1.44443 0.36055
C 1.94863 -2.59573 0.57219
Al -0.06257 -2.58080 0.20530
C 2.63197 -3.76876 0.97751
H 2.06120 -4.69201 1.14230
C 4.02627 -3.80098 1.15254
H 4.52193 -4.73106 1.45431
C 4.78966 -2.64499 0.92216
H 5.87856 -2.66651 1.03971
C 4.15309 -1.45791 0.52848
H 4.75120 -0.56092 0.33692
C 2.99285 1.19851 -1.06196
C 2.79339 1.37518 -2.44749
H 1.94236 0.88629 -2.93509
C 3.67139 2.17094 -3.20122
H 3.50203 2.29655 -4.27572
C 4.75915 2.80178 -2.57825
H 5.44566 3.42094 -3.16512
C 4.95994 2.64317 -1.19673
H 5.80181 3.13995 -0.70294
C 4.08069 1.85311 -0.44058
H 4.23069 1.75981 0.63952
P -1.83950 0.31067 -0.01405
C 1.12993 -1.57915 -2.52010
C -1.12361 2.85252 -0.89688
C -0.95533 4.24567 -0.84922
H -0.59237 4.78081 -1.73297
C -1.24891 4.94551 0.33300
H -1.11978 6.03213 0.37441
C -1.70048 4.24523 1.46327
H -1.91703 4.78369 2.39195
C -1.87323 2.85225 1.41628
H -2.20107 2.31921 2.31198
C -1.59604 2.14378 0.23154
C -3.60061 0.24136 -0.64922
C -4.31009 -0.97256 -0.52195
H -3.84816 -1.82554 -0.01413
C -5.61010 -1.08997 -1.03519
H -6.14894 -2.03717 -0.92841
C -6.21655 -0.00050 -1.68179
H -7.23166 -0.09382 -2.08150
C -5.51599 1.20838 -1.81134
H -5.98280 2.06483 -2.30942
C -4.21382 1.33134 -1.29898
H -3.68253 2.28274 -1.39867
C -1.95292 -0.58793 1.60460
C -2.86439 -0.17575 2.60487
H -3.53373 0.67539 2.43742
C -2.94665 -0.88912 3.80796
H -3.64529 -0.56811 4.58813
C -2.14330 -2.02740 3.99406
H -2.20541 -2.59161 4.93201
C -1.28836 -2.45745 2.96671
H -0.70663 -3.37568 3.12496
C -1.16217 -1.75991 1.73723
H 1.56087 2.99158 0.62685
H -0.88470 2.30416 -1.81783
O -2.29158 -1.93331 -3.17934
O 1.85391 -2.22008 -3.18454
C -0.77213 -4.36326 -0.36357
H -1.84164 -4.31983 -0.63677
H -0.22601 -4.76203 -1.23728
H -0.68067 -5.11388 0.44540

**TS(1-2)1$_{mer,cis}$**

SCF = -1766.75048261
H(0 K)= -1766.173893
H(298 K)= -1766.127006
G(298 K)= -1766.255435
wB97X-D = -2676.72475465
Low Freq. = -141.1042cm-1, 17.0125cm-1

78
**TS(1-2)1$_{mer,cis}$**
Ru -0.00002 0.53147 -0.00652
P 2.35550 0.13969 -0.01166
P -2.35553 0.13966 -0.01159
Al 0.00006 -2.09402 -0.12523
O -0.00013 3.60449 0.36291
O -0.00002 0.00183 -2.96004
C -0.00008 2.42877 0.39881
C -0.00002 0.10942 -1.77838
C 0.00022 -2.51863 1.85509

```
H  -0.89491 -2.15202  2.38289
H   0.89560 -2.15242  2.38274
H   0.00002 -3.62614  1.92389
C   1.81062 -2.48278 -0.95369
C   2.20168 -3.69009 -1.58116
H   1.47076 -4.50130 -1.69401
C   3.49860 -3.89013 -2.08475
H   3.75715 -4.83409 -2.57827
C   4.46168 -2.87618 -1.96454
H   5.47254 -3.01982 -2.36116
C   4.12054 -1.66634 -1.34101
H   4.86757 -0.86914 -1.26062
C   2.81286 -1.47679 -0.84596
C   3.07246  0.03070  1.70538
C   3.66570 -1.15144  2.19192
H   3.73710 -2.03041  1.54422
C   4.16795 -1.19941  3.50375
H   4.62554 -2.12313  3.87314
C   4.08433 -0.07232  4.33560
H   4.47631 -0.11328  5.35720
C   3.49456  1.10974  3.85468
H   3.42643  1.99296  4.49846
C   2.98469  1.16122  2.54923
H   2.52039  2.08228  2.18030
C   3.43499  1.42462 -0.82632
C   4.74145  1.69946 -0.36863
H   5.13623  1.17623  0.50803
C   5.53558  2.65290 -1.02651
H   6.54651  2.86086 -0.66009
C   5.03529  3.33818 -2.14496
H   5.65461  4.08394 -2.65422
C   3.73539  3.06965 -2.60381
H   3.33568  3.60539 -3.47103
C   2.93627  2.12135 -1.94727
H   1.92046  1.92474 -2.30233
C  -1.81064 -2.48293 -0.95330
C  -2.20177 -3.69038 -1.58048
H  -1.47084 -4.50156 -1.69335
C  -3.49880 -3.89058 -2.08372
H  -3.75741 -4.83464 -2.57703
C  -4.46192 -2.87667 -1.96345
H  -5.47287 -3.02045 -2.35980
C  -4.12071 -1.66671 -1.34021
H  -4.86777 -0.86954 -1.25976
C  -2.81292 -1.47699 -0.84552
C  -3.43508  1.42436 -0.82656
C  -4.74126  1.69984 -0.36848
H  -5.13580  1.17727  0.50867
C  -5.53542  2.65309 -1.02662
H  -6.54612  2.86155 -0.65988
C  -5.03544  3.33750 -2.14574
H  -5.65478  4.08310 -2.65521
C  -3.73583  3.06830 -2.60500
H  -3.33637  3.60334 -3.47277
C  -2.93667  2.12021 -1.94822
H  -1.92110  1.92306 -2.30364
C  -3.07239  0.03105  1.70552
C  -3.66528 -1.15110  2.19246
H  -3.73656 -2.03027  1.54502
C  -4.16734 -1.19883  3.50437
H  -4.62465 -2.12256  3.87408
C  -4.08389 -0.07148  4.33590
H  -4.47572 -0.11226  5.35756
C  -3.49447  1.11059  3.85457
H  -3.42649  1.99400  4.49809
C  -2.98478  1.16182  2.54904
H  -2.52076  2.08290  2.17979
H   0.37689  0.45941  3.04882
H  -0.37680  0.46103  3.04866
```

**Int(1-2)1$_{mer,cis}$**

```
SCF  =           -1766.79105886
H(0 K)=          -1766.209891
H(298 K)=        -1766.164631
G(298 K)=        -1766.291059
wB97X-D =        -2676.76629306
Low Freq. = 15.2819cm-1, 21.4741cm-1
```

78

**Int(1-2)1$_{mer,cis}$**

```
Ru -0.00006  0.60520  0.12363
P   2.34787  0.18768  0.12461
P  -2.34794  0.18753  0.12457
Al  0.00008 -2.23823 -0.65358
O  -0.00020  3.60251  0.76407
O  -0.00003  0.82356 -2.98038
C  -0.00014  2.47549  0.44578
C  -0.00001  0.67088 -1.81989
C  -0.00005 -3.80801  0.58940
H  -0.89280 -3.81821  1.23897
H   0.89270 -3.81846  1.23897
H  -0.00019 -4.75738  0.02017
C   1.78860 -2.01252 -1.59487
C   2.17239 -2.91600 -2.61722
H   1.46791 -3.69452 -2.93981
C   3.42355 -2.85534 -3.25439
H   3.66895 -3.56425 -4.05342
C   4.35677 -1.88185 -2.86820
H   5.33494 -1.82222 -3.35745
C   4.02751 -0.97469 -1.84945
H   4.75263 -0.21007 -1.55051
C   2.76165 -1.03900 -1.22697
C   3.03474 -0.56188  1.68617
C   3.92511 -1.65432  1.64985
H   4.21009 -2.09479  0.68956
C   4.43939 -2.18478  2.84524
H   5.12247 -3.03984  2.80682
C   4.07723 -1.62576  4.08040
H   4.47753 -2.04271  5.01051
C   3.19285 -0.53440  4.12087
H   2.90168 -0.09706  5.08173
C   2.66830 -0.00819  2.93129
H   1.95864  0.82460  2.96448
C   3.48182  1.63548 -0.18829
C   4.66647  1.83588  0.54956
H   4.94165  1.13471  1.34303
C   5.49517  2.93640  0.27209
H   6.41006  3.08416  0.85563
C   5.15341  3.84069 -0.74477
H   5.79947  4.69875 -0.95759
C   3.97611  3.64389 -1.48628
H   3.70043  4.34649 -2.27954
C   3.14214  2.55175 -1.20738
H   2.22282  2.41078 -1.78441
C  -1.78839 -2.01260 -1.59499
C  -2.17206 -2.91603 -2.61743
H  -1.46749 -3.69446 -2.94006
C  -3.42320 -2.85543 -3.25465
H  -3.66850 -3.56430 -4.05375
C  -4.35653 -1.88206 -2.86841
H  -5.33469 -1.82250 -3.35770
C  -4.02741 -0.97495 -1.84957
H  -4.75263 -0.21043 -1.55061
C  -2.76156 -1.03917 -1.22706
C  -3.48209  1.63518 -0.18824
C  -4.66682  1.83530  0.54956
H  -4.94191  1.13402  1.34297
C  -5.49570  2.93570  0.27214
H  -6.41065  3.08325  0.85563
C  -5.15402  3.84014 -0.74462
H  -5.80023  4.69811 -0.95740
C  -3.97664  3.64362 -1.48607
H  -3.70103  4.34636 -2.27923
C  -3.14249  2.55161 -1.20722
H  -2.22309  2.41087 -1.78418
C  -3.03456 -0.56202  1.68621
C  -3.92426 -1.65500  1.65014
H  -4.20882 -2.09599  0.68997
C  -4.43839 -2.18534  2.84565
H  -5.12094 -3.04083  2.80744
C  -4.07672 -1.62565  4.08062
H  -4.47690 -2.04249  5.01084
C  -3.19297 -0.53376  4.12082
H  -2.90220 -0.09593  5.08159
```

```
C  -2.66852 -0.00759  2.93117
H  -1.95919  0.82562  2.96408
H  -0.00035  0.59943  1.77168
H  -0.00006 -0.99311  0.65505
```

**TS(1-2)2*mer,cis***

```
SCF  =            -1766.77648213
H(0 K)=           -1766.196131
H(298 K)=         -1766.150986
G(298 K)=         -1766.277579
wB97X-D =         -2676.74881422
Low Freq. = -233.2482cm-1, 13.2764cm-1
```

78

**TS(1-2)2*mer,cis***

```
Ru  0.00000  0.83227  0.07644
P   2.30547  0.22124  0.04037
P  -2.30547  0.22126  0.04038
Al -0.00000 -2.38924 -0.59723
O   0.00006  3.47508  1.65119
O  -0.00003  1.86210 -2.86046
C   0.00005  2.48743  1.01765
C  -0.00001  1.49056 -1.74883
C   0.00004 -3.61033  0.96691
H  -0.89440 -3.46511  1.59645
H   0.89419 -3.46461  1.59674
H   0.00040 -4.66753  0.63926
C   1.76662 -2.11628 -1.57464
C   2.15927 -3.03836 -2.57542
H   1.51236 -3.89760 -2.79989
C   3.34344 -2.89708 -3.32037
H   3.59488 -3.62617 -4.09890
C   4.19380 -1.81388 -3.06629
H   5.11776 -1.68284 -3.63971
C   3.85759 -0.88785 -2.06514
H   4.53215 -0.04954 -1.86529
C   2.66203 -1.03073 -1.32883
C   2.92839 -0.63429  1.57888
C   3.75119 -1.77700  1.51169
H   4.00895 -2.20777  0.53946
C   4.23270 -2.37089  2.69166
H   4.86256 -3.26455  2.62847
C   3.90592 -1.82462  3.94201
H   4.28044 -2.28999  4.85982
C   3.08981 -0.68246  4.01310
H   2.82685 -0.25362  4.98587
C   2.59732 -0.09395  2.83950
H   1.94119  0.78016  2.89786
C   3.57496  1.57332 -0.19051
C   4.86068  1.49299  0.38697
H   5.13390  0.62505  0.99477
C   5.79106  2.52638  0.19140
H   6.78337  2.45387  0.64910
C   5.45084  3.64775 -0.58188
H   6.17662  4.45433 -0.72935
C   4.17340  3.73466 -1.15832
H   3.89673  4.60878 -1.75688
C   3.23883  2.70682 -0.95983
H   2.24060  2.78842 -1.39856
C  -1.76663 -2.11627 -1.57463
C  -2.15928 -3.03835 -2.57542
H  -1.51238 -3.89760 -2.79988
C  -3.34343 -2.89705 -3.32038
H  -3.59488 -3.62615 -4.09892
C  -4.19378 -1.81384 -3.06632
H  -5.11773 -1.68279 -3.63975
C  -3.85757 -0.88781 -2.06517
H  -4.53212 -0.04949 -1.86533
C  -2.66203 -1.03071 -1.32884
C  -3.57497  1.57333 -0.19049
C  -4.86075  1.49289  0.38686
H  -5.13400  0.62488  0.99454
C  -5.79114  2.52629  0.19132
H  -6.78349  2.45370  0.64891
C  -5.45088  3.64775 -0.58180
H  -6.17666  4.45433 -0.72925
C  -4.17338  3.73477 -1.15810
H  -3.89668  4.60896 -1.75654
C  -3.23880  2.70692 -0.95964
H  -2.24053  2.78859 -1.39827
C  -2.92839 -0.63430  1.57888
C  -3.75122 -1.77699  1.51167
H  -4.00901 -2.20773  0.53943
C  -4.23273 -2.37089  2.69164
H  -4.86262 -3.26452  2.62843
C  -3.90592 -1.82466  3.94200
H  -4.28043 -2.29003  4.85980
C  -3.08977 -0.68251  4.01310
H  -2.82679 -0.25371  4.98587
C  -2.59729 -0.09400  2.83950
H  -1.94113  0.78010  2.89788
H   0.00000  0.13270  1.56511
H  -0.00009 -0.68806 -0.49106
```

**TS(1-2)1*mer,trans***

```
SCF  =            -1766.75617702
H(0 K)=           -1766.180874
H(298 K)=         -1766.132973
G(298 K)=         -1766.266105
wB97X-D =         -2676.73126728
Low Freq. = -53.9106cm-1, 15.9951cm-1
```

78

**TS(1-2)1*mer,trans***

```
Ru  0.00000  0.45769  0.28339
P   2.36235  0.23215  0.07492
P  -2.36235  0.23214  0.07492
Al  0.00000 -1.85230 -0.86781
O   0.00000 -1.00917  3.00796
O   0.00000  1.48098 -2.64013
C   0.00000 -0.44098  1.98751
C   0.00000  1.10832 -1.53094
C   0.00001 -3.47020  0.31911
H   0.00001 -4.39165 -0.29556
H  -0.89190 -3.51451  0.96901
H   0.89192 -3.51451  0.96901
C   1.74496 -1.63998 -1.92212
C   2.06163 -2.38591 -3.08468
H   1.33881 -3.11760 -3.46936
C   3.26918 -2.21712 -3.78369
H   3.46742 -2.80321 -4.68871
C   4.22082 -1.28994 -3.32871
H   5.16070 -1.14599 -3.87265
C   3.95960 -0.54073 -2.17183
H   4.69762  0.18748 -1.81805
C   2.73923 -0.71860 -1.48703
C   3.25190 -0.70480  1.42086
C   3.81381 -1.97547  1.18864
H   3.76125 -2.41956  0.19015
C   4.44174 -2.67073  2.23663
H   4.87267 -3.65923  2.04611
C   4.51723 -2.10346  3.51746
H   5.00678 -2.64758  4.33190
C   3.96001 -0.83471  3.75351
H   4.01420 -0.38614  4.75104
C   3.32640 -0.14054  2.71321
H   2.89224  0.84714  2.90527
C   3.36446  1.80860 -0.01086
C   4.74123  1.83493  0.30344
H   5.24255  0.92044  0.63617
C   5.46860  3.03158  0.20512
H   6.53599  3.03819  0.45026
C   4.83095  4.21576 -0.20002
H   5.39962  5.14877 -0.27154
C   3.46048  4.20090 -0.50401
H   2.95424  5.12204 -0.81124
C   2.73140  3.00524 -0.40702
H   1.66021  2.99932 -0.63314
C  -1.74496 -1.63999 -1.92212
C  -2.06163 -2.38592 -3.08468
H  -1.33881 -3.11761 -3.46936
C  -3.26918 -2.21714 -3.78368
H  -3.46742 -2.80323 -4.68870
C  -4.22082 -1.28996 -3.32871
```

```
H  -5.16070 -1.14601 -3.87265
C  -3.95960 -0.54074 -2.17183
H  -4.69762  0.18747 -1.81804
C  -2.73922 -0.71861 -1.48703
C  -3.36446  1.80859 -0.01086
C  -4.74123  1.83493  0.30344
H  -5.24255  0.92044  0.63618
C  -5.46860  3.03157  0.20511
H  -6.53599  3.03818  0.45026
C  -4.83096  4.21575 -0.20004
H  -5.39963  5.14876 -0.27156
C  -3.46049  4.20090 -0.50403
H  -2.95425  5.12203 -0.81127
C  -2.73140  3.00523 -0.40703
H  -1.66022  2.99931 -0.63316
C  -3.25190 -0.70480  1.42086
C  -3.81381 -1.97547  1.18865
H  -3.76125 -2.41957  0.19016
C  -4.44173 -2.67073  2.23665
H  -4.87267 -3.65923  2.04613
C  -4.51723 -2.10346  3.51747
H  -5.00677 -2.64757  4.33191
C  -3.96000 -0.83470  3.75351
H  -4.01419 -0.38613  4.75105
C  -3.32640 -0.14054  2.71321
H  -2.89223  0.84714  2.90526
H  -0.00011  3.11831  3.02018
H  -0.00006  3.65466  3.54655
```

**Int(1-2)1**$_{mer,trans}$

```
SCF  =           -1766.77528794
H(0 K)=          -1766.195140
H(298 K)=        -1766.149675
G(298 K)=        -1766.275084
wB97X-D =        -2676.75213231
Low Freq. = 16.3846cm-1, 24.1565cm-1
```

78

**Int(1-2)1**$_{mer,trans}$

```
Ru  0.00005  0.36965 -0.56354
P  -2.36727  0.19685 -0.18479
P   2.36735  0.19683 -0.18478
Al  0.00012 -1.29663  1.53776
O   0.00017 -2.33335 -2.07531
O   0.00006  2.40793  1.76038
C   0.00008 -1.30756 -1.51478
C   0.00005  1.65558  0.86117
C   0.00043 -3.27789  1.21348
H   0.00059 -3.81773  2.18043
H   0.89124 -3.61621  0.65487
H  -0.89034 -3.61652  0.65500
C  -1.74768 -0.68942  2.40550
C  -2.07489 -0.91025  3.76666
H  -1.34830 -1.40925  4.42129
C  -3.29725 -0.49872  4.32426
H  -3.50258 -0.67054  5.38736
C  -4.25611  0.14115  3.52176
H  -5.20866  0.47135  3.95015
C  -3.98565  0.36277  2.16352
H  -4.72975  0.86557  1.53636
C  -2.74686 -0.04746  1.62531
C  -3.22231 -1.21406 -1.05263
C  -3.81198 -2.27212 -0.33395
H  -3.79451 -2.26377  0.75991
C  -4.42107 -3.33650 -1.02107
H  -4.87295 -4.15656 -0.45332
C  -4.45017 -3.34961 -2.42350
H  -4.92495 -4.18045 -2.95574
C  -3.86437 -2.29469 -3.14469
H  -3.88128 -2.29978 -4.23962
C  -3.24872 -1.23491 -2.46436
H  -2.78833 -0.41850 -3.03188
C  -3.40928  1.65902 -0.71383
C  -4.76222  1.49885 -1.08570
H  -5.20761  0.49948 -1.10838
C  -5.53880  2.61405 -1.43988
H  -6.58552  2.47380 -1.72989
C  -4.97657  3.90036 -1.42639
H  -5.58262  4.76835 -1.70618
C  -3.63203  4.06808 -1.05810
H  -3.18407  5.06728 -1.04807
C  -2.85248  2.95480 -0.70763
H  -1.80336  3.09422 -0.43091
C   1.74770 -0.68901  2.40567
C   2.07484 -0.90953  3.76688
H   1.34825 -1.40849  4.42156
C   3.29711 -0.49774  4.32449
H   3.50238 -0.66933  5.38764
C   4.25596  0.14207  3.52194
H   5.20843  0.47246  3.95033
C   3.98559  0.36340  2.16363
H   4.72968  0.86615  1.53643
C   2.74688 -0.04706  1.62540
C   3.40949  1.65878 -0.71415
C   4.76246  1.49846 -1.08585
H   5.20780  0.49906 -1.10826
C   5.53911  2.61353 -1.44024
H   6.58586  2.47317 -1.73013
C   4.97693  3.89987 -1.42712
H   5.58304  4.76778 -1.70707
C   3.63236  4.06774 -1.05899
H   3.18445  5.06697 -1.04925
C   2.85274  2.95458 -0.70831
H   1.80360  3.09411 -0.43172
C   3.22213 -1.21442 -1.05235
C   3.81106 -2.27280 -0.33353
H   3.79328 -2.26448  0.76033
C   4.41982 -3.33746 -1.02052
H   4.87113 -4.15775 -0.45267
C   4.44935 -3.35053 -2.42295
H   4.92389 -4.18157 -2.95507
C   3.86431 -2.29528 -3.14429
H   3.88159 -2.30031 -4.23921
C   3.24897 -1.23523 -2.46411
H   2.78926 -0.41846 -3.03170
H   0.43471  1.41718 -1.95499
H  -0.43493  1.41761 -1.95422
```

**TS(1-2)2**$_{mer,trans}$

```
SCF  =           -1766.76643818
H(0 K)=          -1766.187494
H(298 K)=        -1766.142598
G(298 K)=        -1766.267128
wB97X-D =        -2676.73826459
Low Freq. = -415.9051cm-1, 7.1499cm-1
```

78

**TS(1-2)2**$_{mer,trans}$

```
Ru  0.00000  0.00965 -0.75451
P  -2.36115  0.05759 -0.17315
P   2.36115  0.05758 -0.17314
Al -0.00002 -0.70536  1.92569
O   0.00001 -3.07790 -1.01608
O  -0.00001  3.01292 -0.03465
C   0.00000 -1.91493 -0.90750
C  -0.00000  1.87833 -0.32486
C  -0.00015 -2.67122  2.33398
H  -0.00017 -2.82117  3.43134
H   0.89082 -3.19100  1.93890
H  -0.89117 -3.19088  1.93888
C  -1.73753  0.14951  2.54949
C  -2.05784  0.40627  3.90647
H  -1.31055  0.20850  4.68593
C  -3.30602  0.91796  4.29755
H  -3.51067  1.12022  5.35538
C  -4.29629  1.17464  3.33353
H  -5.27201  1.57290  3.63217
C  -4.02716  0.92319  1.98057
H  -4.79304  1.12873  1.22474
C  -2.75799  0.42447  1.60632
C  -3.28719 -1.50781 -0.59120
C  -4.18739 -2.08926  0.32461
H  -4.34355 -1.62837  1.30416
C  -4.87756 -3.26487 -0.01346
```

H  -5.56722 -3.71147  0.71041
C  -4.68309 -3.86664 -1.26615
H  -5.22227 -4.78362 -1.52566
C  -3.78563 -3.29400 -2.18204
H  -3.61973 -3.76210 -3.15794
C  -3.08403 -2.12656 -1.84468
H  -2.36646 -1.69910 -2.55295
C  -3.30386  1.37870 -1.11140
C  -4.02582  1.08935 -2.28681
H  -4.09998  0.05928 -2.64719
C  -4.66981  2.11587 -2.99646
H  -5.23095  1.87286 -3.90501
C  -4.60152  3.44187 -2.54218
H  -5.10415  4.24099 -3.09691
C  -3.89106  3.73657 -1.36750
H  -3.83717  4.76629 -0.99895
C  -3.24674  2.71393 -0.65458
H  -2.70980  2.95698  0.26656
C   1.73755  0.14936  2.54952
C   2.05789  0.40602  3.90651
H   1.31059  0.20822  4.68597
C   3.30609  0.91761  4.29761
H   3.51076  1.11980  5.35545
C   4.29637  1.17431  3.33360
H   5.27210  1.57250  3.63226
C   4.02721  0.92296  1.98063
H   4.79310  1.12851  1.22480
C   2.75801  0.42433  1.60635
C   3.30385  1.37876 -1.11130
C   4.02572  1.08952 -2.28679
H   4.09982  0.05948 -2.64729
C   4.66969  2.11609 -2.99636
H   5.23077  1.87316 -3.90497
C   4.60148  3.44205 -2.54194
H   5.10410  4.24122 -3.09660
C   3.89110  3.73664 -1.36718
H   3.83726  4.76633 -0.99852
C   3.24679  2.71394 -0.65433
H   2.70992  2.95691  0.26686
C   3.28720 -1.50778 -0.59133
C   4.18744 -2.08928  0.32440
H   4.34363 -1.62846  1.30398
C   4.87762 -3.26485 -0.01377
H   5.56732 -3.71149  0.71005
C   4.68312 -3.86654 -1.26650
H   5.22230 -4.78349 -1.52610
C   3.78561 -3.29385 -2.18231
H   3.61970 -3.76188 -3.15825
C   3.08400 -2.12645 -1.84485
H   2.36641 -1.69895 -2.55307
H   0.69195  0.21375 -2.22385
H  -0.69195  0.21380 -2.22384

**Int(1-2)2*mer,trans***
SCF  =            -1766.76742821
H(0 K)=           -1766.186527
H(298 K)=         -1766.141217
G(298 K)=         -1766.267128
wB97X-D =         -2676.74442162
Low Freq. = 10.4700cm-1, 17.8506cm-1

78
**Int(1-2)2*mer,trans***
Ru  0.02117  0.08213 -1.36082
P  -2.09091  0.17968 -0.10757
P   2.14042  0.05715 -0.09008
Al -0.17516 -2.23914  0.49164
O   0.19009 -2.51209 -2.98920
O   0.30821  3.14730 -1.51220
C   0.09945 -1.61461 -2.24366
C   0.16526  1.99894 -1.36330
C  -1.10631 -3.83953 -0.25879
H  -1.32423 -4.53976  0.57148
H  -0.49205 -4.38137 -0.99750
H  -2.07351 -3.59927 -0.73130
C  -1.20526 -1.34412  1.99533
C  -1.16168 -1.77364  3.34220
H  -0.50685 -2.60696  3.62847
C  -1.92088 -1.14127  4.34391
H  -1.84837 -1.48059  5.38366
C  -2.76566 -0.06758  4.01750
H  -3.35652  0.42772  4.79583
C  -2.85245  0.37314  2.68595
H  -3.51234  1.20763  2.42558
C  -2.07072 -0.26258  1.69967
C  -3.46371 -0.89731 -0.79093
C  -4.44863 -1.43606  0.06392
H  -4.39524 -1.25380  1.14136
C  -5.48882 -2.22057 -0.45867
H  -6.24082 -2.63975  0.21815
C  -5.56183 -2.47302 -1.83795
H  -6.37147 -3.08945 -2.24241
C  -4.58694 -1.93825 -2.69494
H  -4.63120 -2.13401 -3.77144
C  -3.54095 -1.15861 -2.17522
H  -2.77530 -0.75525 -2.84523
C  -2.86420  1.88268 -0.15928
C  -3.99806  2.16613 -0.94626
H  -4.47136  1.37073 -1.52936
C  -4.52872  3.46670 -0.98369
H  -5.40908  3.67211 -1.60183
C  -3.93984  4.49409 -0.23139
H  -4.35649  5.50626 -0.26010
C  -2.81137  4.21812  0.55860
H  -2.34303  5.01330  1.14807
C  -2.27280  2.92335  0.59072
H  -1.38853  2.72294  1.20511
C   1.77110 -2.52982  0.90738
C   2.21928 -3.72712  1.52114
H   1.50977 -4.54760  1.69106
C   3.55560 -3.90230  1.91513
H   3.87323 -4.84090  2.38359
C   4.49000 -2.87304  1.70642
H   5.53375 -3.00216  2.01276
C   4.08424 -1.67859  1.09526
H   4.81504 -0.88099  0.92330
C   2.73918 -1.52399  0.69431
C   2.33863  1.29440  1.30154
C   3.56984  1.91339  1.60508
H   4.44522  1.73601  0.97296
C   3.67782  2.77141  2.71120
H   4.63731  3.25190  2.93065
C   2.56453  3.01194  3.53245
H   2.65247  3.68090  4.39498
C   1.34034  2.38770  3.24486
H   0.46918  2.55761  3.88612
C   1.22643  1.53679  2.13376
H   0.27462  1.03846  1.92272
C   3.51111  0.51748 -1.27690
C   4.23752 -0.49223 -1.94282
H   4.07064 -1.54341 -1.68845
C   5.17649 -0.15835 -2.93214
H   5.73267 -0.95444 -3.43831
C   5.40113  1.18514 -3.27095
H   6.13406  1.44377 -4.04220
C   4.67804  2.19595 -2.61695
H   4.84327  3.24722 -2.87553
C   3.73607  1.86607 -1.63015
H   3.17525  2.66511 -1.13525
H   1.04828  0.30723 -2.59180
H  -1.06126  0.35676 -2.53862

**TS(1-2)3*mer,trans***
SCF  =            -1766.74861355
H(0 K)=           -1766.169309
H(298 K)=         -1766.124334
G(298 K)=         -1766.248436
wB97X-D =         -2676.72313481
Low Freq. = -263.6985cm-1, 18.5830cm-1

78
**TS(1-2)3*mer,trans***
Ru  0.00091 -0.29323 -1.53900
P  -1.89018  0.15620 -0.03234

```
P   1.90750  0.19834 -0.01999
Al -0.00762 -2.59516  0.02760
O   1.53068 -2.42588 -3.12617
O  -1.25425  1.64050 -3.57278
C   0.99134 -1.64031 -2.44495
C  -0.81741  0.93889 -2.74209
C  -0.53729 -4.31877 -0.82966
H  -0.56329 -5.14481 -0.09354
H   0.16054 -4.62049 -1.63023
H  -1.54581 -4.25946 -1.27726
C  -1.19866 -1.99035  1.57655
C  -1.28615 -2.76593  2.76094
H  -0.77310 -3.73534  2.81066
C  -1.99052 -2.32417  3.89289
H  -2.02355 -2.94350  4.79671
C  -2.64246 -1.07890  3.87356
H  -3.18249 -0.72210  4.75737
C  -2.60189 -0.29075  2.71449
H  -3.11122  0.67816  2.69688
C  -1.88915 -0.75481  1.58746
C  -3.49454 -0.40576 -0.82175
C  -4.09081 -1.62726 -0.44962
H  -3.64801 -2.23102  0.34797
C  -5.25383 -2.07295 -1.09940
H  -5.70730 -3.02351 -0.79940
C  -5.83220 -1.30668 -2.12281
H  -6.73829 -1.65685 -2.62815
C  -5.24491 -0.08576 -2.49375
H  -5.69102  0.52216 -3.28789
C  -4.08299  0.36426 -1.84844
H  -3.64211  1.32161 -2.14205
C  -2.27585  1.94454  0.34699
C  -3.56696  2.34087  0.76377
H  -4.37233  1.60257  0.83004
C  -3.83182  3.68576  1.06592
H  -4.83759  3.98144  1.38273
C  -2.81568  4.64876  0.95074
H  -3.02614  5.69841  1.18133
C  -1.53408  4.26318  0.52792
H  -0.73841  5.00860  0.42911
C  -1.26658  2.91872  0.22451
H  -0.27099  2.61901 -0.11574
C   1.85816 -2.50329  0.82345
C   2.42983 -3.60569  1.50923
H   1.94205 -4.58752  1.44863
C   3.60074 -3.48557  2.27461
H   4.01660 -4.36082  2.78721
C   4.23382 -2.23635  2.39858
H   5.13536 -2.12844  3.01129
C   3.70579 -1.12390  1.72934
H   4.19751 -0.14887  1.82350
C   2.54826 -1.27402  0.93009
C   1.82495  1.50228  1.32435
C   2.56148  2.70366  1.29973
H   3.23611  2.92107  0.46689
C   2.45103  3.62344  2.35733
H   3.03425  4.55018  2.32779
C   1.61098  3.35287  3.44774
H   1.52858  4.07059  4.27066
C   0.88256  2.15186  3.48207
H   0.22903  1.92584  4.33081
C   0.98718  1.23184  2.42964
H   0.41931  0.29686  2.47147
C   3.36796  0.74283 -1.06874
C   4.53689 -0.03359 -1.18734
H   4.63578 -0.96380 -0.62184
C   5.57717  0.37276 -2.04232
H   6.47273 -0.25102 -2.13166
C   5.47007  1.56112 -2.77819
H   6.28261  1.87496 -3.44149
C   4.30603  2.34098 -2.66973
H   4.20526  3.26615 -3.24682
C   3.25889  1.92824 -1.83302
H   2.34378  2.52944 -1.78436
H   1.07776  0.64894 -2.28930
H  -1.15383 -1.28677 -2.10810
```

**Int(1-2)3**$_{mer,trans}$

```
SCF  =           -1766.78039173
H(0 K)=          -1766.199231
H(298 K)=        -1766.154132
G(298 K)=        -1766.278646
wB97X-D =        -2676.76521902
Low Freq. = 18.5370cm-1, 21.0486cm-1
```

78

**Int(1-2)3**$_{mer,trans}$

```
Ru  0.00945 -0.29973 -1.55092
P  -1.88165  0.21820 -0.06168
P   1.90263  0.15249 -0.02150
Al  0.00832 -2.73080  0.22330
O   2.10766 -1.29224 -3.54594
O  -1.82903 -0.14101 -3.98531
C   1.34706 -0.87883 -2.76115
C  -1.18797 -0.23149 -3.01249
C  -0.50751 -4.59439 -0.28349
H  -0.42232 -5.29499  0.56869
H   0.13381 -4.98777 -1.09264
H  -1.55149 -4.64655 -0.64026
C  -1.13369 -1.86892  1.67609
C  -1.18462 -2.52989  2.93175
H  -0.67743 -3.49713  3.04889
C  -1.84315 -1.98809  4.04733
H  -1.84650 -2.52745  5.00152
C  -2.48954 -0.74600  3.93937
H  -2.99872 -0.30644  4.80405
C  -2.48221 -0.06537  2.71358
H  -2.98420  0.90369  2.63232
C  -1.81524 -0.62578  1.60206
C  -3.48868 -0.43131 -0.77389
C  -3.99715 -1.68522 -0.37902
H  -3.48245 -2.26449  0.39336
C  -5.16753 -2.19146 -0.96785
H  -5.55181 -3.16640 -0.65011
C  -5.84319 -1.45259 -1.95119
H  -6.75660 -1.84858 -2.40712
C  -5.34304 -0.20134 -2.34703
H  -5.86294  0.38336 -3.11313
C  -4.17124  0.30632 -1.76578
H  -3.79405  1.28471 -2.08044
C  -2.30602  2.00515  0.26655
C  -3.60707  2.39133  0.66343
H  -4.40107  1.64223  0.74162
C  -3.89500  3.73739  0.93719
H  -4.90805  4.02329  1.23938
C  -2.89158  4.71294  0.81656
H  -3.11944  5.76339  1.02614
C  -1.59947  4.33720  0.41827
H  -0.81204  5.09108  0.31764
C  -1.30876  2.99173  0.14172
H  -0.30525  2.70176 -0.18089
C   1.91102 -2.52283  0.88022
C   2.51294 -3.60322  1.57631
H   2.03896 -4.59292  1.54083
C   3.69144 -3.45125  2.32440
H   4.12844 -4.31098  2.84545
C   4.30031 -2.18860  2.42418
H   5.20313 -2.05261  3.02937
C   3.74493 -1.09796  1.74082
H   4.21618 -0.11153  1.81879
C   2.58452 -1.27974  0.95249
C   1.76759  1.48453  1.28998
C   2.35942  2.75778  1.16368
H   2.94548  3.00756  0.27478
C   2.22039  3.70862  2.18919
H   2.69335  4.69065  2.08068
C   1.49263  3.39963  3.34833
H   1.38689  4.14200  4.14638
C   0.90903  2.12932  3.48346
H   0.34461  1.87305  4.38577
C   1.04593  1.17540  2.46438
H   0.59443  0.18644  2.58707
C   3.33252  0.74642 -1.08372
C   4.57831  0.08782 -1.10931
```

H   4.74647 -0.79308 -0.48470
C   5.60865  0.54468 -1.94906
H   6.56554  0.01238 -1.96250
C   5.41449  1.66683 -2.76694
H   6.21964  2.02061 -3.41921
C   4.17277  2.32346 -2.75718
H   4.00129  3.18965 -3.40492
C   3.13618  1.85964 -1.93386
H   2.15639  2.34653 -1.97278
H   0.24804  1.29692 -1.81805
H  -0.42109 -1.95563 -1.31696

**TS(1-2)4**$_{mer,trans}$
SCF  =           -1766.74896874
H(0 K)=          -1766.170313
H(298 K)=        -1766.125106
G(298 K)=        -1766.250817
wB97X-D =        -2676.72459763
Low Freq. = -320.4785cm-1, 14.3808cm-1

78
**TS(1-2)4**$_{mer,trans}$
Ru  0.01008 -0.62872 -1.00511
P   2.10847 -0.13969  0.02509
P  -2.10400 -0.09765  0.01497
Al  0.03500  2.34632 -0.85453
O  -1.33920 -0.78966 -3.79495
O   0.83567 -3.55916 -1.51448
C  -0.83622 -0.72202 -2.74260
C   0.54248 -2.44315 -1.31035
C   0.74409  3.40910 -2.39793
H   0.70416  4.49367 -2.17872
H   0.15513  3.24243 -3.31761
H   1.79661  3.16547 -2.62450
C   1.21990  2.39921  0.79868
C   1.24282  3.53584  1.64401
H   0.59991  4.39518  1.41163
C   2.04793  3.60031  2.79527
H   2.02222  4.49045  3.43431
C   2.87816  2.51995  3.13307
H   3.50434  2.55957  4.03103
C   2.90074  1.37997  2.31327
H   3.54466  0.53280  2.57356
C   2.07897  1.33153  1.16672
C   3.47972  0.25920 -1.18032
C   4.57649  1.05795 -0.79234
H   4.61354  1.48582  0.21438
C   5.61232  1.32235 -1.70213
H   6.45456  1.95122 -1.39484
C   5.56470  0.79178 -3.00181
H   6.37174  1.00417 -3.71086
C   4.47403 -0.00125 -3.39296
H   4.42545 -0.40999 -4.40768
C   3.43297 -0.26356 -2.48818
H   2.56670 -0.85879 -2.79540
C   2.82002 -1.53270  1.04122
C   4.05515 -2.13297  0.72557
H   4.63660 -1.77267 -0.12791
C   4.54365 -3.19755  1.50236
H   5.50185 -3.65944  1.24233
C   3.80982 -3.66600  2.60203
H   4.19196 -4.49610  3.20525
C   2.57966 -3.06773  2.92502
H   1.99991 -3.42711  3.78167
C   2.08358 -2.01168  2.14748
H   1.12027 -1.55717  2.40501
C  -1.92496  2.65991 -0.50012
C  -2.46639  3.97033 -0.55214
H  -1.82856  4.80527 -0.87095
C  -3.80150  4.23987 -0.21180
H  -4.18834  5.26397 -0.26623
C  -4.64333  3.19476  0.20597
H  -5.68341  3.39743  0.48373
C  -4.14669  1.88532  0.26464
H  -4.80170  1.07145  0.59493
C  -2.80778  1.62833 -0.10644
C  -2.26333 -0.40752  1.85648
C  -3.38646 -1.03957  2.42641
H  -4.17856 -1.43672  1.78470
C  -3.49055 -1.17575  3.82148
H  -4.36376 -1.67906  4.25032
C  -2.48543 -0.67051  4.65978
H  -2.57008 -0.77666  5.74640
C  -1.37179 -0.02397  4.09755
H  -0.58676  0.38468  4.74242
C  -1.25788  0.10472  2.70475
H  -0.39138  0.61962  2.27543
C  -3.40474 -1.21914 -0.72311
C  -4.37584 -0.74151 -1.62597
H  -4.43969  0.32795 -1.84672
C  -5.26421 -1.63478 -2.25039
H  -6.01026 -1.24877 -2.95299
C  -5.19623 -3.00871 -1.97819
H  -5.89011 -3.70189 -2.46488
C  -4.22533 -3.49326 -1.08399
H  -4.15808 -4.56546 -0.87155
C  -3.32924 -2.60773 -0.46925
H  -2.55942 -2.99713  0.20575
H  -0.07510 -1.22743  0.50683
H   0.52218  0.75918 -1.78102

**TS**$_{exchange}$
SCF  =           -1766.76730366
H(0 K)=          -1766.187261
H(298 K)=        -1766.141942
G(298 K)=        -1766.267805
wB97X-D =        -2676.74122147
Low Freq. = -509.1742cm-1, 14.2476cm-1

78
**TS**$_{exchange}$
Ru -0.00000  0.53428  0.13440
P   2.36871  0.19653 -0.05399
P  -2.36871  0.19653 -0.05399
Al -0.00001 -2.10460 -0.49805
O  -0.00001 -0.81281  2.86711
O  -0.00001  3.45951  1.14450
C  -0.00001 -0.33792  1.79219
C  -0.00000  2.40634  0.62372
C  -0.00003 -3.58457  0.85373
H   0.89013 -3.55692  1.50689
H  -0.00006 -4.56842  0.34606
H  -0.89017 -3.55687  1.50691
C   1.73543 -2.07586 -1.58489
C   2.04968 -3.05607 -2.56022
H   1.32514 -3.85251 -2.77560
C   3.25751 -3.04706 -3.27808
H   3.45277 -3.81449 -4.03625
C   4.21632 -2.05139 -3.02788
H   5.15910 -2.03468 -3.58535
C   3.95827 -1.06983 -2.06005
H   4.70160 -0.28905 -1.86590
C   2.73293 -1.08824 -1.35774
C   3.23604 -0.38545  1.48574
C   3.91070 -1.62096  1.53067
H   3.94400 -2.25685  0.64092
C   4.53781 -2.03594  2.71812
H   5.05600 -3.00015  2.74628
C   4.49922 -1.22203  3.86022
H   4.98740 -1.54897  4.78423
C   3.82770  0.01216  3.81806
H   3.78998  0.64956  4.70758
C   3.19370  0.42823  2.63909
H   2.66263  1.38609  2.61445
C   3.39367  1.67934 -0.55249
C   4.73559  1.82062 -0.13812
H   5.18257  1.07679  0.52862
C   5.49936  2.91769 -0.56930
H   6.53779  3.01775 -0.23614
C   4.93492  3.88239 -1.41858
H   5.53064  4.73931 -1.74981
C   3.60019  3.74898 -1.83429
H   3.14914  4.50205 -2.48885
C   2.83373  2.65671 -1.40056

H   1.78648  2.57326 -1.70922
C  -1.73544 -2.07585 -1.58491
C  -2.04968 -3.05604 -2.56024
H  -1.32515 -3.85249 -2.77563
C  -3.25751 -3.04703 -3.27811
H  -3.45277 -3.81446 -4.03628
C  -4.21633 -2.05137 -3.02790
H  -5.15911 -2.03465 -3.58536
C  -3.95828 -1.06981 -2.06006
H  -4.70161 -0.28904 -1.86590
C  -2.73293 -1.08823 -1.35775
C  -3.39368  1.67934 -0.55247
C  -4.73558  1.82064 -0.13808
H  -5.18256  1.07682  0.52867
C  -5.49935  2.91771 -0.56926
H  -6.53778  3.01778 -0.23608
C  -4.93492  3.88240 -1.41857
H  -5.53064  4.73932 -1.74980
C  -3.60021  3.74897 -1.83430
H  -3.14916  4.50203 -2.48888
C  -2.83374  2.65670 -1.40057
H  -1.78649  2.57323 -1.70925
C  -3.23603 -0.38547  1.48575
C  -3.91063 -1.62101  1.53068
H  -3.94388 -2.25692  0.64094
C  -4.53772 -2.03601  2.71814
H  -5.05586 -3.00025  2.74630
C  -4.49918 -1.22208  3.86023
H  -4.98735 -1.54904  4.78423
C  -3.82773  0.01214  3.81805
H  -3.79005  0.64955  4.70757
C  -3.19374  0.42823  2.63908
H  -2.66273  1.38612  2.61443
H   0.41513  0.67283 -1.63797
H  -0.41513  0.67288 -1.63796

**1. $B(C_6F_5)_3$**
SCF  =           -4087.16982418
H(0 K)=          -4086.446864
H(298 K)=        -4086.367759
G(298 K)=        -4086.572103
wB97X-D =        -4997.49378435
Low Freq. = 7.1664cm-1, 9.0189cm-1

112
**1. $B(C_6F_5)_3$**
Ru  3.24495 -0.10273 -0.24327
P   3.08888  2.28088  0.09017
P   2.87600 -2.46518  0.07524
Al  1.05434 -0.03578  1.38164
O   0.94681  0.03406 -2.31586
O   5.62491 -0.20096 -2.23619
O   4.55500 -0.16689  2.56558
C   1.80005 -0.02228 -1.51823
C   4.74481 -0.16375 -1.46402
C   4.06256 -0.14451  1.50281
C  -0.78648 -0.04858  0.57200
H  -0.90137  0.68902 -0.23968
H  -1.52414  0.20098  1.35658
H  -1.04388 -1.04327  0.16964
C   1.46116  1.72568  2.32468
C   0.87018  2.08929  3.56055
H   0.17115  1.39681  4.04784
C   1.15448  3.30547  4.20386
H   0.69023  3.53921  5.16890
C   2.04108  4.22067  3.61380
H   2.27469  5.16818  4.11106
C   2.63401  3.91369  2.38048
H   3.32865  4.62477  1.92068
C   2.34352  2.68236  1.75353
C   2.04175  3.19714 -1.14799
C   0.89995  3.92503 -0.75958
H   0.60739  3.96367  0.29387
C   0.13721  4.60552 -1.72500
H  -0.74907  5.16642 -1.41367
C   0.50916  4.56419 -3.07713
H  -0.08698  5.09513 -3.82659
C   1.64673  3.83712 -3.46900
H   1.93991  3.79830 -4.52326
C   2.40806  3.15293 -2.51133
H   3.29118  2.58402 -2.82237
C   4.69383  3.23643  0.01874
C   4.71843  4.58821 -0.38939
H   3.79518  5.07932 -0.71178
C   5.92565  5.30541 -0.39532
H   5.93079  6.35198 -0.71770
C   7.11921  4.68382  0.00437
H   8.06037  5.24329 -0.00550
C   7.10250  3.33942  0.40874
H   8.02989  2.84422  0.71456
C   5.89844  2.61861  0.41268
H   5.89834  1.56774  0.71460
C   1.40593 -1.78336  2.37723
C   0.86231 -2.08789  3.64959
H   0.24314 -1.34140  4.16410
C   1.09865 -3.31300  4.29640
H   0.67529 -3.50078  5.28987
C   1.88421 -4.29633  3.67371
H   2.07849 -5.25143  4.17339
C   2.42833 -4.04740  2.40493
H   3.04529 -4.81039  1.91815
C   2.19007 -2.80602  1.77694
C   4.37149 -3.57170 -0.08782
C   4.25115 -4.90236 -0.54392
H   3.27466 -5.28937 -0.85080
C   5.38221 -5.73183 -0.61534
H   5.27599 -6.76070 -0.97506
C   6.64155 -5.24451 -0.23189
H   7.52287 -5.89142 -0.29243
C   6.76805 -3.92190  0.22273
H   7.74770 -3.53185  0.51764
C   5.64115 -3.08878  0.29106
H   5.75105 -2.05571  0.63325
C   1.66296 -3.24853 -1.10055
C   0.47179 -3.84085 -0.63752
H   0.25812 -3.87055  0.43499
C  -0.44271 -4.38905 -1.55338
H  -1.36692 -4.84481 -1.18298
C  -0.17606 -4.35223 -2.93043
H  -0.90155 -4.76155 -3.64006
C   1.01717 -3.77137 -3.39471
H   1.23168 -3.73808 -4.46781
C   1.93145 -3.21904 -2.48694
H   2.85438 -2.75847 -2.85629
B  -4.37142  0.12500 -0.22655
C  -4.16725  1.62002 -0.67707
C  -4.56455  2.08501 -1.95044
C  -3.52914  2.57469  0.14681
C  -4.33415  3.39181 -2.39779
C  -3.25236  3.87994 -0.27795
C  -3.66078  4.28906 -1.55516
C  -4.19825 -1.03909 -1.27077
C  -3.24318 -0.98462 -2.31388
C  -4.97076 -2.22243 -1.22023
C  -3.04072 -2.03337 -3.22032
C  -4.80982 -3.28402 -2.11922
C  -3.82939 -3.18866 -3.11891
C  -4.76373 -0.19494  1.26314
C  -4.27385 -1.32645  1.95498
C  -5.62726  0.64178  2.00602
C  -4.59315 -1.60351  3.29018
C  -5.98922  0.38491  3.33433
C  -5.46170 -0.74374  3.98085
F  -5.21743  1.26182 -2.80108
F  -4.73864  3.79009 -3.61804
F  -3.39096  5.53801 -1.97353
F  -2.58351  4.74242  0.51365
F  -3.11307  2.24565  1.38828
F  -3.43024 -2.18831  1.34812
F  -2.44786  0.09324 -2.46164
F  -2.10213 -1.94885 -4.17945
F  -4.08212 -2.68064  3.91419
F  -5.78824 -1.00134  5.25629
F  -6.83066  1.20287  3.99344

```
F  -6.17661  1.73611  1.43276
F  -5.94069 -2.36675 -0.28969
F  -5.57689 -4.38726 -2.03431
F  -3.64283 -4.20602 -3.97799
```

**TS(1.B($C_6F_5$)$_3$-4)**

```
SCF  =           -4087.15889912
H(0 K)=          -4086.435338
H(298 K)=        -4086.357608
G(298 K)=        -4086.555746
wB97X-D =        -4997.48769167
Low Freq. = -275.1100cm-1, 5.1722cm-1
```

112

**TS(1.B($C_6F_5$)$_3$-4)**

```
Ru  2.97761 -0.10831 -0.21618
P   2.80799  2.28545  0.09488
P   2.59193 -2.48061  0.05393
Al  0.72315 -0.04405  1.21595
O   0.95022  0.04745 -2.55139
O   5.56245 -0.20676 -1.93932
O   4.04852 -0.18477  2.69204
C   1.70235 -0.01922 -1.66077
C   4.60413 -0.16936 -1.27012
C   3.65275 -0.15709  1.59104
C  -1.23764 -0.03782  0.32218
H  -1.05039  0.43896 -0.64157
H  -1.59067  0.58146  1.15052
H  -1.39211 -1.11641  0.35286
C   0.97205  1.72485  2.17058
C   0.27401  2.07734  3.35121
H  -0.45820  1.37838  3.77581
C   0.49178  3.29543  4.01523
H  -0.05555  3.52498  4.93619
C   1.41623  4.21838  3.50050
H   1.59500  5.16933  4.01321
C   2.11785  3.91729  2.32415
H   2.84312  4.63491  1.92656
C   1.89566  2.68389  1.67417
C   1.91955  3.21224 -1.25112
C   0.77529  3.99270 -0.99290
H   0.36387  4.05577  0.01887
C   0.15745  4.69907 -2.04052
H  -0.73003  5.30265 -1.83002
C   0.67329  4.62728 -3.34287
H   0.18738  5.17676 -4.15559
C   1.81183  3.84534 -3.60462
H   2.21622  3.78234 -4.62009
C   2.43231  3.13873 -2.56555
H   3.32035  2.53196 -2.77442
C   4.42245  3.22010  0.20471
C   4.50226  4.57006 -0.20308
H   3.62547  5.06586 -0.63049
C   5.70713  5.27939 -0.07413
H   5.75537  6.32455 -0.39703
C   6.84371  4.65172  0.45945
H   7.78363  5.20505  0.55436
C   6.77255  3.30892  0.86293
H   7.65608  2.80880  1.27267
C   5.57074  2.59595  0.73384
H   5.53250  1.54714  1.03946
C   0.89145 -1.80651  2.20268
C   0.21260 -2.11582  3.40570
H  -0.45979 -1.37412  3.85448
C   0.37867 -3.34576  4.06383
H  -0.15335 -3.54218  5.00109
C   1.22911 -4.32274  3.52191
H   1.36762 -5.28200  4.03166
C   1.90711 -4.06605  2.32093
H   2.57197 -4.82642  1.89759
C   1.73654 -2.82169  1.67724
C   4.10432 -3.57433  0.05058
C   4.04222 -4.89789 -0.43687
H   3.10910 -5.28262 -0.85932
C   5.17764 -5.72309 -0.39014
H   5.11749 -6.74643 -0.77499
C   6.38260 -5.23861  0.14235
H   7.26767 -5.88228  0.17357
C   6.45079 -3.92301  0.62870
H   7.38829 -3.53537  1.04031
C   5.31997 -3.09402  0.58026
H   5.38736 -2.06765  0.95156
C   1.51286 -3.26895 -1.23890
C   0.31037 -3.91791 -0.89526
H   0.00044 -3.97893  0.15218
C  -0.49198 -4.48583 -1.89970
H  -1.42756 -4.98302 -1.62490
C  -0.10208 -4.41143 -3.24543
H  -0.74356 -4.83495 -4.02399
C   1.10300 -3.77466 -3.58971
H   1.41229 -3.71196 -4.63793
C   1.90688 -3.20290 -2.59376
H   2.84110 -2.70133 -2.86953
B  -3.34863  0.08908 -0.07516
C  -3.47256  1.60237 -0.64338
C  -4.11220  1.93230 -1.85539
C  -2.92653  2.70865  0.03692
C  -4.14980  3.23130 -2.39030
C  -2.92602  4.01494 -0.46284
C  -3.53730  4.27970 -1.69458
C  -3.52082 -1.09529 -1.16438
C  -2.77815 -1.10221 -2.36399
C  -4.39210 -2.19368 -1.01860
C  -2.83851 -2.12124 -3.32110
C  -4.48768 -3.23933 -1.95322
C  -3.69749 -3.20800 -3.10797
C  -3.97266 -0.17053  1.39756
C  -3.61736 -1.28981  2.17831
C  -4.90419  0.68940  2.01376
C  -4.09049 -1.52953  3.47403
C  -5.41565  0.48436  3.30670
C  -5.00252 -0.63037  4.04585
F  -4.75212  0.98785 -2.58600
F  -4.76380  3.47241 -3.56633
F  -3.51696  5.52871 -2.20318
F  -2.31436  5.01727  0.21166
F  -2.34031  2.54600  1.25627
F  -2.76324 -2.22575  1.67898
F  -1.93916 -0.07213 -2.64746
F  -2.07900 -2.07582 -4.43555
F  -3.69075 -2.61591  4.16861
F  -5.47783 -0.84166  5.28735
F  -6.30318  1.35001  3.83751
F  -5.38366  1.78051  1.36857
F  -5.22032 -2.29813  0.04906
F  -5.33342 -4.27024 -1.74519
F  -3.75394 -4.21336 -4.00566
```

**4**

```
SCF  =           -4087.16949477
H(0 K)=          -4086.444324
H(298 K)=        -4086.366524
G(298 K)=        -4086.564451
wB97X-D =        -4997.50943710
Low Freq. = 6.0953cm-1, 13.4516cm-1
```

112

**4**

```
Ru  3.08465 -0.09362  0.26427
P   2.75173  2.30785  0.16686
P   2.78621 -2.46407 -0.15069
Al  0.90990 -0.00353 -1.18800
O   1.24530 -0.23896  2.75826
O   5.72290 -0.20558  1.90897
O   4.19695  0.12436 -2.61877
C   1.91017 -0.19404  1.80512
C   4.74118 -0.16182  1.27816
C   3.78655  0.03933 -1.52670
C   0.96416  1.84679 -1.97144
C   0.21122  2.28126 -3.08871
H  -0.53363  1.61490 -3.53769
C   0.37100  3.56468 -3.63733
H  -0.23189  3.86622 -4.49951
C   1.29180  4.46058 -3.07158
```

| | | | |
|---|---|---|---|
| H | 1.42621 | 5.45976 | -3.49889 |
| C | 2.03407 | 4.07984 | -1.94383 |
| H | 2.73967 | 4.78511 | -1.49298 |
| C | 1.86602 | 2.78771 | -1.40356 |
| C | 4.28261 | 3.36933 | 0.22448 |
| C | 4.23811 | 4.67466 | 0.76213 |
| H | 3.30987 | 5.05663 | 1.19749 |
| C | 5.38599 | 5.48327 | 0.75021 |
| H | 5.33981 | 6.49210 | 1.17310 |
| C | 6.58565 | 5.00068 | 0.20366 |
| H | 7.48030 | 5.63159 | 0.19979 |
| C | 6.63628 | 3.70379 | -0.33205 |
| H | 7.56939 | 3.31800 | -0.75498 |
| C | 5.49257 | 2.89105 | -0.31929 |
| H | 5.54627 | 1.87917 | -0.73040 |
| C | 1.69498 | 3.00164 | 1.52989 |
| C | 0.39316 | 3.47874 | 1.27500 |
| H | 0.00192 | 3.49302 | 0.25342 |
| C | -0.40792 | 3.94272 | 2.33298 |
| H | -1.42277 | 4.29316 | 2.12519 |
| C | 0.09076 | 3.94082 | 3.64421 |
| H | -0.53502 | 4.29946 | 4.46758 |
| C | 1.39153 | 3.47392 | 3.90212 |
| H | 1.78325 | 3.47115 | 4.92446 |
| C | 2.19064 | 2.99879 | 2.85275 |
| H | 3.20108 | 2.63017 | 3.06205 |
| C | 0.95410 | -1.74049 | -2.18864 |
| C | 0.18836 | -2.02518 | -3.34466 |
| H | -0.51526 | -1.27640 | -3.72851 |
| C | 0.30571 | -3.24627 | -4.02798 |
| H | -0.29078 | -3.43078 | -4.92789 |
| C | 1.19139 | -4.23004 | -3.55921 |
| H | 1.29063 | -5.18287 | -4.08950 |
| C | 1.95227 | -3.99335 | -2.40513 |
| H | 2.64040 | -4.76321 | -2.04131 |
| C | 1.83153 | -2.75989 | -1.73062 |
| C | 1.83091 | -3.34733 | 1.17302 |
| C | 0.50835 | -3.77700 | 0.95226 |
| H | 0.03512 | -3.64283 | -0.02390 |
| C | -0.21923 | -4.37184 | 1.99667 |
| H | -1.25306 | -4.67964 | 1.81598 |
| C | 0.37176 | -4.55359 | 3.25513 |
| H | -0.19865 | -5.01465 | 4.06761 |
| C | 1.69455 | -4.13131 | 3.47689 |
| H | 2.15915 | -4.26783 | 4.45883 |
| C | 2.42154 | -3.52205 | 2.44496 |
| H | 3.44926 | -3.18824 | 2.62658 |
| C | 4.33751 | -3.48685 | -0.31237 |
| C | 4.33881 | -4.85392 | 0.04370 |
| H | 3.43925 | -5.31056 | 0.46726 |
| C | 5.49618 | -5.62948 | -0.13077 |
| H | 5.48481 | -6.68703 | 0.15245 |
| C | 6.66138 | -5.05237 | -0.65987 |
| H | 7.56391 | -5.65803 | -0.79054 |
| C | 6.66737 | -3.69386 | -1.01409 |
| H | 7.57360 | -3.23429 | -1.42150 |
| C | 5.51367 | -2.91449 | -0.83893 |
| H | 5.53498 | -1.85562 | -1.10994 |
| F | -1.39979 | -1.09345 | 2.34882 |
| F | -2.41409 | -1.59954 | 4.77003 |
| F | -5.04384 | -1.00857 | 5.35166 |
| F | -6.64311 | 0.11854 | 3.40822 |
| F | -5.64290 | 0.63523 | 0.95453 |
| F | -2.35240 | -2.82613 | -0.04653 |
| F | -3.64035 | -4.77336 | -1.35084 |
| F | -5.84488 | -4.20123 | -2.91606 |
| F | -6.70327 | -1.59274 | -3.13538 |
| F | -5.43178 | 0.37949 | -1.86740 |
| C | -1.29053 | -0.10819 | -0.25042 |
| H | -0.97307 | -1.10824 | 0.07112 |
| H | -0.83442 | 0.70960 | 0.33600 |
| H | -1.24345 | 0.04486 | -1.34819 |
| C | -3.46826 | -0.18047 | 1.51731 |
| C | -2.70844 | -0.76177 | 2.54368 |
| C | -3.20837 | -1.04299 | 3.82688 |
| C | -4.54279 | -0.74670 | 4.12649 |
| C | -5.35270 | -0.17342 | 3.13524 |
| C | -4.80529 | 0.08528 | 1.87167 |
| C | -3.77866 | -1.09071 | -0.91835 |
| C | -3.41311 | -2.44649 | -0.82820 |
| C | -4.06949 | -3.49738 | -1.48408 |
| C | -5.18996 | -3.21232 | -2.27429 |
| C | -5.61990 | -1.88407 | -2.38368 |
| C | -4.92392 | -0.86733 | -1.70786 |
| B | -2.98229 | 0.07043 | -0.03821 |
| F | -3.44723 | 2.63848 | 1.58819 |
| F | -3.53373 | 5.10616 | 0.60900 |
| F | -3.31432 | 5.56712 | -2.10855 |
| F | -2.95408 | 3.42165 | -3.81414 |
| F | -2.82510 | 0.92100 | -2.84784 |
| C | -3.21308 | 1.61592 | -0.58238 |
| C | -3.07009 | 1.92482 | -1.94744 |
| C | -3.11375 | 3.21653 | -2.48570 |
| C | -3.28773 | 4.30892 | -1.62473 |
| C | -3.41262 | 4.06697 | -0.25192 |
| C | -3.36978 | 2.74883 | 0.23467 |
